# Highly active and efficient catalysts for alkoxycarbonylation of alkenes

**DOI:** 10.1038/ncomms14117

**Published:** 2017-01-25

**Authors:** Kaiwu Dong, Xianjie Fang, Samet Gülak, Robert Franke, Anke Spannenberg, Helfried Neumann, Ralf Jackstell, Matthias Beller

**Affiliations:** 1 Leibniz-Institut für Katalyse e.V. an der Universität Rostock, Albert-Einstein Straße 29a, Rostock 18059, Germany; 2 Evonik Performance Materials GmbH, Paul-Baumann-Straße 1, Marl 45772, Germany; 3 Lehrstuhl für Theoretische Chemie, Ruhr-Universität Bochum, Bochum 44780, Germany

## Abstract

Carbonylation reactions of alkenes constitute the most important industrial processes in homogeneous catalysis. Despite the tremendous progress in this transformation, the development of advanced catalyst systems to improve their activity and widen the range of feedstocks continues to be essential for new practical applications. Herein a palladium catalyst based on 1,2-bis((*tert*-butyl(pyridin-2-yl)phosphanyl)methyl)benzene **L3** (py^*t*^bpx) is rationally designed and synthesized. Application of this system allows a general alkoxycarbonylation of sterically hindered and demanding olefins including all kinds of tetra-, tri- and 1,1-disubstituted alkenes as well as natural products and pharmaceuticals to the desired esters in excellent yield. Industrially relevant bulk ethylene is functionalized with high activity (TON: >1,425,000; TOF: 44,000 h^−1^ for initial 18 h) and selectivity (>99%). Given its generality and efficiency, we expect this catalytic system to immediately impact both the chemical industry and research laboratories by providing a practical synthetic tool for the transformation of nearly any alkene into a versatile ester product.

## Abstract (web summary)

The carbonylation of alkenes is tremendously important industrial process, but many substrates are highly challenging. Here the authors report a highly active catalytic system for the alkoxycarbonylation of alkenes that is also general across the range of alkene substitution patterns.

Functionalization reactions of alkenes constitute a fundamental basis of today's chemical industry. Hence, in addition to polymerizations and oxidations, carbonylation reactions count among the largest industrial applications in the area of homogeneous catalysis and a large variety of value-added bulk and fine chemicals are available via this technology^1^,^2^,^3^,^4^,^5^. Besides hydroformylations and related transformations^6^,^7^,^8^, which produce over 10 million tons of oxo products every year, alkoxycarbonylation is another important type of such reactions. They have been shown to be core processes for the production of acids, esters and amides^3^,^4^,^5^,^9^. For example, the current state-of-the-art commercial process (Lucite Alpha process) to methyl propionate, a key intermediate for methyl methacrylate polymers, is produced based on the palladium-catalysed methoxycarbonylation of ethylene on a >300,000-ton-per-annum scale^10^,^11^.

Comparing the reactivity of diverse olefins involving transition metal hydride complexes, it is well known that ethylene shows highest activity and terminal olefins react much faster than internal ones. Hence, the rate of the respective functionalization reaction falls with increasing steric hindrance of the substrate^12^,^13^,^14^,^15^,^16^. With respect to catalytic carbonylations the reactivity order of different olefins was found to be independent of the metal Co^17^, Rh^18^ or Pd^19^ (Fig. 1). In fact, the reaction of branched olefins requires significantly more severe reaction conditions or alternatively more active catalysts. Essentially, the carbonylation of tetra- or tri-substituted *sp*^2^-configurated C-atoms is extremely difficult (Keulemans' rule)^20^. Accordingly, catalytic carbonylation reactions are limited to terminal olefins and the n,m-disubstituted internal alkenes R^1^–CH=CH–R^2^. The major problem impeding the development of a general carbonylation of tetra- and tri-substituted alkenes is the low binding affinity of these substrates towards the metal center and the sluggish migratory insertion of the metal complex leading to intrinsically demanding hydrometalation^21^. The resulting tertiary alkylmetal intermediates are highly unstable and are readily converted back to the stable alkenes. Another challenge for such transformations is the formation of unwanted by-products such as alkanes (in the case of hydroformylation) and the corresponding ethers (in the case of alkoxycarbonylation) especially under acidic conditions. As a result, and to the best of our knowledge, no general and practical catalyst has yet been developed for the alkoxycarbonylation of tetra- and tri-substituted alkenes.

On the other hand, tetra- and/or tri-substituted alkenes are interesting feedstocks and this bond motif is frequently found in natural products, pharmaceuticals and petrochemicals. Interestingly, for several industrial bulk processes, pure α-olefins are not available at an economically viable price. Therefore, mixtures are used including internal and branched olefins. As an illustrative example, so-called dibutene is a mixture of more >30 compounds including mainly octenes, methylheptenes, and dimethylhexenes. Notably, this feedstock is produced via dimerization of 1-butene and 2-butene on a 500,000-ton-per-annum scale. Due to the low reactivity of sterically hindered internal olefins, a significant amount of this feedstock cannot be further functionalized, which leads to unwanted waste. Hence, the development of improved catalyst systems for the carbonylation of tetra- and tri-substituted alkenes is an important and rewarding but highly challenging task.

A scarcely explored possibility to valorize such ‘inert' internal olefins would be a fast isomerization to more reactive alkenes. Unfortunately, despite the vast knowledge on isomerization reactions^21^, such catalyst systems, which operate under mild conditions in the presence of CO, are not known. On the basis of our long-standing interest in this area^22^,^23^ as well as the elegant works of other groups^24^,^25^,^26^,^27^,^28^, we started to explore the development of a more efficient isomerization–carbonylation catalyst. In this regard, herein we report a rationally designed palladium catalyst, which allows for alkoxycarbonylations of both highly demanding alkenes and industrially relevant bulk olefins such as ethylene with unpreceded activity and selectivity.

## Results

### Reaction concept

To develop the first general alkoxycarbonylation catalyst for ‘non-reactive' olefins, we focused on the alkoxycarbonylation of tetramethylethylene **1a** as the benchmark substrate. In our initial attempts, we performed catalytic experiments with two state-of-the-art palladium catalysts: Pd_2_(dba)_3_/**L1**/MeSO_3_H (refs 10, 11, 29) known from the methyl methacrylate process developed by Eastham *et al*. and established by Lucite International and the Shell system Pd(OAc)_2_/**L2**/MeSO_3_H pioneered by Drent and co-workers for the methoxycarbonylation of alkynes^30^,^31^,^32^,^33^,^34^,^35^. However, in both cases no carbonylation occurred and only the corresponding ether—resulting from electrophilic addition of methanol—was detected in 50 and 45% yield, respectively. Obviously, to realize the alkoxycarbonylation of **1a**, the development of a new catalyst system is imperative. According to the so-called hydride mechanism (see Supplementary Fig. 1 for details), successful alkoxycarbonylation requires the formation of a palladium hydride complex^26^,^36^,^37^,^38^,^39^.

After coordination of the alkene to this complex followed by migratory insertion into the Pd–H bond, the corresponding alkyl complex is obtained, which is transformed into an acyl complex by the migratory insertion of CO. Finally, inter- or intramolecular nucleophilic attack of methanol on the acyl carbonyl leads to the formation of the desired ester and regeneration of the palladium hydride species. Notably in the presence of acid, the overall rate-limiting step associated with the highest energetic barrier is the alcoholysis of the Pd-acyl species^26^,^38^.

**Figure 2:**
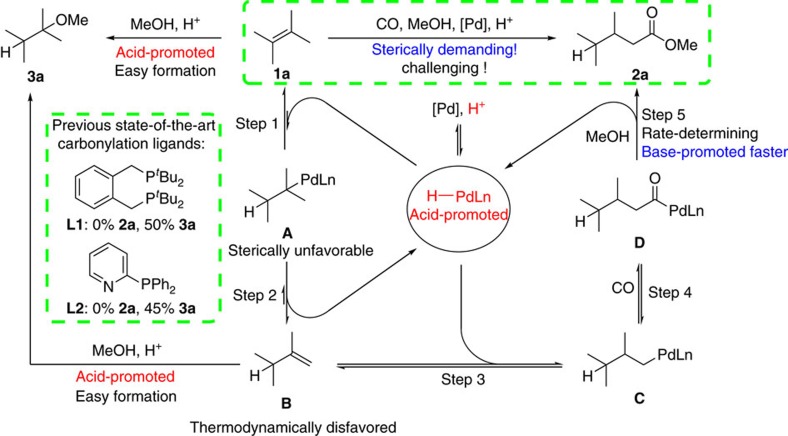
Palladium-catalysed isomerization-methoxycarbonylation of tetramethylethylene. Required steps and challenges.

**Figure 3:**
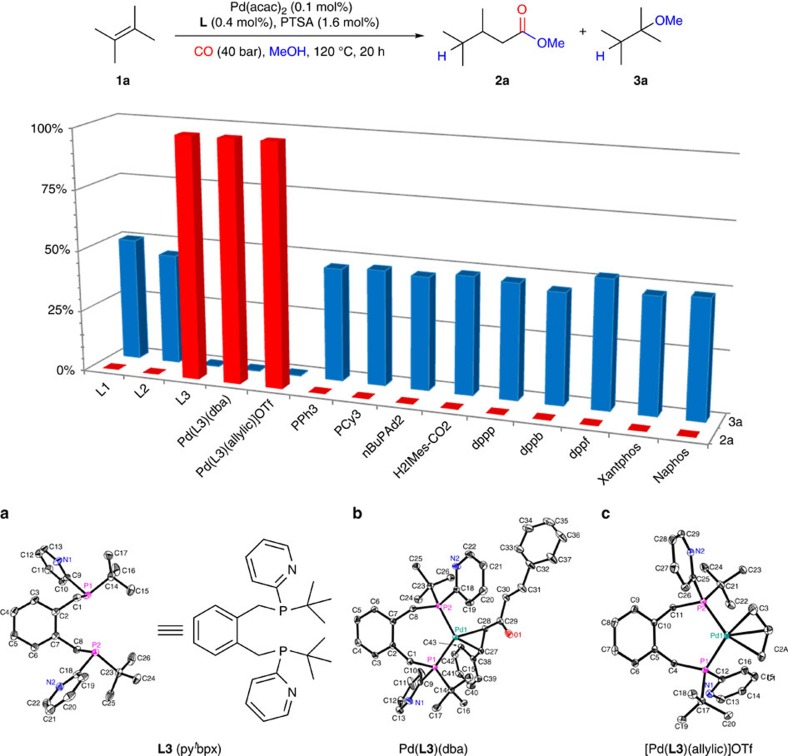
Palladium-catalysed methoxycarbonylation of tetramethylethylene 1a in the presence of various ligands. Molecular structures of **L3** and its palladium complexes: (**a**) **L3**, (**b**) Pd(**L3**)(dba), and (**c**) [Pd(**L3**)(allylic)]OTf. Hydrogen atoms are omitted for clarity. Displacement ellipsoids correspond to 30% probability. Reaction conditions: **1a** (4.0 mmol), Pd(acac)_2_ (0.1 mol%), monodentate ligand (0.8 mol%), bidentate ligand (0.4 mol%), PTSA (1.6 mol%), CO (40 bar), MeOH (2.0 ml), 120 °C, 20 h. When Pd(**L3**)(dba) or [Pd(**L3**)(allylic)]OTf was used, **L3** (0.3 mol%) was introduced in the catalytic system. The conversion of **1a** and the yields of **2a** and **3a** were determined by GC chromatography using *iso*-octane as the internal standard. acac, acetylacetonate; dba, dibenzylideneacetone; OTf, trifluoromethanesulfonate.

As shown in Fig. 2, two key problems have to be solved to realize the desired alkoxycarbonylation of tetramethylethylene **1a**: (1) the isomerization of the internal alkene to the more reactive intermediate **B** has to be enhanced under carbonylation conditions; (2) besides the irreversible alcoholysis of the Pd-acyl species, all other steps in the catalytic cycle are reversible. Hence, to shift the equilibrium towards the desired product and to avoid formation of **3a**, the alcoholysis of the final Pd-acyl species has to be accelerated markedly (step 5). This key step is known to be catalysed by base, which unfortunately impedes steps 1 and 3. To solve this contradiction, we envisioned the inclusion of an amphoteric group as part of the catalyst systems. With this idea in mind, the novel bidentate phosphine ligand **L3** containing both sterically hindered and amphoteric groups on the P-atom was designed and synthesized (see Supplementary Figs 2–4 for details). Notably, the pyridine group in this ligand should act as a proton-shuttle for the alcohol, which facilitates the alcoholysis of the Pd-acyl species^30^,^31^,^32^,^33^,^34^,^35^. Structures of **L3** and the corresponding palladium complexes were confirmed unambiguously by X-ray diffraction (Fig. 3). Both complexes Pd(**L3**)(dba) and Pd(**L3**)(allylic)OTf contain only one ligand coordinated to palladium through the P atoms. The coordination geometry at the palladium atom can be best described as trigonal-planar for Pd(**L3**)(dba) and pseudo-square-planar for Pd(**L3**)(allylic)OTf (see Supplementary Figs 5–7 for details). The ^31^P NMR spectra of these complexes in CD_2_Cl_2_ solution also indicate the clean formation of a single mono-ligated species.

To prove our concept, the methoxycarbonylation of tetramethylethylene **1a** was performed in the presence of Pd(**L3**)(dba) and Pd(**L3**)(allylic)OTf with *p*-toluenesulfonic acid monohydrate (PTSA) as co-catalyst under typical carbonylation conditions (0.1 mol% Pd catalyst, 40 bar CO, 120 °C). Indeed, the desired product **2a** was afforded in almost quantitative yield. Similar results were obtained using the *in situ-*generated catalyst (Pd(acac)_2_/**L3**/PTSA=0.1/0.4/1.6 mol%), which demonstrates the superiority of this novel ligand compared with previously privileged ligands. To also compare the reactivity of other well-known ligands such as PPh_3_, ^*n*^BuPAd_2_, dppb, dppf, Xantphos, and Naphos with our system, we investigated their effect in this challenging benchmark reaction (see Supplementary Fig. 8 for details). As shown in Fig. 3, none of the investigated monodentate and bidentate ligands provided any desired product (again only the corresponding ether by-product was obtained).

To improve the novel catalyst system further on, the effects of other critical reaction parameters such as Pd precursor, acid co-catalyst and CO pressure were investigated for the alkoxycarbonylation of **1a** in the presence of **L3** (see Supplementary Fig. 9 for details). Compared with Pd(acac)_2_, Pd(OAc)_2_ gave slightly better results under identical conditions. PdCl_2_ showed lower activity, which is attributed due to the strong coordination of the counterion. Zero-valent precursor Pd_2_(dba)_3_ catalysed the methoxycarbonylation of **1a** with comparable rate. Acid co-catalysts with strong acidity and non-coordinating anions facilitate the carbonylation of **1a** (order of activity: TfOH>H_2_SO_4_>PTSA). The effect of CO pressure is negligible, while the reaction temperature has a noticeable influence on the rate of the methoxycarbonylation of **1a**.

### Alkoxycarbonylation of various alkenes

With an optimal catalyst in hand, we investigated the scope and limitations for this system. Initially, alkoxycarbonylations of **1a** with different alcohols were carried out. To our delight, primary as well as secondary alcohols such as ethanol, tetrahydrofurfuryl alcohol, and *iso*-propanol worked well and afforded the desired esters **4**–**6** in almost quantitative yield (see Supplementary Fig. 10 for details).

**Figure 4:**
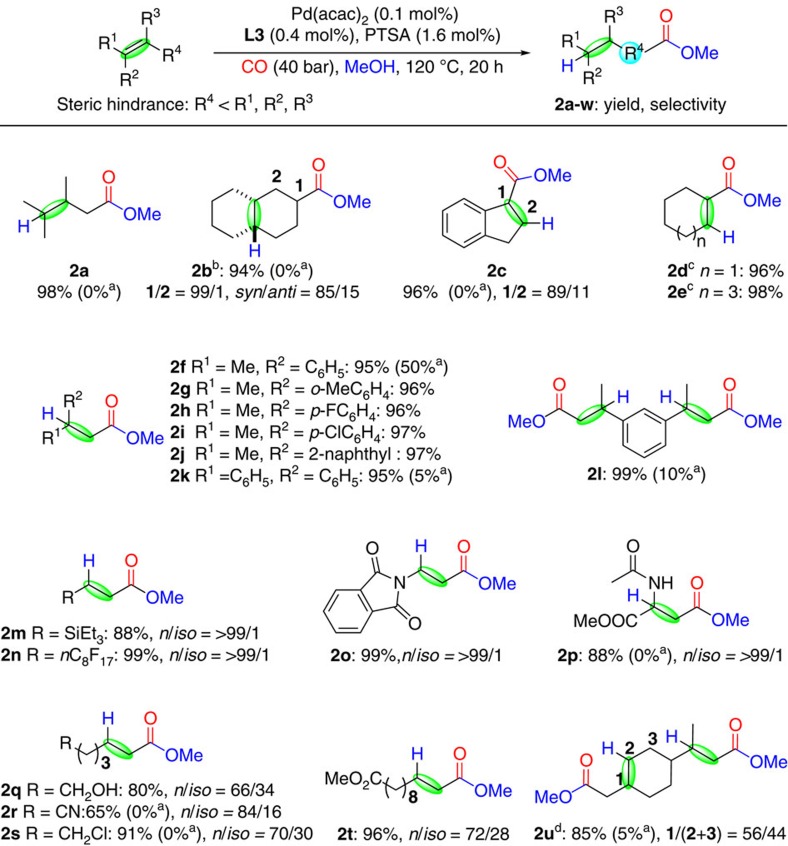
Methoxycarbonylation of various alkenes. ^a^d^*t*^bpx **L1** was used instead of py^*t*^bpx **L3**. ^b^0.5/2/8 mol% Pd(acac)_2_/**L3**/PTSA. ^c^0.04/0.16/0.64 mol% Pd(acac)_2_/**L3**/PTSA. ^d^1/4/16 mol% Pd(acac)_2_/**L3**/PTSA. Isolated yields for all products.

Next, various aliphatic and aromatic alkenes including internal and terminal ones were employed under the methoxycarbonylation conditions and afforded the desired esters in good to excellent yields (Fig. 4). In addition to **1a**, tetra-substituted 9,10-octalin **1b** was converted smoothly to **2b** in high yield with excellent regioselectivity. To the best of our knowledge, this is the first example of alkoxycarbonylations of bicyclic internal olefins, which offers new valorization possibilities for such strained intermediates. Again, there is no ester observed when using **L1** instead of **L3**, thus demonstrating the striking reactivity difference between the two systems. Aromatic olefins such as indene constitute suitable substrates and **2c** was obtained in 96% yield with high regioselectivity. Simple cycloalkenes often show low reactivity under traditional alkoxycarbonylation conditions. Gratifyingly, they are methoxycarbonylated successfully into the corresponding esters **2d** and **2e** in almost quantitative yields within 2 h (see Supplementary Fig. 11 for details). Similarly, 1-methylstyrene and related derivatives as well as 1-vinylnaphthalene and 1,1-diphenylethylene were converted to the corresponding esters **2f**-**k** in almost quantitative yields. Interestingly, diester **2l**, which has potential applications in the polymer chemistry, was also able to be obtained in 99% yield and selectivity through the dimethoxycarbonylation of 1,3-diisopropenylbenzene **1l** using our catalytic system. When **L1** was used in the carbonylation of alkenes of such kind, significantly lower yields of the desired esters and significant amounts of the corresponding ethers were observed due to the stability of the corresponding carbenium ion.

With respect to organic synthesis, this catalytic system is compatible with a broad range of functional groups. Indeed, alkenes containing electron-donating (triethylsilyl **1m**) as well as electron-withdrawing substituents (perfluoroalkyl **1n**, phthalimido **1o**) in direct conjugation with the olefin led to functionalized esters in very good yield and regioselectivity. Notably, also methyl 2-acetamidoacrylate—an example of a notoriously unreactive push-pull olefin—produced the amino acid derivative **2p** in 88% yield and highly selectively. Other olefins with remote substituents, for example, hydroxyl, nitrile, chloride and ester groups, underwent methoxycarbonylation smoothly and afforded the desired products **2q**-**t** in 65–96% yields, although in some cases the regioselectivity was lower. As an example for the carbonylation of renewable olefins (terpenes), we tested limonene. In contrast to known carbonylation catalysts^40^,^41^ double methoxycarbonylation occurred preferentially to deliver the diester product **2u** in high yield.

### Methoxycarbonylation of pharmaceuticals

**Figure 5:**
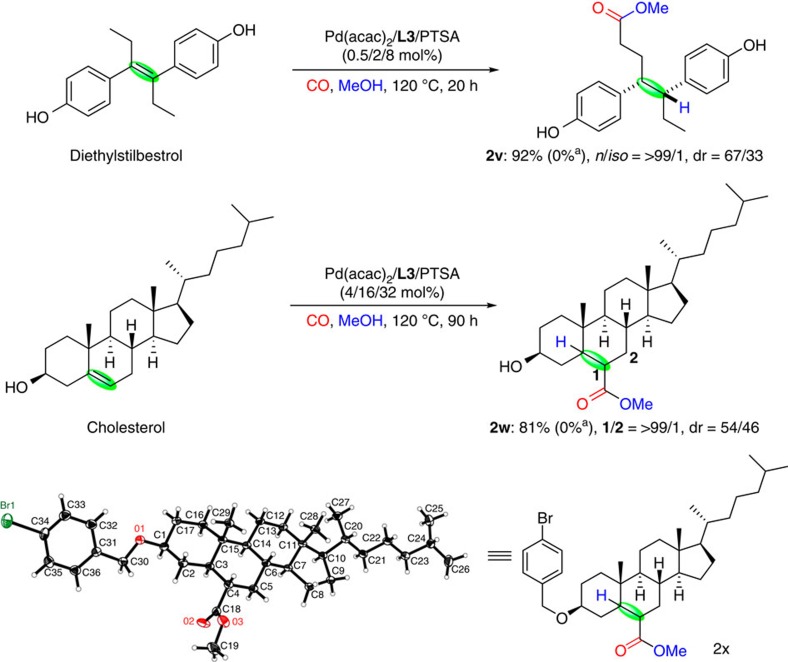
Methoxycarbonylation of pharmaceuticals. ^a^d^*t*^bpx **L1** was used instead of py^*t*^bpx **L3**. Isolated yields for all products.

For life science applications, the late-stage modification of lead compounds or even actual drugs is of current interest for the discovery of new bio-active agents. Using pharmaceuticals with inherent carbon–carbon double bonds, our alkoxycarbonylation catalyst provides an entree to otherwise not easily accessible compounds. As depicted in Fig. 5, methoxycarbonylation of diethylstilbestrol—a potent anti-tumor drug—afforded the single regioisomer **2v** in 92% yield, albeit as a mixture of diastereomers. As another example, cholesterol, which is an essential structural component of all animal cells that is required to maintain the structural integrity of membranes, is regioselectively carbonylated into the corresponding ester **2w** in 81% yield. The molecular structure of **2w** was confirmed by the X-ray diffraction of its derivative **2x**. Again, products **2v** or **2w** were not detected when using **L1** instead of **L3** under the same conditions.

### Methoxycarbonylation of industrial bulk alkenes

The ultimate goal for any new catalyst is to be superior compared with known systems in ‘real life' applications in industry. As mentioned vide supra, the methoxycarbonylation of ethylene constitutes a key step in the Lucite α-process for the preparation of methyl methacrylate.

**Figure 6:**
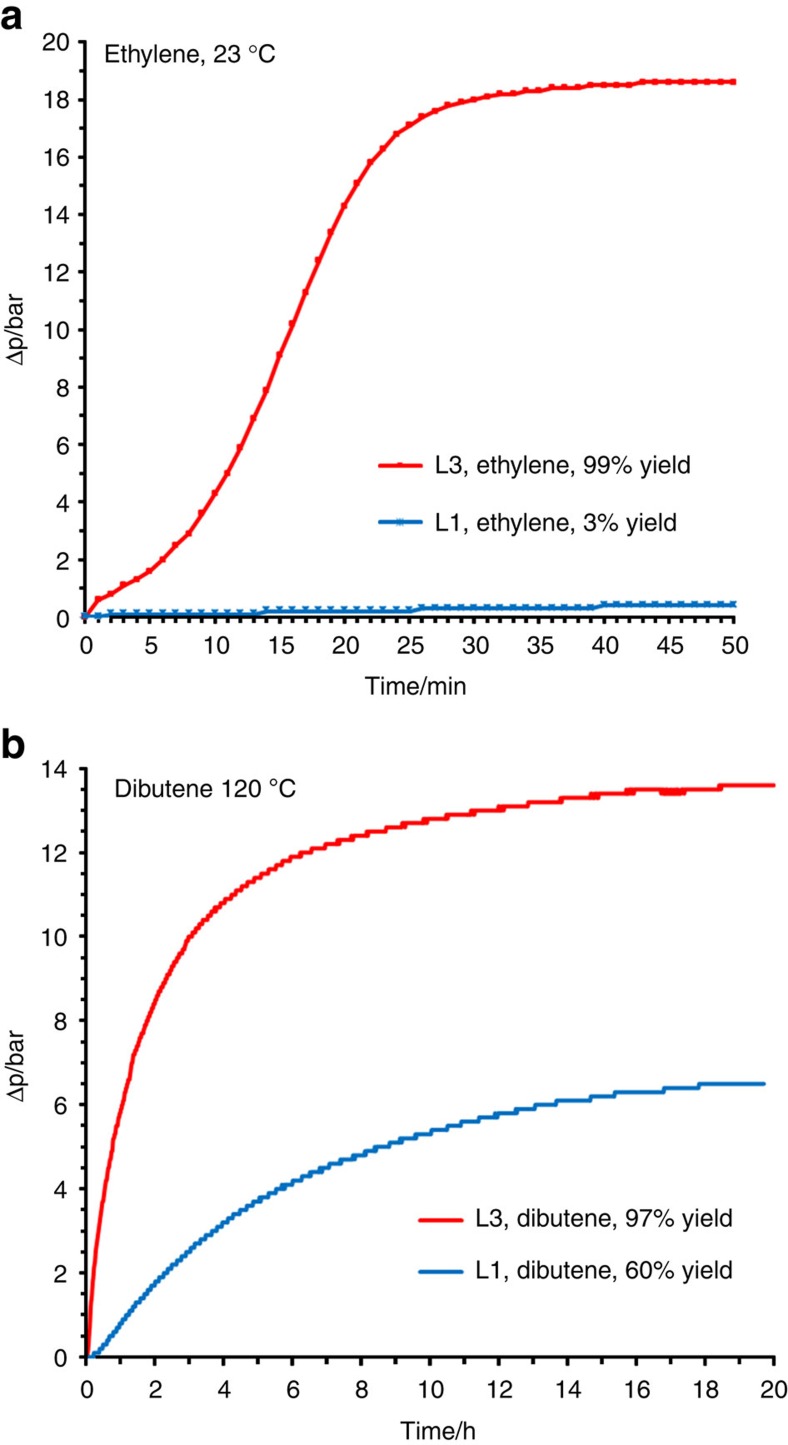
Methoxycarbonylation of industrial bulk alkenes. Ethylene at 23 °C (**a**) and dibutene at 120 °C (**b**).

As depicted in Supplementary Fig. 12, important aliphatic olefins such as ethylene and propylene were completely converted to the corresponding methyl esters in quantitative yields in the presence of Pd/**L3**/PTSA (0.04/0.16/0.6 mol%) at 80 °C within 10 min, respectively. Under such technical conditions, the new system again proved to be superior compared to the present state-of-the art industrial catalyst for such reactions. To demonstrate the striking difference in activity with the existing catalyst, ethylene was also methoxycarbonylated at room temperature. Using the commercial ligand **L1** did not result in any catalyst activity, while **L3** afforded the desired product within 30 min with excellent selectivity (>99%) (Fig. 6a). To the best of our knowledge, this is also the first time that the methoxycarbonylation of ethylene proceeded at room temperature with significant rate. Remarkably, the catalyst loading can be decreased as low as 0.6 p.p.m. for ethylene methoxycarbonylation and the desired product was afforded with unprecedented activity and chemoselectivity (TON: >1,425,000; TOF: 44,000 h^−1^; total yield: 85%, selectivity: >99%; Supplementary Methods).

As mentioned in the Introduction, another important industrial application is the alkoxycarbonylation of the feedstock dibutene. In contrast to ethylene, the key issue for this feedstock is to achieve full conversion, which is not yet possible due to the low reactivity of the inherent tetra-substituted olefins. To our delight, the new catalyst system allowed complete use of the substrate and the corresponding ester products were obtained in 97% yield (Fig. 6b). Again, this yield is unprecedented and such high conversion was not reported with any previously available catalyst systems.

In conclusion, we have developed a palladium catalyst based on the novel ligand **L3** (py^*t*^bpx) for the general alkoxycarbonylation of olefins. With respect to reactivity, this catalyst clearly surpasses any known Reppe carbonylation catalyst in all applications studied so far. In addition to interesting synthetic examples, demanding challenging and industrially important bulk alkenes can be alkoxycarbonylated with unprecedented activity.

## Methods

Experimental procedures are described in Supplementary Methods in detail.

### Data availability

Crystal structures have been deposited at the Cambridge Crystallographic Data Centre and allocated the deposition numbers CCDC 1483958 (**L3**), 1483956 ([Pd(**L3**)(dba)]), CCDC 1483957 ([Pd(**L3**)(allylic)]OTf), and 1483955 (**2x**). Crystal data are also provided in Supplementary Tables 1–4. Spectra of products can be found in Supplementary Fig. 13. All other data are available from the authors upon reasonable request.

## Additional information

**How to cite this article:** Dong, K. *et al*. Highly active and efficient catalysts for alkoxycarbonylation of alkenes. *Nat. Commun.* **8,** 14117 doi: 10.1038/ncomms14117 (2017).

**Publisher's note:** Springer Nature remains neutral with regard to jurisdictional claims in published maps and institutional affiliations.

## Supplementary Material

Supplementary Information Supplementary figures, supplementary tables, supplementary methods and supplementary references.

Peer review file

## Figures and Tables

**Figure 1:**
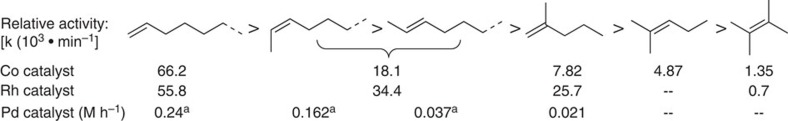
Reaction rates of alkene carbonylations. ^a^Heptenes were used.
