# Supplementary material for: Highly active and efficient catalysts for alkoxycarbonylation of alkenes
Source: Nat Commun. 2017 Jan 25;8:14117. doi: 10.1038/ncomms14117 (PMC5288498; doi:10.1038/ncomms14117)
Supplement: Supplementary Information — Supplementary figures, supplementary tables, supplementary methods and supplementary references. [file ncomms14117-s1.pdf]

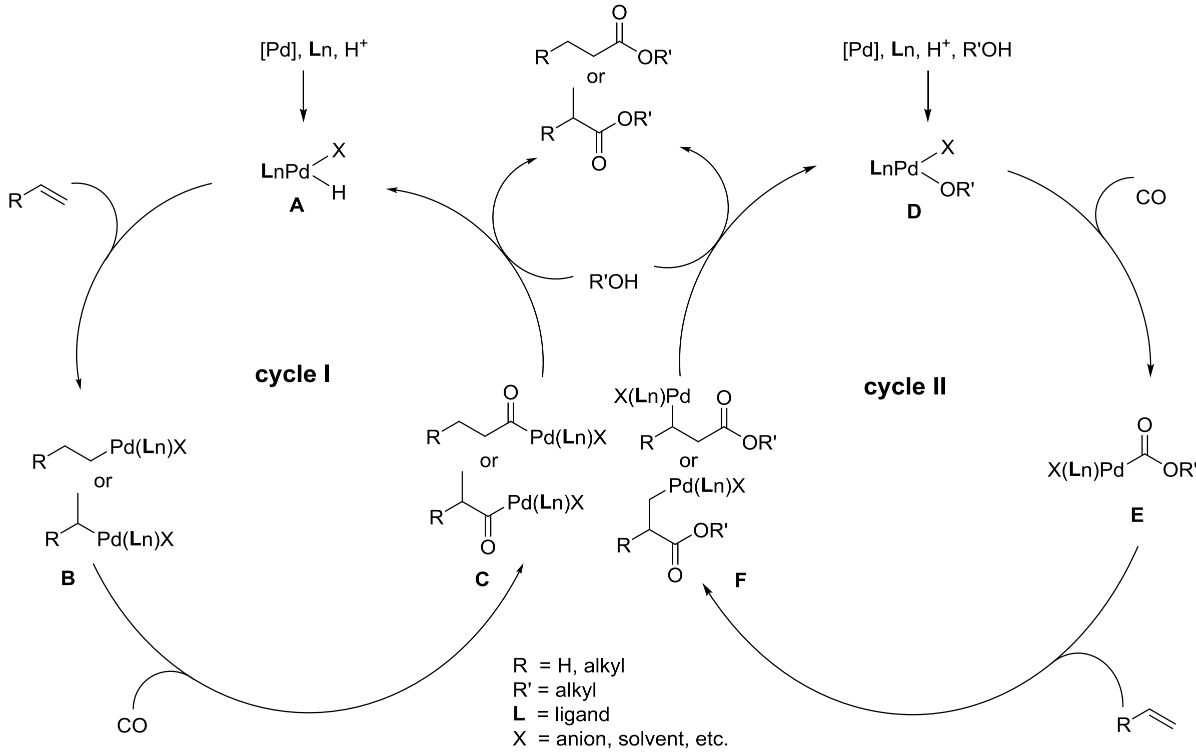

Supplementary Figure 1. The mechanistic pathway for palladium-catalyzed alkoxycarbonylation of alkenes (e.g. terminal olefins). There is a lot of evidence and general agreement that systems affording ester product operate exclusively by the hydride catalytic **cycle I** and the alternative **cycle II** was provided in the copolymerization of alkenes with CO and carbonylation of alkynes as well. **Cycle I** (hydride mechanism): this pathway has been proposed with the formation of a palladium hydride complex **A**. Coordination of the alkene, followed by migratory insertion into the Pd-H bond then affords a Pd–alkyl complex **B**, which is transformed into an acyl complex **C** by the migratory insertion of CO. Inter- or intramolecular nucleophilic attack of methanol on the acyl carbonyl leads to the formation of the desired ester and regeneration of the palladium hydride species. **Cycle II** (carboalkoxy mechanism): this pathway starts with the generation of the Pd-OR' species **D** followed by the coordination and insertion of CO. After migratory insertion of alkenes into the Pd-COOR' complex **E**, the alkyl palladium **F** was formed. Final protonation of the alkyl complex **F** with alcohol affords the desired product and the methoxy-palladium species.

Supplementary Methods

General information

Air- and moisture-sensitive syntheses were performed under argon atmosphere. Chemicals were purchased from Aldrich, TCI, Alfa, Fluka, Acros, or Strem. Unless otherwise noted, all commercial reagents were used without further purification.

Products were characterized by ^{1}H NMR, ^{13}C NMR, and HRMS spectroscopy. ^{1}H and ^{13}C NMR spectra were recorded on Bruker Avance 300 (300 MHz) or 400 (400M) NMR spectrometers. Chemical shifts δ (ppm) are given relative to solvent: references for $CDCl_3$ were 7.26 ppm (^{1}H-NMR) and 77.16 ppm (^{13}C-NMR), for CD_2Cl_2 were 3.76 ppm (^{1}H-NMR) and 54.2 ppm (^{13}C-NMR), and for C_6D_6 were 7.12 ppm (^{1}H-NMR) and 116.0 ppm (^{13}C-NMR). ^{13}C-NMR spectra were acquired on a broad band decoupled mode. Multiplets were assigned as s (singlet), d (doublet), t (triplet), dd (doublet of doublet), dt (doublet of triplet), td (triplet of doublet), and m (multiplet).

EI (Electron impact) mass spectra were recorded on an MAT 95XP spectrometer (70 eV, Thermo ELECTRON CORPORATION). ESI (electrospray ionization) high resolution mass spectra were recorded on an Agilent Technologies 6210 TOF LC/MS using H_2O + 0.1% formic acid (10%) and methanol (90%) as eluent. GC analysis was performed on a Agilent 7890A chromatograph with a a 29 m HP5 column.

Data were collected on a Bruker Kappa APEX II Duo diffractometer. The structures were solved by direct methods (SHELXS-97: Sheldrick, G. M. *Acta Crystallogr.* **2008**, *A64*, 112.) and refined by full-matrix least-squares procedures on F^2 (SHELXL-2014: G. M. Sheldrick, *Acta Crystallogr.* **2015**, *C71*, 3.). XP (Bruker AXS) was used for graphical representations.

Alkoxycarbonylation products were isolated from the reaction mixture by distillation directly or column chromatography on silica gel after solvent evaporation (eluent: heptane/ethyl acetate = 20/1–1/1).

Synthesis and characterization of ligand L3

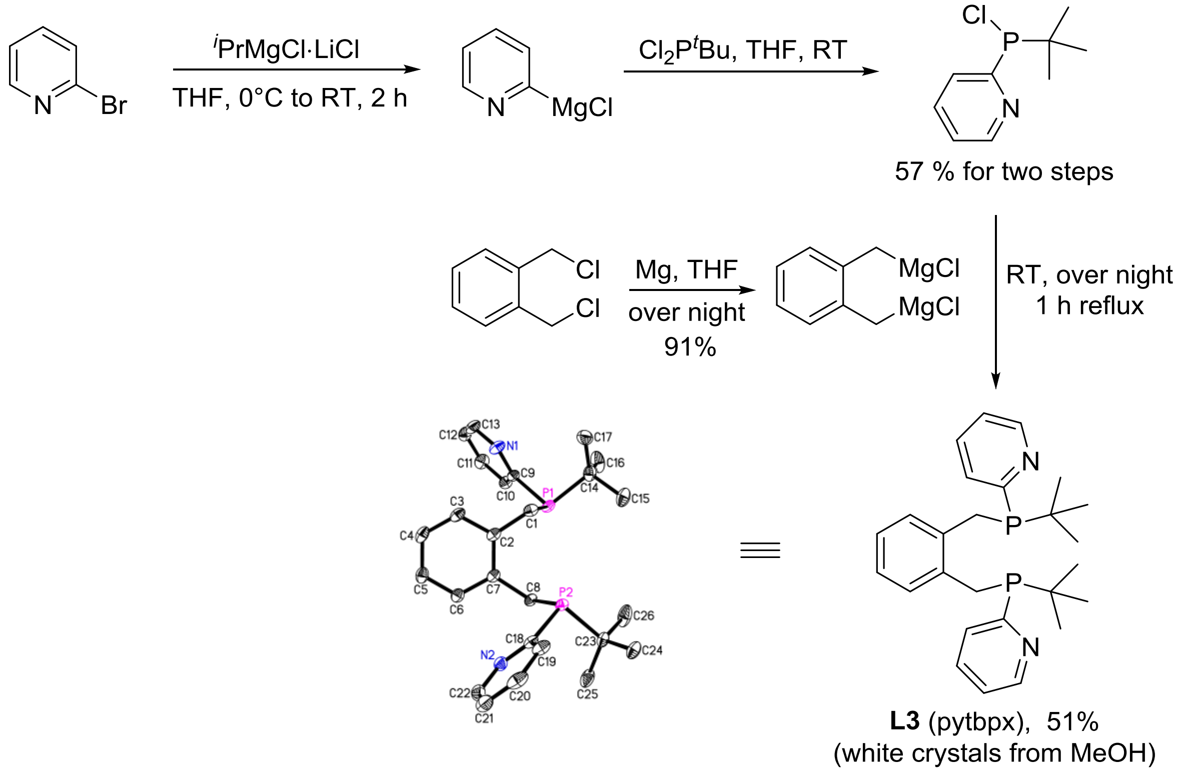

Supplementary Figure 2. Synthesis and characterization of ligand L3 (py^{t}bpx)

Synthesis of 2-(*tert*-butylchlorophosphanyl)pyridine: 2-bromopyridine (953.5 µL, 10 mmol) was dropped into an isopropylmagnesium chloride solution (8.07 mL, 1.3 M in THF, 10.5 mmol) under argon atmosphere at -15 ° C. The solution immediately turned yellow. The reaction solution was allowed to room temperature and continued to stir for 1 h.

The fresh Grignard reagent (pyridin-2-ylmagnesium chloride) was added dropwise into the solution of *tert*-butyldichlorophosphane (1.748 g, 11 mmol in 10 mL THF) at -15 ^{o}C. The reaction mixture was initially a clear yellow solution, and then became cloudy. The reaction was allowed to warm to room temperature and stirred overnight. After removing the solvent in a high vacuum, a white solid containing the brown spots was obtained. The solid was suspended in 20 ml of heptane and crushed in an ultrasonic bath. After standing for 0.5 h the solution was decanted. The process was repeated 2 times each with 10-20 ml of heptane. Then combining the heptane solution and the desired product was afforded as colorless oil by distillation (1.08 g, 50% yield).

^{1}H NMR (300 MHz, C_6D_6): δ 8.36 (m, 1H, Py), 7.67 (m, 1H, Py), 7.03-6.93 (m, 1H, Py), 6.55-6.46 (m, 1H, Py), 1.07 (d, J = 13.3 Hz, 9H, t-Bu) ^{13}C NMR (75 MHz, C_6D_6): δ 162.9, 162.6, 148.8, 135.5, 125.8, 125.7, 122.8, 35.3, 34.8, 25.9 and 25.8. ^{31}P NMR (121 MHz,

C_6D_6) δ 97.9. MS (EI) *m*:*z* (relative intensity): 201 (M^+,2), 147(32), 145 (100), 109 (17), 78 (8), 57.1 (17).

Synthesis of the ligand L3 (py^{t}bpx): Mg powder (675 mg, 27.8 mmol, 4 eq) was heated at 90 ° C for 45 minutes. After cooling to room temperature 2 grains of iodine were added and dissolved in 20 ml of THF. The suspension was stirred for 10 minutes until the yellow color of iodine disappeared. Then the THF solution was decanted and the activated magnesium powder was washed 2 times with 1-2 ml of THF. After fresh THF (20 mL) was added again a solution of α,α'-dichloro-*o*-xylene (1.21 g, 6.9 mmol in 70 ml THF) was slowly dropped with the syringe pump at room temperature. The THF mixture turned slowly dark and stirred overnight. The unreacted magnesium powder was filtered off from the reaction mixture. Quantitative determination of the content of the Grignard solution: 1 mL Grignard solution was quenched with 2 mL of 0.1 M HCl and the excess acid was titrated with 0.1 M NaOH. Bromocresol green (0.04% in water) was used as an indicator (color change is from yellow to blue).The present Grignard solution was determined as 0.063M. That's over 90% yield.
2-(tert-butylchlorophosphanyl)pyridine solution (1.8 g, 8.66 mmol in 10 mL THF) was cooled to -60 ° C under argon. Then the fresh Grignard solution (55 mL, 0.063 M, 3.46 mmol) was slowly added dropwise at this temperature with the syringe pump. The mixture was allowed to room temperature and stirred overnight which gave a clear yellow solution. To complete the reaction the solution was heated under reflux for 1 hour. After removal of THF in vacuum a light yellow solid was provided. 10 mL of water and 10 ml of ether were added into the solid and two separated clear phases observed. The aqueous phase was extracted with ether (10 mL x 2). After the combined organic phase was dried with anhydrous Na_2SO_4, the ether was removed under high vacuum and provided an almost colorless solid. The solid was dissolved in 5 mL MeOH under heating on a water bath and filtered through celite. After cooling at -28 ^oC overnight the desired ligand was afforded as white crystals (772 mg, 51%).
^1H NMR (300 MHz, C_6D_6): δ 8.58 (m, 2H, Py), 7.31-7.30 (m, 2H, benzene), 7.30-7.22 (m, 2H, Py), 6.85-6.77 (m, 2H, Py), 6.73 (m, 2H, benzene), 6.57-6.50 (m, 2H, py), 4.33(dd, J = 13.3, 4.3 Hz, 2H, CH_2), 3.72-3.62 (m, 2H, CH_2), 1.21(d, *J* = 11.8 Hz, 18H, ^{t}Bu). ^{13}C NMR (75 MHz, C_6D_6): δ 161.3, 161.1, 149.6, 137.8, 137.7, 134.5, 133.3, 132.7, 131.4, 131.3, 125.7, 122.9, 30.7, 30.5, 28.2, 28.0, 26.5, 26.4, 26.2, 26.1.· ^{31}P NMR (121 MHz, C_6D_6) δ 8.8. EA calcd for $C_{26}H_{34}N_2P_2$: C, 71.54; H, 7.85; N, 6.56; P,14.35. Found: C, 71.21; H, 7.55; N, 6.56; P, 14.35.

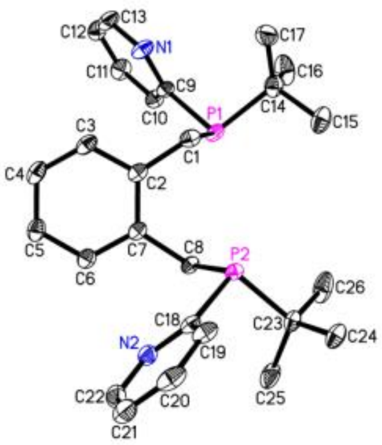

Supplementary Figure 3. **Molecular structure of the ligand L3 (py^{t}bpx).** Hydrogen atoms have been omitted for clarity. Displacement ellipsoids correspond to 30% probability. Selected bond lengths [Å]: P1-C1, 1.8548(13); P1-C9, 1.8417(13); P1-C14, 1.8835(17); P2-C8, 1.8507(12); P2-C18, 1.8399(13); P2-C23, 1.8862(14).

Supplementary Table 1. Crystal data for the ligand L3 (py^{t}bpx): Single crystals of the ligand **L3** were obtained from the recrystallization in methanol.

Empirical formula	$C_{26}H_{34}N_2P_2$
Formula weight	436.49
Temperature	150(2) K
Wavelength	0.71073 Å
Crystal system	triclinic
Space group	*P*-1
Unit cell dimensions	a = 7.9694(2) Å α = 75.2895(6)°
	b = 16.5043(4) Å β = 84.0696(7)°
	c = 19.6929(5) Å γ = 86.1823(7)°
Volume	2489.69(11) Å^3
Z	4
Density (calculated)	1.164 Mg/m^3
Absorption coefficient	0.189 mm^{-1}
F(000)	936
Crystal size	0.517 x 0.479 x 0.420 mm
Theta range for data collection	1.861 to 27.500°.
Index ranges	-10<=h<=10, -21<=k<=21, -25<=l<=25
Reflections collected	86798
Independent reflections	11448 [R(int) = 0.0210]
Completeness to theta = 25.242°	99.9 %
Max. and min. transmission	0.93 and 0.90

Data / restraints / parameters	11448 / 9 / 552
Goodness-of-fit on F^2	1.040
Final R indices [I>2σ(I)]	R1 = 0.0335, wR2 = 0.0872
R indices (all data)	R1 = 0.0392, wR2 = 0.0933
Largest diff. peak and hole	0.435 and -0.262 e·Å^{-3}
CCDC no.	CCDC1483958

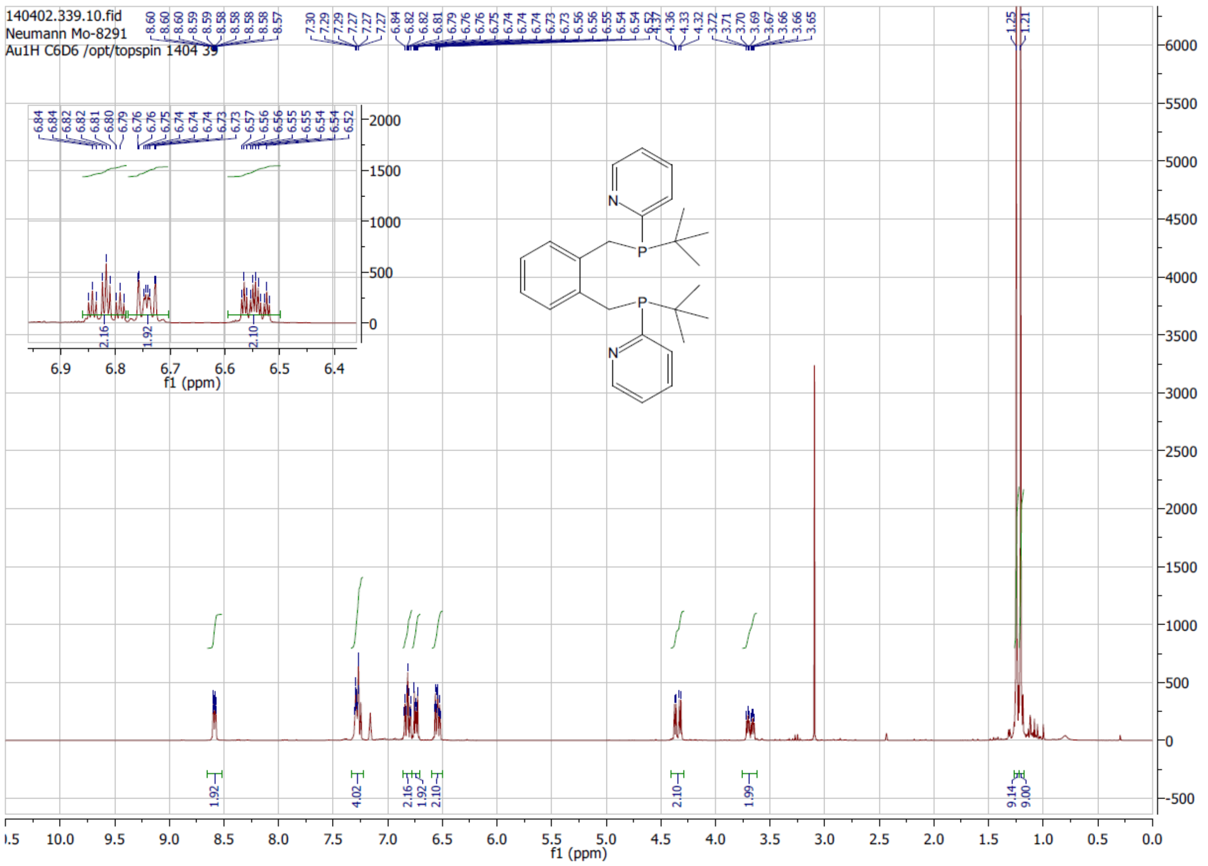

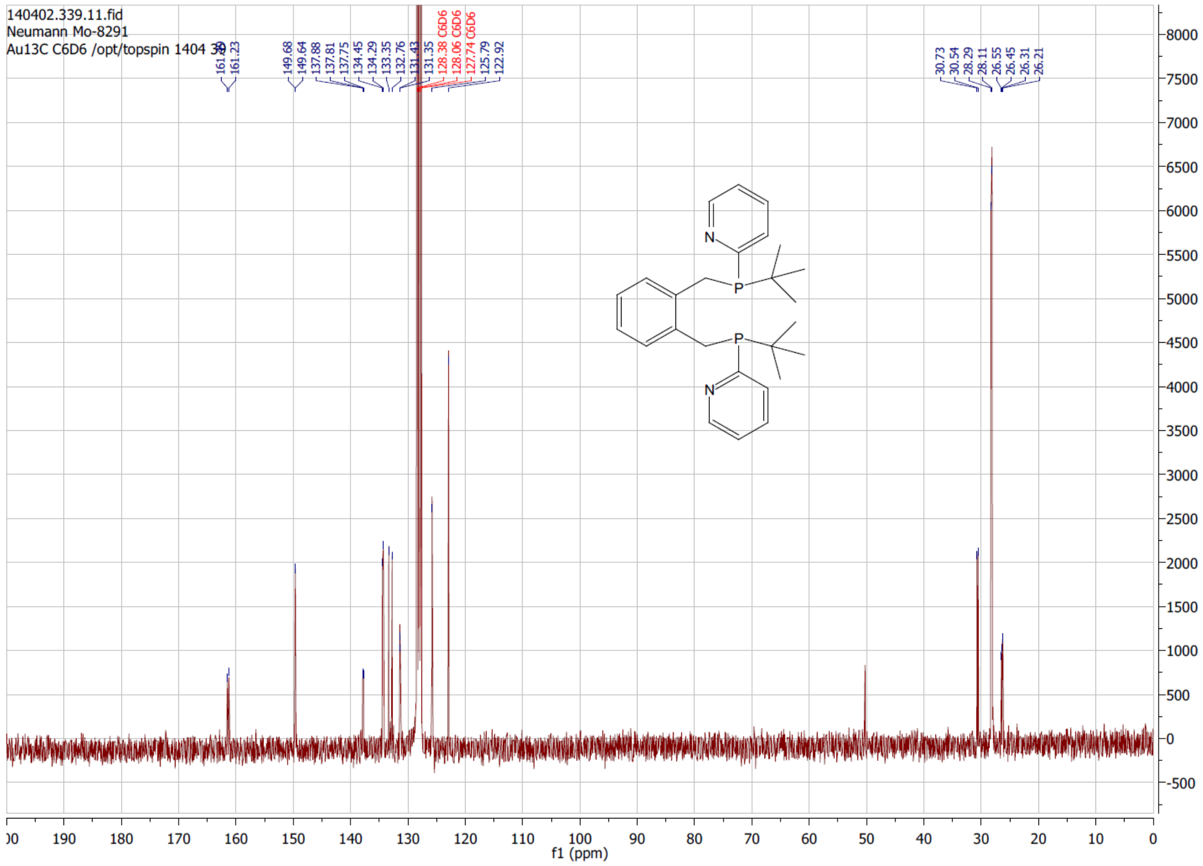

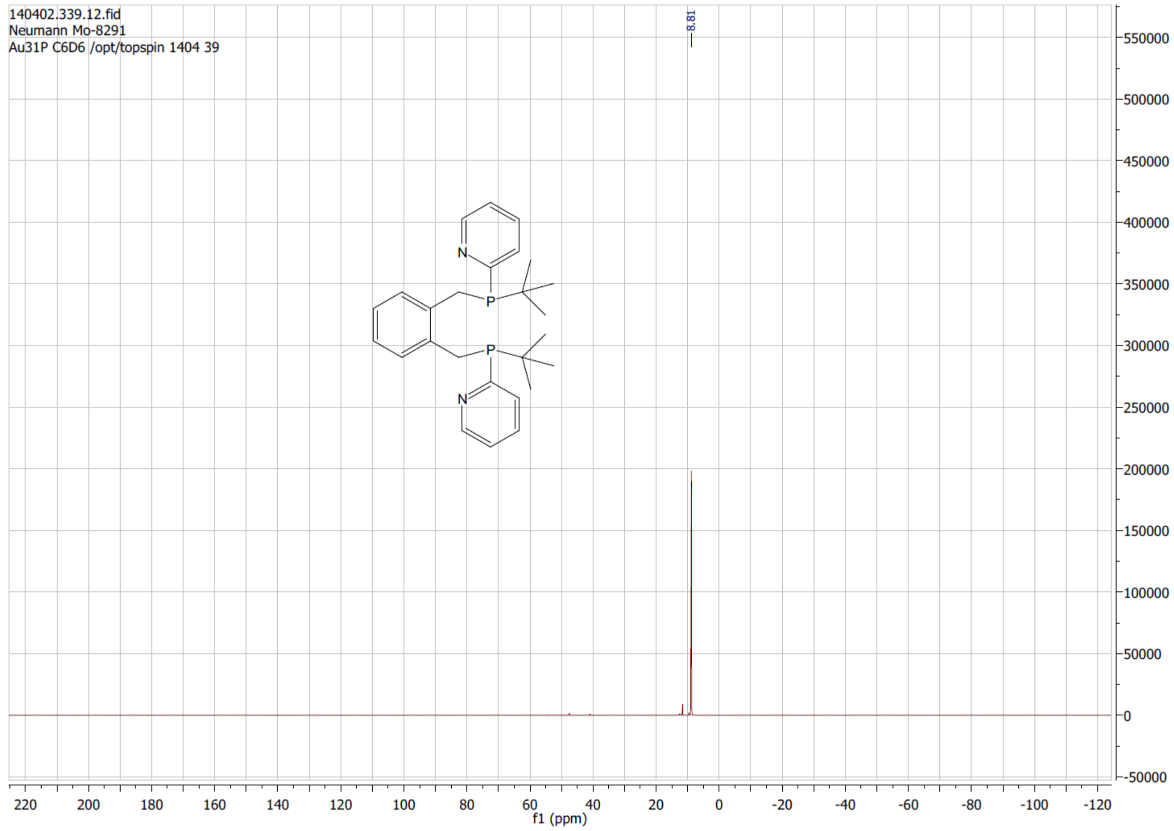

Supplementary Figure 4. ^{1}H, ^{13}C and ^{31}P NMR spectra of the ligand L3.

X-ray crystal structure analysis of the catalyst precursors Pd(L3)(dba) and Pd[(L3)(allyl)]OTf.

Single crystals of the catalyst precursor **Pd(L3)(dba)** was obtained from recrystallization in acetone/pentane at 0 °C. Crystal data are given in **Table S2.**

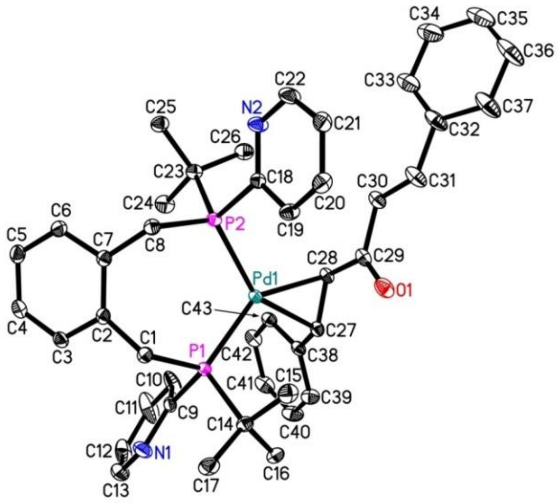

Supplementary Figure 5. Molecular structure of complex Pd(**L3**)(dba). Hydrogen atoms have been omitted for clarity. Displacement ellipsoids correspond to 30% probability. Selected bond lengths [Å] and angles [°]: Pd1-P1, 2.3002(4); Pd1-P2, 2.3202(4); Pd1-C27, 2.1633(15); Pd1-C28, 2.1527(15); P1-C1, 1.8422(15); P1-C9, 1.8365(16); P1-C14, 1.8772(16); P2-C8, 1.8643(16); P2-C18, 1.8436(15); P2-C23, 1.8784(16); P1-Pd1-P2, 105.516(14).

Supplementary Table 2. Crystal data for Pd(L3)(dba).

Empirical formula	$C_{43}H_{48}N_2OP_2Pd$
Formula weight	777.17
Temperature	150(2) K
Wavelength	0.71073 Å
Crystal system	triclinic
Space group	*P*-1
Unit cell dimensions	a = 9.2677(2) Å α = 76.3232(6)°
	b = 11.0597(2) Å β = 89.1885(6)°
	c = 19.5326(4) Å γ = 80.8475(6)°
Volume	1919.94(7) Å^3
Z	2
Density (calculated)	1.344 Mg/m^3
Absorption coefficient	0.602 mm^{-1}

F(000)	808
Crystal size	0.261 x 0.253 x 0.234 mm
Theta range for data collection	1.920 to 28.000°.
Index ranges	-12<=h<=12, -14<=k<=14, -25<=l<=25
Reflections collected	38144
Independent reflections	9298 [R(int) = 0.0214]
Completeness to theta = 25.242°	100.0 %
Max. and min. transmission	0.87 and 0.80
Data / restraints / parameters	9298 / 0 / 456
Goodness-of-fit on F^2	1.030
Final R indices [$I>2\sigma(I)$]	R1 = 0.0238, wR2 = 0.0571
R indices (all data)	R1 = 0.0290, wR2 = 0.0600
Largest diff. peak and hole	0.607 and -0.264 e·$Å^{-3}$
CCDC no.	CCDC1483956

Single crystals of the catalyst precursor **Pd[(L3)(allyl)]OTf** were obtained from recrystallization in dichloromethane//pentane at 0 °C. Crystal data are given in **Table S3.**

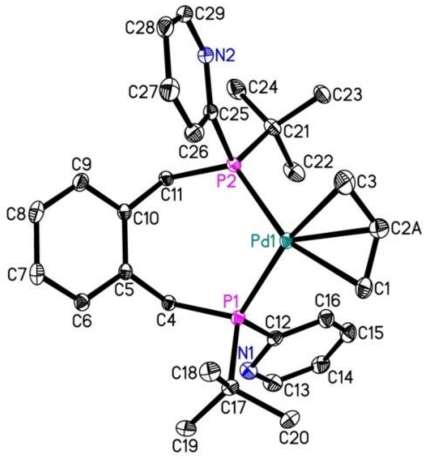

Supplementary Figure 6. Molecular structure of complex Pd[(**L3**)(allyl)]OTf. Hydrogen atoms and the OTf^- anion have been omitted for clarity. Displacement ellipsoids correspond to 30% probability. Selected bond lengths [Å] and angles [°]: Pd1-P1, 2.3292(6); Pd1-P2, 2.3084(6); Pd1-C1, 2.200(2); Pd1-C2A, 2.193(3); Pd1-C3, 2.186(3); P1-C4, 1.839(2); P1-C12, 1.837(2); P1-C17, 1.881(2); P2-C11, 1.837(2); P2-C21, 1.867(2); P2-C25, 1.835(2); P1-Pd1-P2, 101.81(2).

Supplementary Table 3. Crystal data for Pd[(L3)(allyl)]OTf.

Empirical formula	$C_{30}H_{39}F_3N_2O_3P_2PdS$
Formula weight	733.03
Temperature	150(2) K
Wavelength	0.71073 Å
Crystal system	monoclinic
Space group	$P2_1/c$
Unit cell dimensions	a = 20.7226(3) Å α = 90°
	b = 9.45540(10) Å β = 115.8256(7)°
	c = 18.1542(3) Å γ = 90°
Volume	3201.87(8) Å^3
Z	4
Density (calculated)	1.521 Mg/m^3
Absorption coefficient	0.796 mm^{-1}
F(000)	1504
Crystal size	0.457 x 0.292 x 0.136 mm
Theta range for data collection	2.184 to 27.999°
Index ranges	-27<=h<=27, -11<=k<=12, -23<=l<=23
Reflections collected	55970
Independent reflections	7713 [R(int) = 0.0398]
Completeness to theta = 25.242°	99.9 %
Max. and min. transmission	0.90 and 0.71
Data / restraints / parameters	7713 / 9 / 385
Goodness-of-fit on F^2	1.036
Final R indices [$I>2\sigma(I)$]	R1 = 0.0334, wR2 = 0.0820
R indices (all data)	R1 = 0.0393, wR2 = 0.0872
Largest diff. peak and hole	1.565 and -0.632 e·Å^{-3}
CCDC no.	CCDC1483957

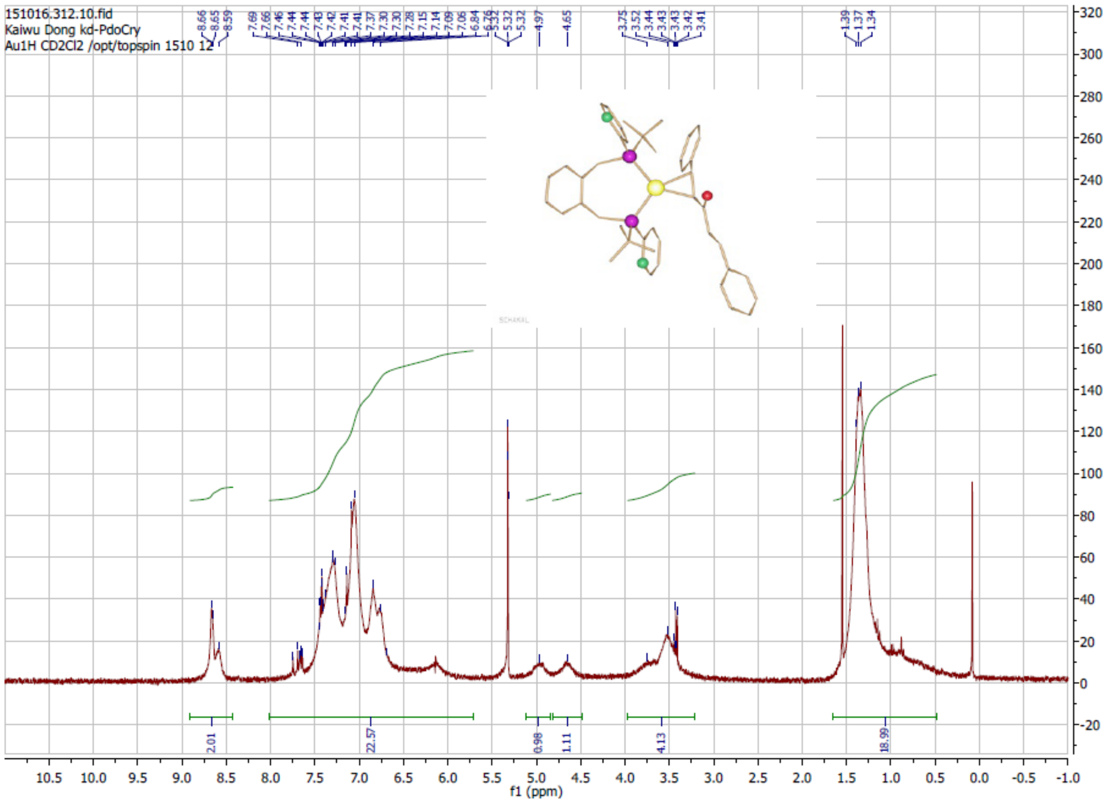
151016.312.10.fid
Kaiwu Dong kd-PdoCry
Au1H CD2Cl2 /opt/topspin 1510 12
f1 (ppm)

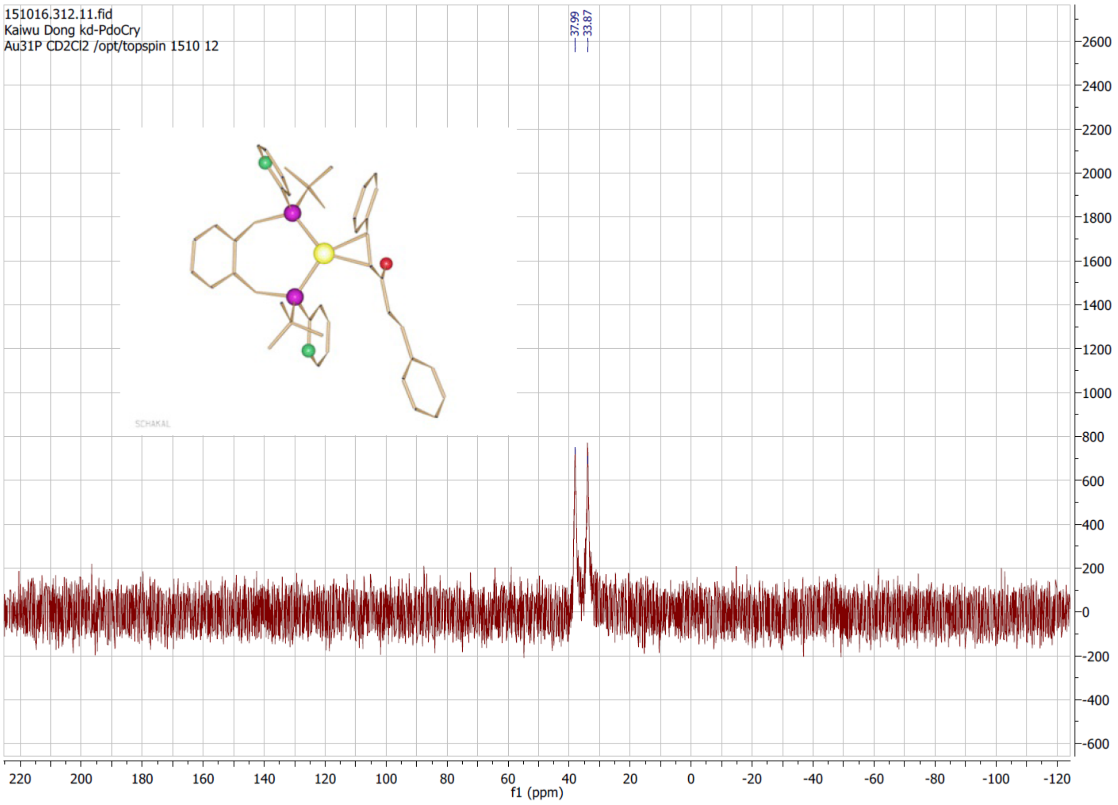
151016.312.11.fid
Kaiwu Dong kd-PdoCry
Au31P CD2Cl2 /opt/topspin 1510 12
37.99
33.87
f1 (ppm)

(**a**)

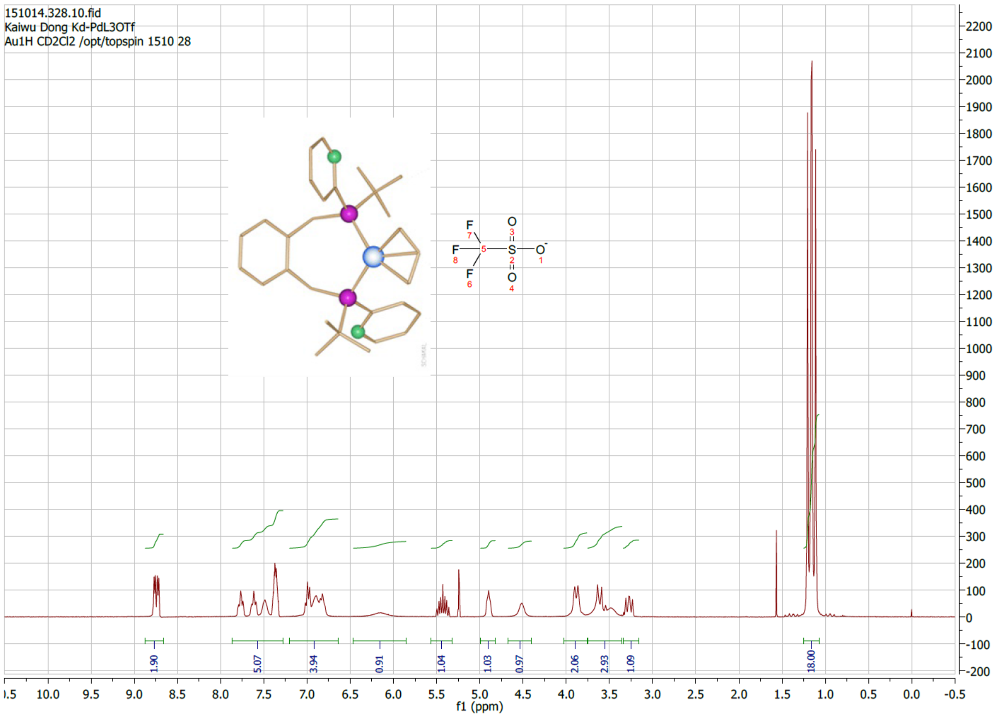

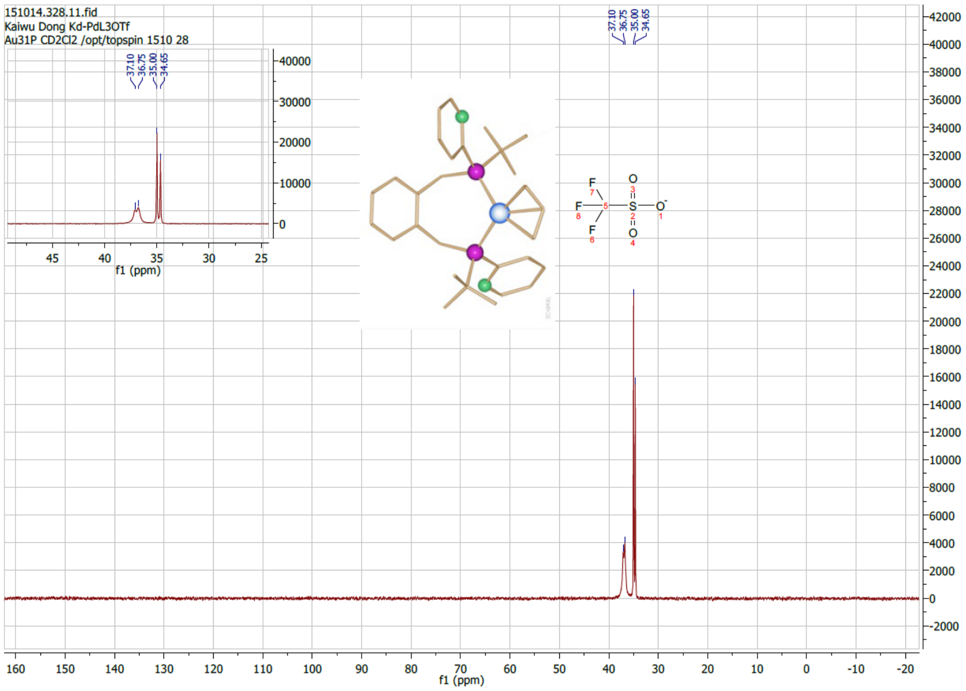

(b)

Supplementary Figure 7. ^{1}H and ^{31}P NMR spectra measured for single crystals of Pd(**L3**)(dba) (**a**) and Pd[(**L3**)(allyl)]OTf **(b)** (CD_2Cl_2, rt).

Palladium-catalyzed methoxycarbonylation of tetramethylethylene 1a in the presence of various ligands.

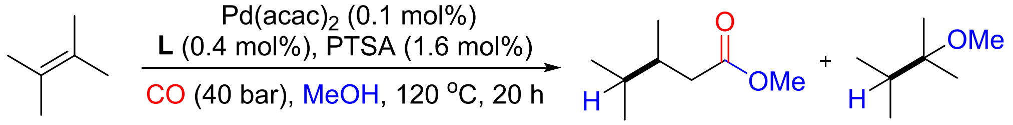

1a **2a** **3a**

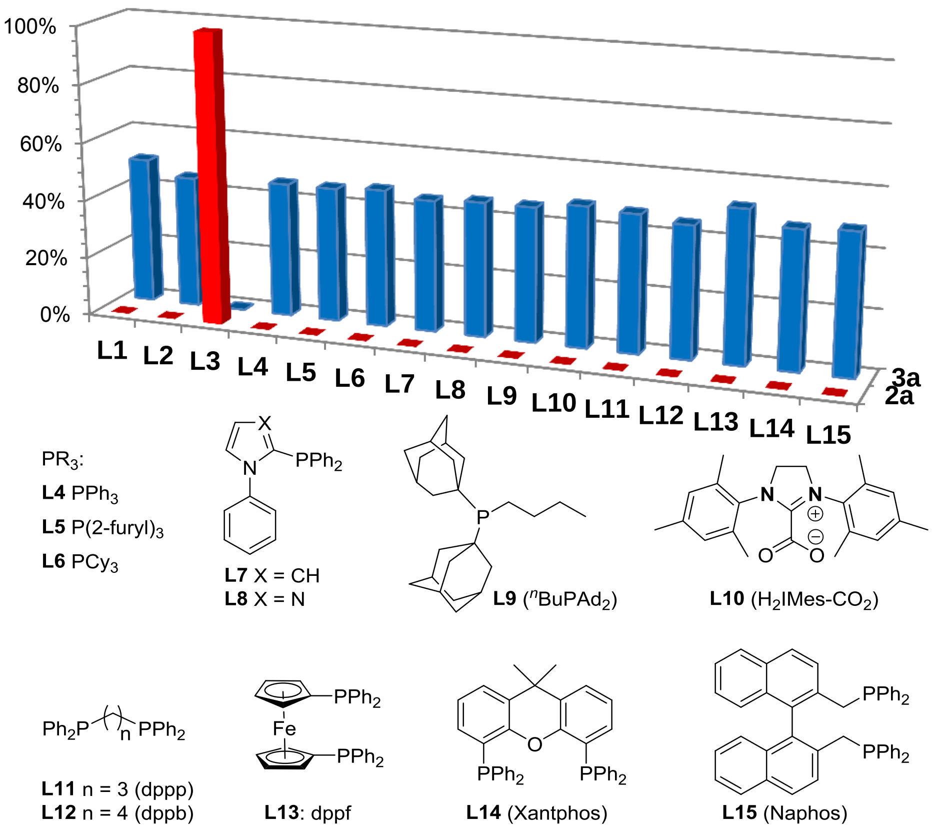

Supplementary Figure 8. Palladium-catalyzed methoxycarbonylation of tetramethylethylene 1a in the presence of various ligands. Reaction conditions: **1a** (4.0 mmol), Pd(acac)$_2$ (0.1 mol%), monodentate ligand (0.8 mol%), bidentate ligand (0.4 mol%), PTSA (1.6 mol%), CO (40 bar), MeOH (2.0 mL), 120 ^{o}C, 20 h. The conversion of **1a** and the yields of **2a** and **3a** were determined by GC analysis using isooctane as the internal standard. PTSA = *p*-toluenesulfonic acid monohydrate.

General procedure. Under argon atmosphere, a vial (4 mL) was charged with [Pd(acac)$_2$] (1.22 mg, 0.1 mol%), monodentate ligand (0.8 mol%) or bidentate ligand (0.4 mol%), PTSA (12.2 mg, 1.6 mol%) and a stirring bar. Then MeOH (2.0 mL) and **1a** (0.48 mL, 4.0 mmol)

were injected by syringe. The vial was placed in an alloyed plate, which was then transferred into an autoclave (300 mL) under argon atmosphere. The autoclave was flushed with CO three times at room temperature and then pressurized with CO to 40 bar. The reaction was performed at 120 ^{o}C for 20 h. After the reaction finished, the autoclave was cooled to room temperature and the pressure was carefully released. Isooctane (0.5 mL) was added into the reaction mixture as the internal standard and the yield was measured by GC analysis. The new ligand **L3** gave the desired product **2a** in 99% yield while the other ligands only gave the byproduct **3a** in moderate yield.

Investigating various parameters of the palladium/L3-catalyzed alkoxycarbonylation of tetramethylethylene 1a.

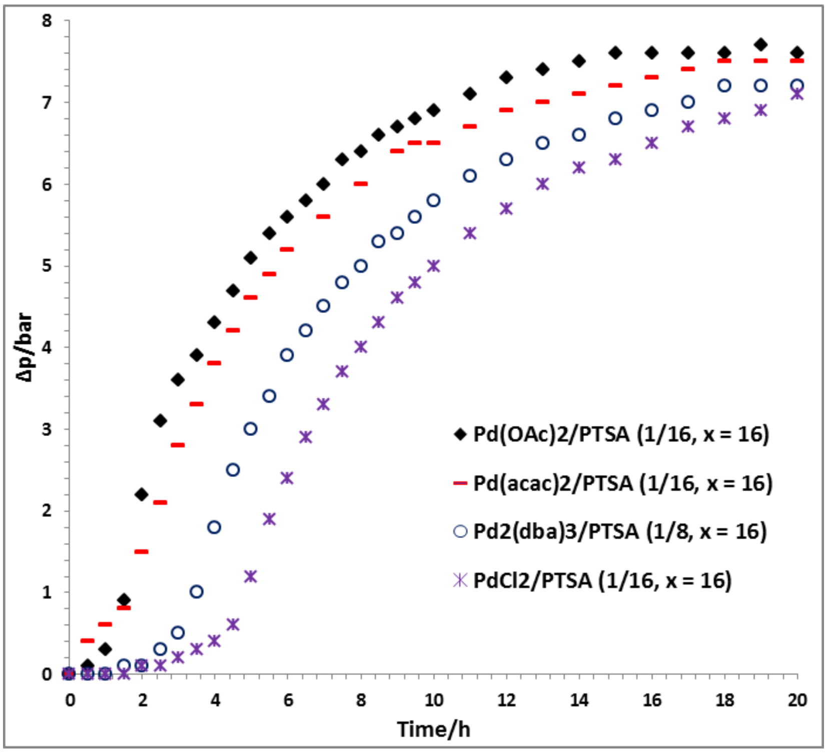

(a) The effect of palladium precursors

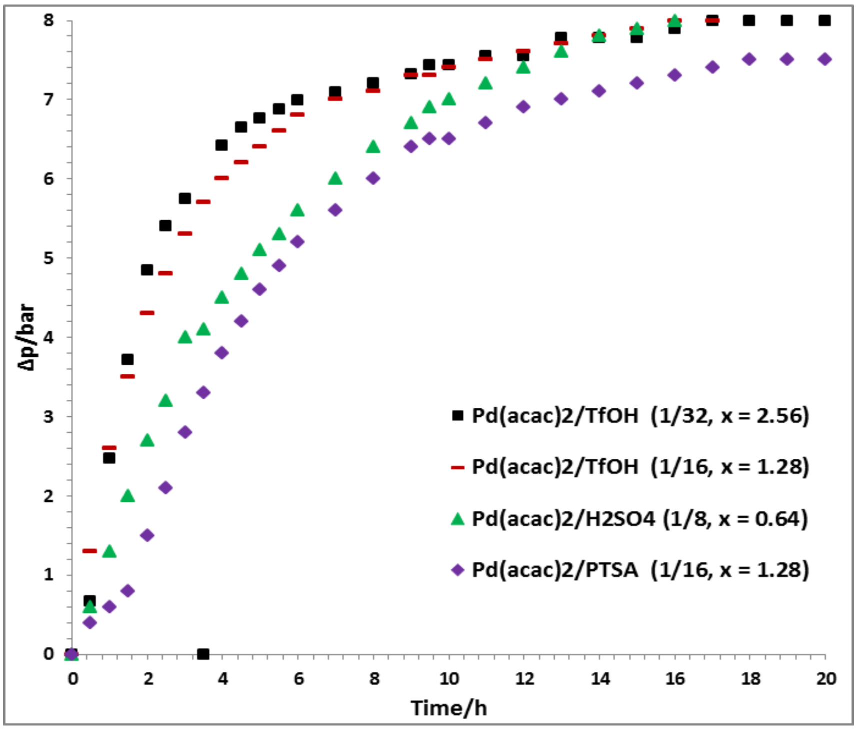

(b) The effect of co-acids

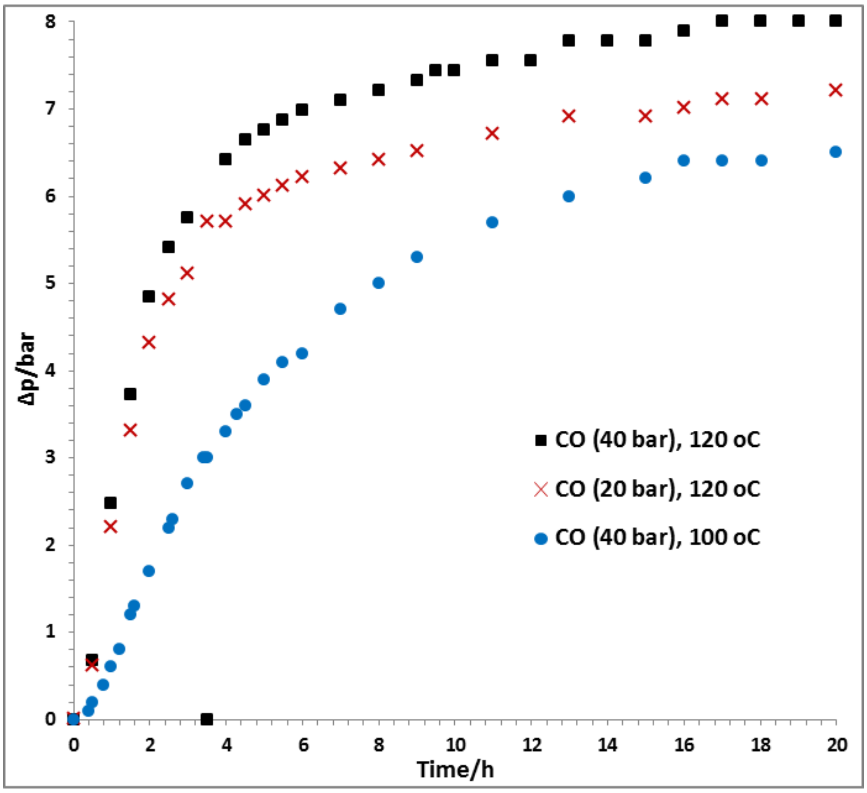

(c) The effect of CO pressure and reaction temperature

Supplementary Figure 9. The effect of various parameters for Pd-catalyzed alkoxycarbonylation of tetramethylethylene 1a.

(a) The effect of palladium precursors: Under argon atmosphere, Pd precursor (0.08 mol% Pd), **L3** (27.9 mg, 0.32 mol%), and PTSA (48.6 mg, 1.28 mol%) were added into an autoclave (100 mL). Then MeOH (20 mL) and **1a** (2.4 mL, 20 mmol) were injected via syringe. The autoclave was flushed with CO gas three times and pressurized with CO gas to 40 bar at room temperature. The reaction was performed at 120 °C for 20 h. After the reaction finished, the autoclave was cooled to room temperature and the pressure was carefully released. The yield of **2a** was measured by GC analysis using isooctane (3.0 mL) as the internal standard.

(b) The effect of co-acids: Under argon atmosphere, $Pd(acac)_2$ (4.9 mg, 0.08 mol% Pd) and **L3** (27.9 mg, 0.32 mol%) were added into an autoclave (100 mL). Then MeOH (20 mL), acid (0.64-2.56 mol%), and **1a** (2.4 mL, 20 mmol) were injected via syringe. The autoclave was flushed with CO gas three times and pressurized with CO gas to 40 bar at room temperature. The reaction was performed at 120 °C for 20 h. After the reaction finished, the autoclave was cooled to room temperature and the pressure was carefully released. The yield of **2a** was measured by GC analysis using isooctane (3.0 mL) as the internal standard.

(c) The effect of CO pressure and reaction temperature: Under argon atmosphere, $Pd(acac)_2$ (4.9 mg, 0.08 mol% Pd) and **L3** (27.9 mg, 0.32 mol%) were added into an autoclave (100 mL). Then MeOH (20 mL), CF_3SO_2OH (45 uL, 2.56 mol%), and **1a** (2.4 mL, 20 mmol) were injected into the autoclave. The autoclave was flushed with CO gas three times and pressurized with CO gas to desired pressure at room temperature. The reaction was performed at preset temperature for 20 h. After the reaction finished, the autoclave was cooled to room temperature and the pressure was carefully released. The yield of **2a** was measured by GC analysis using isooctane (3.0 mL) as the internal standard.

Palladium/L3-catalyzed alkoxycarbonylation of alkenes 1a-1w

Pd-catalyzed alkoxycarbonylation of tetramethylethylene 1a with different alcohols.: Under argon atmosphere, $Pd(acac)_2$ (4.9 mg, 0.08 mol% Pd) and **L3** (27.9 mg, 0.32 mol%) were added into an autoclave (100 mL). Then alcohol (20 mL), CF_3SO_2OH (45 uL, 2.56 mol%), and **1a** (2.4 mL, 20 mmol) were injected into the autoclave. The autoclave was flushed with CO gas three times and pressurized with CO gas to 40 bar at room temperature. The reaction was performed at 120 ^oC for 20 h. After the reaction finished, the autoclave was cooled to room temperature and the pressure was carefully released. The products **2a** and **4-6** were isolated in 91-99% yields through distillation or column chromatography on silica gel.

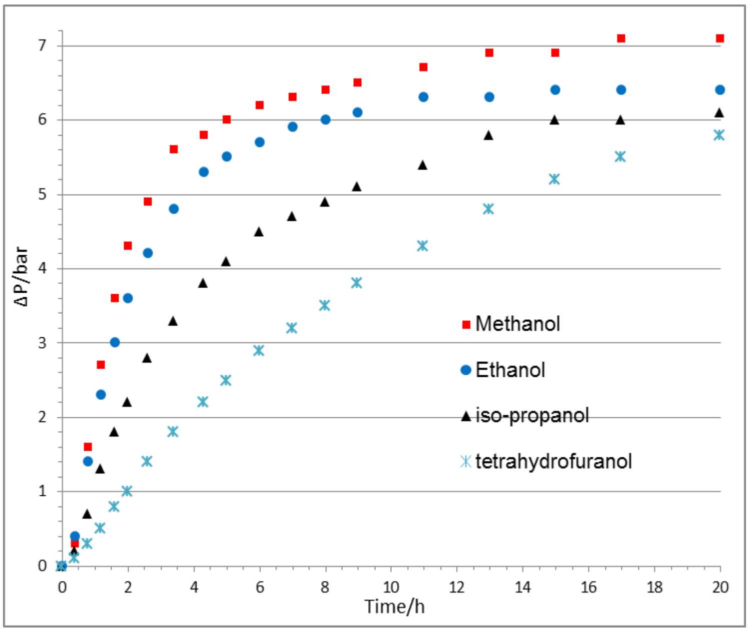

Supplementary Figure 10. Pd-catalyzed alkoxycarbonylation of 1a with different alcohols.

Pd-catalyzed methoxycarbonylation of 1e-1w (0.1 mol% Pd): Under argon atmosphere, a vial (4 mL) was charged with $[Pd(acac)_2]$ (0.61 mg, 0.1 mol%), **L3** (3.5 mg, 0.4 mol%), PTSA (6.1 mg, 1.6 mol%) and a stirring bar. Then MeOH (2.0 mL) and the alkene **1** (2.0 mmol) were added. The vial was placed in an alloyed plate, which was then transferred into an autoclave (300 mL) under argon atmosphere. The autoclave was flushed with CO three times at room temperature and then pressurized with CO to 40 bar. The reaction was performed at 120 ^oC for 20 h. After the reaction finished, the autoclave was cooled to room temperature and the pressure was carefully released. The regionselectivity of the product was measured by GC analysis. The desired ester **2** was purified through column chromatography on silica gel.

Pd-catalyzed methoxycarbonylation of 1b, 1c, and 1x (0.5 mol% Pd): Under argon atmosphere, a vial (4 mL) was charged with [Pd(acac)$_2$] (3.04 mg, 0.5 mol%), **L3** (17.4 mg, 2 mol%), PTSA (30.4 mg, 8 mol%) and a stirring bar. Then MeOH (2.0 mL) and the alkene **1** (2.0 mmol) were added. The vial was placed in an alloyed plate, which was then transferred into an autoclave (300 mL) under argon atmosphere. The autoclave was flushed with CO three times at room temperature and then pressurized with CO to 40 bar. The reaction was performed at 120 ^{o}C for 20 h (50 h for **2x**). After the reaction finished, the autoclave was cooled to room temperature and the pressure was carefully released. The regioselectivity of the product was measured by GC analysis. The desired ester **2** was purified through column chromatography on silica gel.

Pd-catalyzed methoxycarbonylation of 1d and 1e (0.04 mol% Pd): A 100 mL steel autoclave was charged with Pd(acac)$_2$ (6.1 mg, 0.04 mol%), **L** (0.16 mol%), and PTSA (61 mg, 0. 64 mol%) under argon atmosphere. Methanol (20 mL) and **1d** or **1e** (50 mmol) were injected into the autoclave via syringe. Then the autoclave was flashed with CO gas three times and pressurized with CO to 40 bar. After the reaction was carried out at 120 ^{o}C for desired time, the autoclave was cooled to room temperature and depressurized slowly. The product was isolated via distillation of the reaction mixture.

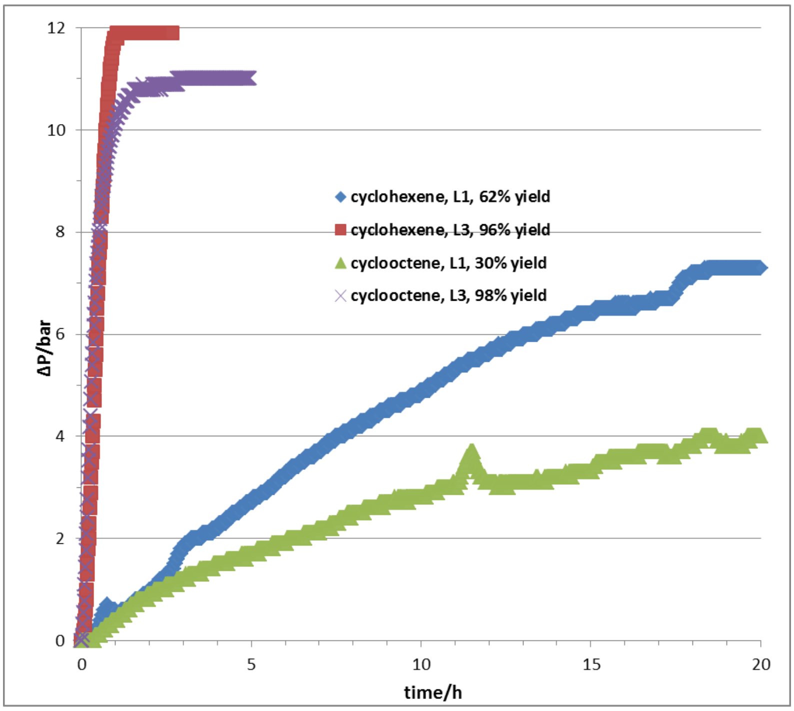

Supplementary Figure 11. Methoxycarbonylation of 1d and 1e

Pd-catalyzed methoxycarbonylation of 1z (4 mol% Pd): Under argon atmosphere, a vial (10 mL) was charged with [Pd(acac)$_2$] (3.04 mg, 1 mol%), **L3** (17.4 mg, 4 mol%), PTSA

(15.2 mg, 8 mol%), **1z** (100 mg, 0.26 mmol), and a stirring bar. Then MeOH (5.0 mL) was injected into the vial via syringe. The vial was placed in an alloyed plate, which was then transferred into an autoclave (300 mL) under argon atmosphere. The autoclave was flushed with CO three times at room temperature and then pressurized with CO to 40 bar. The reaction was performed at 120 ^{o}C for 90 h. After the reaction finished, the autoclave was cooled to room temperature and the pressure was carefully released. The regioselectivity of the product was measured by GC analysis. The desired ester **2z** was isolated in 81% yield through column chromatography on silica gel.

Pd/L3-catalyzed methoxycarbonylation of ethylene, propylene, and dibutene

Pd-catalyzed methoxycarbonylation of ethylene and propylene at 80 °C: A 100 mL steel autoclave was charged with $Pd(acac)_2$ (6.52 mg, 0.04 mol%), **L3** (37.4 mg, 0.16 mol%), and PTSA (61.1 mg, 0.6 mol%) under argon atmosphere. Methanol (20 mL) was injected into the autoclave via syringe. Then ethylene (1.5 g, 53.6 mmol) or propylene (2.3 g, 54.8 mmol) was introduced into the autoclave (mass control by balance). After the reaction mixture was heated to 80 °C, CO (30 bar) was introduced into the autoclave and the reaction was carried out at 80 °C for 20 h. The autoclave was cooled to room temperature and depressurized slowly. The content was transferred to a 50 mL Schlenk flask and isooctane (internal standard, 3.0 mL) was added into the solution. The yield was measured by GC analysis.

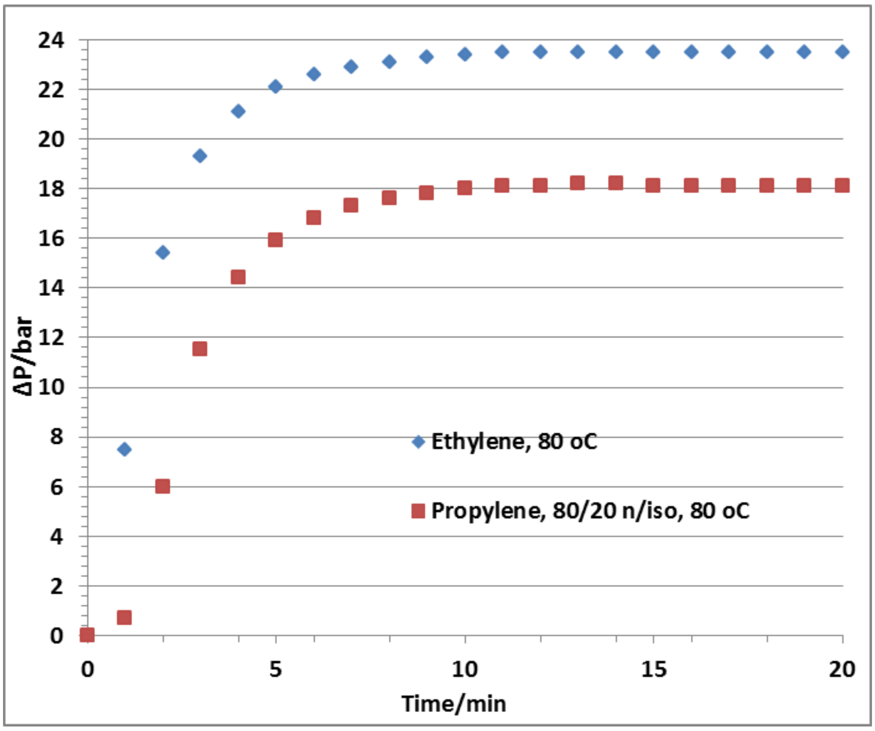

Supplementary Figure 12. Methoxycarbonylation of ethylene and propylene.

Ethylene methoxycarbonylation at 23 °C: A 100 mL steel autoclave was charged with $Pd(acac)_2$ (6.52 mg, 0.04 mol%), **L3** (37.4 mg, 0.16 mol%), and PTSA (61.1 mg, 0.6 mol%) under argon atmosphere. Methanol (20 mL) was injected into the autoclave via syringe. Then ethylene (1.5 g, 53.6 mmol) was introduced into the autoclave (mass control by balance). CO (30 bar) was introduced into the autoclave at 23 °C and the reaction was carried out for 20 h. The autoclave was depressurized slowly. The content was transferred to a 50 mL Schlenk flask and isooctane (internal standard, 3.0 mL) was added into the solution. The yield was measured by GC analysis.

Ethylene methoxycarbonylation at 120 °C: A 300 mL steel autoclave was charged with $Pd(acac)_2$ (0.152 mg, 5 x 10^{-4} mmol, 0.152 mg/mL solution in MeOH), **L3** (109 mg, 0.25 mmol), and PTSA (190 mg, 1.0 mmol) under argon atmosphere. Methanol (50 mL) was injected into the autoclave via syringe. The weight of the autoclave was determined. Then ethylene (11.7 g, circa 20 bar, 418 mmol) was introduced into the autoclave (mass control by balance). CO (30 bar, circa 12.1 g, 432 mmol) was introduced into the autoclave and the reaction was carried out at 120 °C for 18 h. Significant gas consumption was observed. The autoclave was cooled to room temperature and depressurized slowly. The autoclave was weighted after the reaction and 22.1 g mass addition of the autoclave was detected controlled by balance. TOF (turnover frequency): >44 000 h^{-1} was calculated for 18 h. Then ethylene (11.7 g, circa 20 bar, 418 mmol) was introduced into the autoclave (mass control by balance). CO (30 bar, circa 12.2 g, 436 mmol) was introduced into the autoclave and the reaction was carried out at 120 °C for 40 h. After the autoclave was cooled to room temperature and depressurized slowly, we weighted the autoclave again and 17.8 g mass addition of the autoclave was observed controlled by balance. The product methyl propionate was confirmed by GC analysis (>99% selectivity). Total 39.9 g mass addition of the autoclave was determined which corresponded to 62.7 g of the desired product. Total TON (turnover number): >1 425000 for two portions.

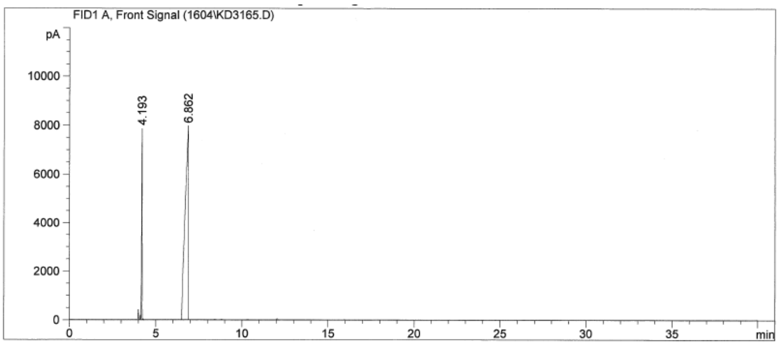

Area Percent Report

Sorted By : Signal
Multiplier: : 1.0000
Dilution: : 1.0000
Use Multiplier & Dilution Factor with ISTDs

Signal 1: FID1 A, Front Signal

Peak #	RetTime [min]	Type	Width [min]	Area [pA*s]	Height [pA]	Area %
1	4.193	VB S	0.0382	1.81856e4	7852.83643	15.18203
2	6.862	BB	0.1532	1.01598e5	7945.48047	84.81797

Pd/L3-catalyzed methoxycarbonylation of dibutene: A 100 mL steel autoclave was charged with $Pd(acac)_2$ (5.9 mg, 0.04 mol%), **L3** (33.5 mg, 0.16 mol%), and PTSA (54.7 mg, 0. 6 mol%) under argon atmosphere. Methanol (30 mL) and dibutene (7.54 mL, 48 mmol) were injected into the autoclave via syringe. Then the autoclave was flashed with CO gas

three times and pressurized with CO to 40 bar. After the reaction was carried out at 120 ^{o}C for 20 h, the autoclave was cooled to room temperature and depressurized slowly. The content was transferred to a 50 mL Schlenk flask and isooctane (internal standard, 10 mL) was added into the solution. The yield and regioselectivity were measured by GC analysis.

Characterization of products 2a-5a and 2b-2z

methyl 3,4-dimethylpentanoate, 2a (*1*)

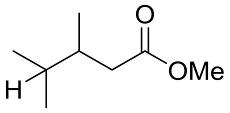

Colorless oil, 99% yield. ^{1}H NMR (400 MHz, $CDCl_3$): δ 3.65 (s, 3H), 2.33 (dd, *J* = 20.0, 8.0 Hz, 1H), 2.06 (dd, *J* = 20.0, 8.0 Hz, 1H), 1.89-1.83 (m, 1H), 1.59-1.53 (m, 1H), 0.87-0.81 (m, 9H). ^{13}C NMR (100 MHz, $CDCl_3$): δ 174.3, 51.5, 39.1, 36.0, 32.2, 19.9, 18.8, 15.9.

ethyl 3,4-dimethylpentanoate, 4 (*2*)

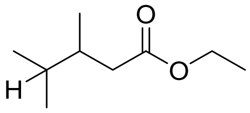

Colorless oil, 98% yield. ^{1}H NMR (300 MHz, $CDCl_3$): δ 4.11 (q, *J* = 9.0 Hz, 2H), 2.31 (dd, *J* = 15.0, 6.0 Hz, 1H), 2.04 (dd, *J* = 15.0, 9.0 Hz, 1H), 1.90-1.83 (m, 1H), 1.60-1.51 (m, 1H), 1.24 (d, *J* = 6.0 Hz, 3H), 0.87-0.82 (m, 9H). ^{13}C NMR (100 MHz, $CDCl_3$): δ 173.7, 60.0, 39.2, 35.8, 32.0, 19.8, 18.2, 15.7, 14.2.

isopropyl 3,4-dimethylpentanoate, 5

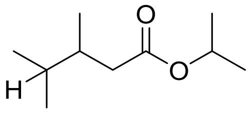

Colorless oil, 96% yield. ^{1}H NMR (300 MHz, $CDCl_3$): δ 4.99 (sept, *J* = 6.0 Hz, 1H), 2.28 (dd, *J* = 15.0, 6.0 Hz, 1H), 2.01 (dd, *J* = 15.0, 9.0 Hz, 1H), 1.89-1.82 (m, 1H), 1.63-1.48 (m, 1H), 1.21 (d, *J* = 6.0 Hz, 6H), 0.87-0.81 (m, 9H). ^{13}C NMR (75 MHz, $CDCl_3$): δ 173.2, 67.2, 39.5, 35.9, 32.1, 21.8, 21.7, 19.8, 18.2, 15.6. HRMS (EI): Calcd. for $C_{10}H_{20}O_2$ $[M]^+$: 172.14578, Found: 172.14572.

(tetrahydrofuran-2-yl)methyl 3,4-dimethylpentanoate, 6

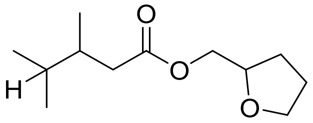

Colorless oil, 91% yield. ^{1}H NMR (300 MHz, $CDCl_3$): δ 4.11-3.99 (m, 2H), 3.94-3.88 (m, 1H), 3.84-3.77 (m, 1H), 3.75-3.68 (m, 1H), 2.31 (dd, *J* = 15.0, 3.0 Hz, 1H), 2.04 (dd, *J* = 15.0, 9.0 Hz, 1H), 1.98-1.78 (m, 4H), 1.58-1.45 (m, 2H), 0.81-0.75 (m, 9H). ^{13}C NMR (100 MHz, $CDCl_3$): δ 173.4, 76.3, 68.2, 66.0, 38.8, 35.6, 35.6, 31.9, 27.8, 25.5, 19.6, 18.0, 15.5. HRMS (ESI): Calcd. for $C_{12}H_{22}O_3$ $[M + Na]^+$: 237.14612, Found: 237.14660.

methyl decahydronaphthalene-2-carboxylate, 2b (*3*)

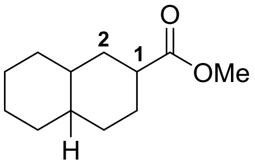

Colorless oil, 94% yield, **1/2** = >99/1. ^{1}H NMR (300 MHz, $CDCl_3$): δ 3.66-3.65 (m, 3H, OCH_3), 2.45-2.28 (m, 1H, CH), 1.71-0.91 (m, 16H). ^{13}C NMR (75 MHz, $CDCl_3$): δ 176.5, 51.4, 43.4, 42.5, 42.3, 36.2, 33.7, 33.6, 32.9, 29.0, 26.5. HRMS (EI): Calcd. for $C_{12}H_{20}O_2$ $[M]^+$: 196.14633, Found: 196,14570.

methyl 2,3-dihydro-1H-indene-1-carboxylate, 2c (*4*)

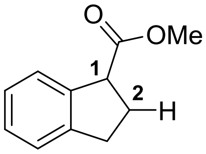

Colorless oil, 69% yield, **1**/**2** = 89/11. ^{1}H NMR (300 MHz, $CDCl_3$) δ = 7.29-7.26 (m, 1H), 7.15-7.03 (m, 3H), 3.96 (t, J = 9.0 Hz, 1H), 3.62 (s, 3H), 3.00-2.80 (m, 2H), 2.40-2.16 (m, 2H); ^{13}C NMR (75 MHz, $CDCl_3$) δ = 175.72, 174.36, 144.10, 141.55, 140.70, 127.56, 126.63, 126.45, 124.94, 124.80, 124.70, 124.34, 52.01, 51.92, 50.11, 43.50, 36.21, 31.79, 28.78; HRMS (ESI): Calcd. for $C_{11}H_{12}O_2$ $[M+1]^+$: 177.09101, Found: 177.09115.

methyl cyclohexanecarboxylate, 2d (*5*)

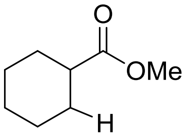

Colorless oil, 96% yield. ^{1}H NMR (300 MHz, $CDCl_3$) δ = 3.60 (s, 3H), 2.24 (tt, J = 12, 3 Hz, 1H), 1.86-1.81 (m, 2H), 1.71-1.55 (m, 3H), 1.44-1.20 (m, 5H); ^{13}C NMR (75 MHz, $CDCl_3$) δ = 176.52, 51.42, 43.12, 29.05, 25.79, 25.48.

methyl cyclooctanecarboxylate, 2e (*6*)

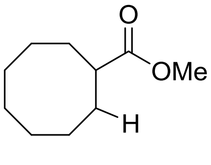

Colorless oil, 98% yield. ^{1}H NMR (300 MHz, $CDCl_3$) δ = 3.60 (s, 3H), 2.56-2.47 (m, 1H), 1.92-1.82 (m, 2H), 1.75-1.64 (m, 4H), 1.59-1.48 (m, 8H); ^{13}C NMR (75 MHz, $CDCl_3$) δ = 177.8, 51.5, 43.4, 28.7, 26.7, 26.1, 25.2; HRMS (EI): Calcd. for $C_{10}H_{18}O_2$ $[M]^+$: 170.13013, Found: 170.12966.

methyl 3-phenylbutanoate, 2f (*7*)

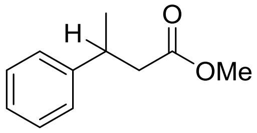

Colorless oil, 95% yield. ^{1}H NMR (300 MHz, $CDCl_3$) δ = 7.34-7.18 (m, 5H), 3.64 (s, 3H), 3.33-3.26 (m, 1H), 2.68-2.52 (m, 2H), 1.31 (d, J = 9.0 Hz, 3H); ^{13}C NMR (75 MHz, $CDCl_3$) δ = 172.82, 145.66, 128.47, 126.67, 126.38, 51.48, 42.71, 36.40, 21.75; HRMS (EI): Calcd. for $C_{11}H_{14}O_2$ $[M]^+$: 178.09883, Found: 178.09866.

methyl 3-(*o*-tolyl)butanoate, 2g (*8*)

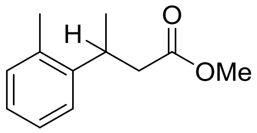

Colorless oil, 96% yield. ^{1}H NMR (300 MHz, $CDCl_3$) δ = 7.24-7.12 (m, 4H), 3.68 (s, 3H), 3.64-3.55 (m, 1H), 2.70 (dd, J = 15.0, 6.0 Hz, 1H), 2.59 (dd, J = 15.0, 9.0 Hz, 1H), 2.44 (s, 3H), 1.32 (d, J = 6.0 Hz, 3H); ^{13}C NMR (75 MHz, $CDCl_3$) δ = 172.8, 143.7, 135.0, 130.3, 126.1, 125.9, 124.8, 124.7, 51.3, 41.7, 31.2, 21.1, 19.2; HRMS (ESI): Calcd. for $C_{12}H_{16}O_2$ $[M + Na]^+$: 215.10425, Found: 215.10396.

methyl 3-(4-fluorophenyl)butanoate, 2h (*9*)

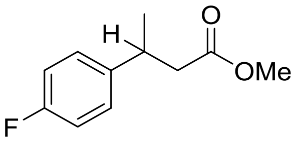

Colorless oil, 96% yield. ^{1}H NMR (300 MHz, $CDCl_3$) δ = 7.20-7.14 (m, 2H), 7.00-6.93 (m, 2H), 3.60 (s, 3H), 3.31-3.23 (m, 1H), 2.62-2.48 (m, 2H), 1.27 (d, J = 9.0 Hz, 3H); ^{13}C NMR (75 MHz, $CDCl_3$) δ = 172.4, 161.3 (d, J = 243 Hz), 141.2, 127.9, 115.1 (d, J = 21Hz), 51.3, 42.6, 35.6, 21.7; HRMS (ESI): Calcd. for $C_{11}H_{13}FO_2$ $[M + H]^+$: 197.09723, Found: 197.09701.

methyl 3-(4-chlorophenyl)butanoate, 2i (*7*)

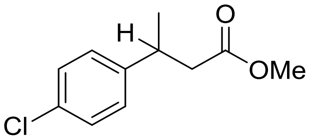

Colorless oil, 97% yield. ^{1}H NMR (300 MHz, $CDCl_3$) δ = 7.88-7.85 (m, 3H), 7.73 (s, 1H), 7.54-7.27 (m, 3H), 3.68 (s, 3H), 3.68-3.48 (m, 1H), 2.85-2.67 (m, 2H), 1.46 (d, J = 6.0 Hz,

3H); ^{13}C NMR (75 MHz, $CDCl_3$) δ = 172.6, 143.0, 133.4, 132.2, 128.0, 127.5, 127.4, 125.8, 125.3, 125.2, 124.8, 51.38, 42.5, 36.4, 21.7; HRMS (EI): Calcd. for $C_{11}H_{13}O_2Cl$ $[M]^+$: 212.05986, Found: 212.05972.

methyl 3-(naphthalen-2-yl)butanoate, 2j (*7*)

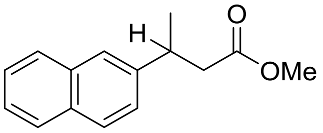

Colorless oil, 97% yield. ^{1}H NMR (300 MHz, $CDCl_3$) δ = 7.33-7.28 (m, 2H), 7.22-7.17 (m, 2H), 3.66 (s, 3H), 3.34-3.25 (m, 1H), 2.62-2.59 (m, 2H), 1.32 (d, J = 9.0 Hz, 3H); ^{13}C NMR (75 MHz, $CDCl_3$) δ = 172.43, 144.03, 131.95, 128.53, 128.04, 51.47, 42.47, 35.80, 21.73; HRMS (ESI): Calcd. for $C_{15}H_{16}O_2$ $[M + H]^+$: 229.12231, Found: 229.12179.

methyl 3,3-diphenylpropanoate, 2k (*10*)

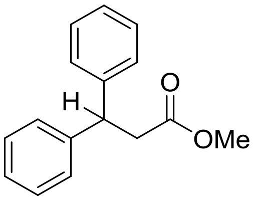

Colorless oil, 95% yield. ^{1}H NMR (300 MHz, $CDCl_3$) δ = 7.43-7.35 (m, 8H), 7.34-7.27 (m, 2H), 4.70 (t, J = 9.0 Hz, 3H), 3.68 (s, 3H), 3.19 (d, J = 9.0 Hz, 2H); ^{13}C NMR (75 MHz, $CDCl_3$) δ = 172.08, 143.34, 128.43, 127.51, 126.41, 51.50, 46.83, 40.42; HRMS (EI): Calcd. for $C_{16}H_{16}O_2$ $[M]^+$: 240.11448, Found: 240.11401.

dimethyl 3,3'-(1,3-phenylene)dibutyrate, 2l

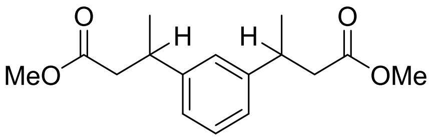

Colorless oil, 99% yield. ^{1}H NMR (300 MHz, $CDCl_3$) δ = 7.39-7.33 (m, 1H, Ar), 7.27-7.17 (m, 3H, Ar), 3.74 (s, 6H, OCH_3), 3.42-3.33 (m, 2H, CH), 2.78-2.62 (m, 4H, CH_2), 1.41 (d, J = 6.0 Hz, 6H, CH_3); ^{13}C NMR (75 MHz, $CDCl_3$) δ = 172.7, 145.8, 128.5, 125.2, 124.5, 51.4, 42.7, 36.3, 21.6; HRMS (EI): Calcd. for $C_7H_{12}O_4$ $[M]^+$: 203.07882, Found: 203.07939.

methyl 3-(triethylsilyl)propanoate, 2m (*11*)

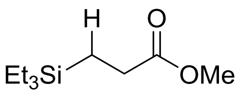

Colorless oil, 88% yield, *n*/*iso* = >99/1. ^{1}H NMR (300 MHz, $CDCl_3$) δ = 3.67 (s, 3H, OCH_3), 2.31-2.25 (m, 2H, CH_2), 0.96-0.84 (m, 11H), 0.56-0.48 (m, 6H); ^{13}C NMR (75 MHz, $CDCl_3$) δ = 175.6, 51.5, 28.6, 7.3, 6.5, 3.0.

methyl 4,4,5,5,6,6,7,7,8,8,9,9,10,10,11,11,11-heptadecafluoroundecanoate, 2n (*12*)

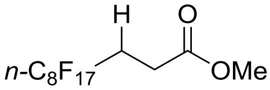

Colorless oil, 99% yield, *n*/*iso* = >99/1. ^{1}H NMR (400 MHz, $CDCl_3$): δ 3:72 (s, 3H), 2.53-2:40 (m, 2H). ^{3}C NMR (100 MHz, $CDCl_3$): δ 171.7, 118.0-109.0 (broad, 8C), 51.2, 25.7 (t, *J* = 22 Hz), 24.4 (t, *J* = 4.5 Hz).

methyl 7-hydroxyheptanoate, 2o (*13*)

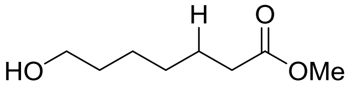

Colorless oil, 80% yield, *n*/*iso* = 66/34. ^{1}H NMR (400 MHz, $CDCl_3$) δ = 3.63-3.61 (m, 3H), 3.56 (t, *J* = 8.0 Hz, 2H), 2.44-2.37 (m, 0.3H), 2.31 (s, 1H), 2.26 (t, *J* = 8.0 Hz, 1.4H), 1.62-1.47 (m, 4H), 1.32-1.30 (m, 3.4H), 1.10 (d, *J* = 8.0 Hz, 0.9 Hz); ^{13}C NMR (100 MHz, $CDCl_3$) δ = 177.4, 174.4, 62.7, 62.5, 51.6, 51.5, 50.5, 39.5, 34.0, 32.5, 28.9, 25.4, 24.9, 23.5, 17.1.

methyl 6-cyanohexanoate, 2p (*14*)

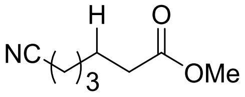

Yellowish oil, 65% yield, *n*/*iso* = 84/16. ^{1}H NMR (300 MHz, $CDCl_3$) δ = 3.57 (s, 3H), 2.30-2.22 (m, 4H), 1.61-1.52 (m, 4H), 1.44-1.34 (m, 2H); ^{13}C NMR (75 MHz, $CDCl_3$) δ = 175.92, 173.32, 119.32, 119.11, 51.34, 51.19, 38.38, 33.24, 32.18, 27.73, 24.73, 23.69, 22.85, 16.74, 16.61; HRMS (ESI): Calcd. for $C_8H_{13}O_2N$ $[M + Na]^+$: 178.08385, Found: 178.08401.

methyl 7-chloroheptanoate, 2q (*15*)

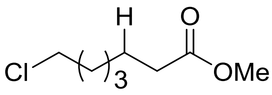

Colorless oil, 91% yield, *n*/*iso* = 70/30. ^{1}H NMR (300 MHz, $CDCl_3$) δ = 3.58 (s, 2.82H), 3.44 (t, *J* = 6.0 Hz, 1.64H), 3.29-3.23 (m, 0.77H), 2.38-2.18 (m, 1.73H), 1.73-1.06 (m, 8H); ^{13}C NMR (75 MHz, $CDCl_3$) δ = 176.66, 173.87, 173.74, 72.47, 58.23, 51.16, 44.66, 44.47, 33.63, 32.72, 32.20, 32.15, 28.12, 26.27, 24.49, 24.31, 16.81.

dimethyl dodecanedioate, dimethyl 2-methylundecanedioate, 2r (*16*)

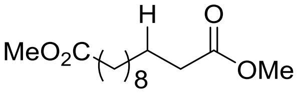

Colorless oil, 96% yield, *n*/*iso* = 72/28. ^{1}H NMR (300 MHz, $CDCl_3$) δ = 3.61 (s, 6H), 2.25 (t, *J* = 9.0 Hz, 4H), 1.58-1.51 (m, 4H), 1.22 (s, br, 12H); ^{13}C NMR (75 MHz, $CDCl_3$) δ = 177.1, 174.1, 51.4, 39.4, 34.0, 33.9, 29.2, 29.1, 29.0, 27.1, 24.8, 17.0; HRMS (ESI): Calcd. for $C_{14}H_{26}O_4$ $[M + H]^+$: 259.19039, Found: 259.19025.

methyl 3-(1,3-dioxoisoindolin-2-yl)propanoate, 2s (*7*)

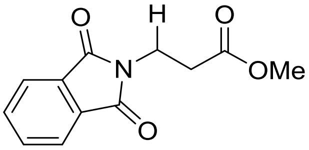

White solid, 99% yield, *n*/*iso* = >99/1. ^{1}H NMR (400 MHz, $CDCl_3$): δ 7.80-7.78 (m, 2H), 7.68-7.66 (m, 2H), 3.94 (t, *J* = 8.0 Hz, 2H), 3.63 (s, 3H), 2.68 (t, *J* = 8.0 Hz, 3H). ^{13}C NMR (100 MHz, $CDCl_3$): δ 171.0, 167.7, 133.9, 131.9, 131.8, 123.1, 51.7, 33.6, 32.6.

methyl 3-(4-(2-methoxy-2-oxoethyl)cyclohexyl)butanoate, 2t

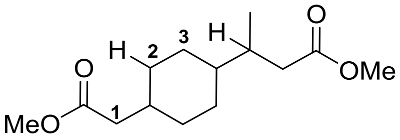

Colorless oil, 85% yield, **1**/(**2**+**3**) = 56/44. ^{1}H NMR (400 MHz, $CDCl_3$) δ = 3.65-3.63 (m, 6H), 2.31-0.82 (m, 18H); ^{13}C NMR (100 MHz, $CDCl_3$) δ = 176.4, 174.0, 173.8, 173.7, 173.4, 51.4, 51.3, 42.0, 41.4, 41.3, 39.0, 35.1, 35.0, 34.9, 34.5, 33.5, 29.6, 28.2, 20.2, 16.5; HRMS (ESI): Calcd. for $C_{14}H_{24}O_4$ $[M]^+$: 256.16746, Found: 256.16637.

dimethyl acetylaspartate, 2u (*17*)

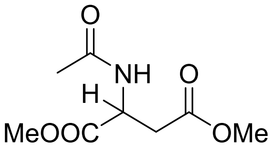

White solid, 88% yield, *n*/*iso* = >99/1. ^{1}H NMR (300 MHz, $CDCl_3$): δ 6.71-6.69 (m, 1H, NH), 4.83-4.77 (m, 1H, CH), 3.69 (s, 3H, OCH_3), 3.63 (s, 3H, OCH_3), 2.96 (dd, *J* = 18.0, 6.0 Hz, 1H, CH_2), 2.80 (dd, *J* = 18.0, 3.0 Hz, 1H, CH_2), 1.97 (s, 3H, $COCH_3$). ^{13}C NMR (75 MHz, $CDCl_3$): δ 171.3, 171.1, 169.8, 52.6, 51.8, 48.3, 35.9, 22.8. HRMS (EI): Calcd. for $C_8H_{13}O_5N$ $[M]^+$: 203.07882, Found: 203.07939.

methyl 4,5-bis(4-hydroxyphenyl)heptanoate, 2v

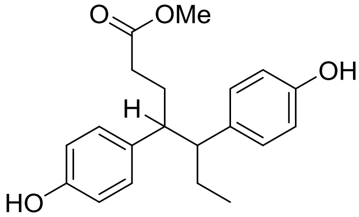

White solid, 92% yield, n/iso = >99/1. ^{1}H NMR (300 MHz, $CDCl_3$): δ 7.02-6.61 (m, 8H, Ar), 5.85 (s, br, 2H, OH), 3.61 (s, 1H, OMe), 3.52 (s, 2H, OMe), 2.73-2.42 (m, 2H, CH_2), 2.14-1.24 (m, 6H, CH_2, CH), 0.72 (t, J = 6.0 Hz, 1H, CH_3), 0.53 (t, J = 6.0 Hz, 2H, CH_3). ^{13}C NMR (75 MHz, $CDCl_3$): δ 175.45, 175.43, 154.20, 153.98, 153.85, 153.59, 135.58, 135.08, 134.18, 133.34, 130.02, 129.97, 129.24, 115.36, 115.21, 114.66, 114.49, 53.48, 52.65, 51.79, 51.71, 50.86, 49.93, 32.54, 32.45, 29.53, 28.62, 27.25, 26.00. HRMS (EI): Calcd. for $C_{20}H_{24}O_4$ $[M]^+$: 328.16746, Found: 328.16713.

methyl (3*S*,8*S*,9*S*,10*R*,13*R*,14*S*,17*R*)-3-hydroxy-10,13-dimethyl-17-((*R*)-6-methylheptan-2-yl)hexadecahydro-1*H*-cyclopenta[a]phenanthrene-6-carboxylate, 2w

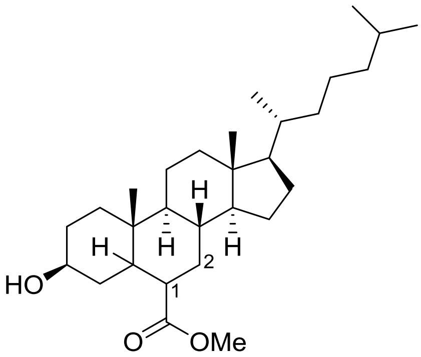

While solid, 81% yield, **1**/**2** = >99/1. ^{1}H NMR (400 MHz, $CDCl_3$): δ 3.65 (s, 3H), 3.62-3.54 (m, 1H), 2.32 (dt, J = 4.0, 12.0 Hz, 1H), 1.97 (td, J = 4.0, 12.0 Hz, 1H), 1.85-1.71 (m, 4H), 1.54-1.49 (m, 5H), 1.42-1.20 (m, 9H), 1.18-0.95 (m, 10H), 0.89 (d, J = 8.0 Hz, 3H), 0.86 (dd, J = 4.0, 8.0 Hz, 6H), 0.83 (s, 3H), 0.74 (dt, J = 4.0, 12.0 Hz, 1H), 0.64 (s, 3H). ^{13}C NMR (100 MHz, $CDCl_3$): δ 176.54, 70.81, 56.15, 55.95, 53.60, 51.40, 45.94, 44.77, 42.59, 39.80, 39.47, 36.71, 36.12, 35.75, 35.32, 35.26, 34.95, 31.05, 28.18, 27.98, 24.06, 23.82, 22.80, 22.54, 21.13, 18.63, 12.91, 12.02. HRMS (EI): Calcd. for $C_{29}H_{50}O_3$ $[M]^+$: 446.37545, Found: 446.37541.

methyl (3*S*,8*S*,9*S*,10*R*,13*R*,14*S*,17*R*)-3-((4-bromobenzyl)oxy)-10,13-dimethyl-17-((*R*)-6-methylheptan-2-yl)hexadecahydro-1H-cyclopenta[a]phenanthrene-6-carboxylate, 2x

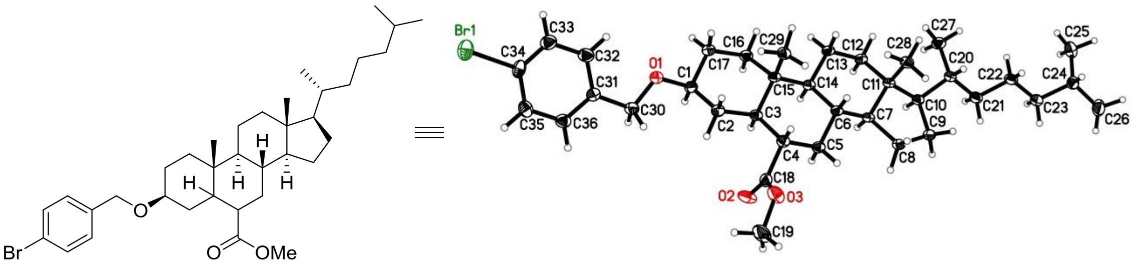

Experimental procedure for the derivation of 2w to 2x: Under argon atmosphere, 44 mg **2w** (0.1 mmol) was dissolved in THF (2 mL). Then 20 mg NaH (60% content, 0.5 mmol) was added to the reaction. After the mixture was stirred at RT for 20 h, 150 mg 4-Bromobenzyl bromide was added. The resulted reaction mixture was stirred at RT for 40 h. The reaction was quenched by 1 mL Water followed by addition of 20 mL water and 20 mL CH_2Cl_2. The mixture was separated and the water phase was extracted with CH_2Cl_2 (2 x 20 mL). The organic phase was combined and dried with sodium sulfate anhydrous. After removal of the solvent, the crude product was purified through column chromatography on silica gel (PE/EA = 50/1-10/1). The desired product **2x** (45 mg) was obtained in 75% yield and characterized by ^{1}H, ^{13}C NMR, HRMS, and X-ray diffraction.

While solid, 75% yield. ^{1}H NMR (300 MHz, $CDCl_3$): δ 7.46-7.42 (m, 2H), 7.22-7.18 (m, 2H), 4.50-4.41 (m, 2H), 3.65 (s, 3H), 3.35-3.28 (m, 1H), 2.32 (dt, *J* = 0.6, 12.0 Hz, 1H), 1.99-1.73 (m, 4H), 1.60-1.49 (m, 5H), 1.40-1.25 (m, 9H), 1.15-0.99 (m, 10H), 0.89 (d, *J* = 6.0 Hz, 3H), 0.86 (dd, *J* = 3.0, 9.0 Hz, 6H), 0.83 (m, 3H), 0.77-0.68 (m, 1H), 0.64 (s, 3H). ^{13}C NMR (75 MHz, $CDCl_3$): δ 176.79, 138.21, 131.55, 129.36, 121.35, 78.12, 69.49, 56.30, 56.11, 53.78, 51.57, 46.06, 44.94, 42.76, 39.96, 39.64, 36.82, 36.28, 35.91, 35.72, 34.62, 31.91, 28.35, 28.15, 28.04, 24.22, 23.98, 22.97, 22.71, 21.27, 18.80, 13.06, 12.18. HRMS (EI): Calcd. for $C_{36}H_{55}O_3Br$ $[M]^+$: 614.33291, Found: 614.33111.

Supplementary Table 4. Crystal data for 2x: Single crystals of **2x** were obtained from the recrystallization in methanol.

Empirical formula	$C_{36}H_{55}BrO_3$
Formula weight	615.71
Temperature	150(2) K
Wavelength	1.54178 Å
Crystal system	monoclinic

Space group	$P2_1$
Unit cell dimensions	a = 11.8286(7) Å α = 90°
	b = 10.6768(6) Å β = 106.603(2)°
	c = 13.7048(8) Å γ = 90°
Volume	1658.64(17) $Å^3$
Z	2
Density (calculated)	1.233 Mg/m^3
Absorption coefficient	1.927 mm^{-1}
F(000)	660
Crystal size	0.260 x 0.107 x 0.052 mm
Theta range for data collection	3.365 to 65.499°
Index ranges	-13<=h<=13, -12<=k<=12, -16<=l<=16
Reflections collected	21047
Independent reflections	5680 [R(int) = 0.0368]
Completeness to theta = 65.499°	99.9 %
Max. and min. transmission	0.91 and 0.63
Data / restraints / parameters	5680 / 1 / 367
Goodness-of-fit on F^2	1.043
Final R indices [$I>2\sigma(I)$]	R1 = 0.0437, wR2 = 0.1207
R indices (all data)	R1 = 0.0446, wR2 = 0.1219
Absolute structure parameter	-0.033(7)
Largest diff. peak and hole	0.824 and -0.300 e.$Å^{-3}$
CCDC no.	1483955

Supplementary References

1. M. Behforouz, T. T. Curran, J. L. Bolan, Regiospecific addition of organocopper reagents to α,β-unsaturated esters. *Tetrahedron Lett.* **27**, 3107 (1986).
2. S. Marquais, M. Alami, G. Cahiez, Manganese-copper-catalyzed conjugate addition of organomagnesium reagents to α,β-ethylenic ketones: preparation of 2-(1,1-dimethylpentyl)-5-methylcyclohexanone from pulegone (cyclohexanone, 2-(1,1-dimethylpentyl)-5-methyl-). *Org. Synth.* **72**, 135 (1995).
3. M. Gordon, S. H. Grover, J. B. Stothers, Carbon-13 nuclear magnetic resonance studies. 29. Carbon-13 spectra of some alicyclic methyl esters. *Can. J. Chem.* **51**, 2092 (1973).
4. R. Deng, L. Sun, Z. Li, Nickel-catalyzed Carboannulation Reaction of o-Bromobenzyl Zinc Bromide with Unsaturated Compounds. *Org. Lett.* **9**, 5207 (2007).
5. Y. N. Cui *et al.*, Photopromoted methoxycarbonylation of olefin with methyl formate by $Co(OAc)_2$. *Chin. Chem. Lett.* **18**, 17 (2007).
6. L. Wu, Q. Liu, R. Jackstell, M. Beller, Ruthenium-catalyzed alkoxycarbonylation of alkenes using carbon monoxide. *Org. Chem. Front.* **2**, 771 (2015).
7. Q. Liu *et al.*, Regioselective Pd-Catalyzed Methoxycarbonylation of Alkenes Using both Paraformaldehyde and Methanol as CO Surrogates. *Angew. Chem. Int. Ed.* **54**, 4493 (2015).
8. G. W. Shipps, Jr. *et al.* (Schering Corporation, USA . 2010), pp. 231.
9. W. Tang, W. Wang, X. Zhang, Phospholane-oxazoline ligands for Ir-catalyzed asymmetric hydrogenation. *Angew. Chem., Int. Ed.* **42**, 943 (2003).
10. S. Oi, Y. Honma, Y. Inoue, Conjugate Addition of Organosiloxanes to α,β-Unsaturated Carbonyl Compounds Catalyzed by a Cationic Rhodium Complex. *Org. Lett.* **4**, 667 (2002).
11. D. Seyferth, R. E. Mammarella, H. A. Klein, New functional allylic lithium reagents: gem-dialkoxyallyllithium reagents: a useful route to β-silyl- and β-stannylpropionate esters. *J. Organomet. Chem.* **194**, 1 (1980).
12. S. Barata-Vallejo, A. Postigo, (Me3Si)3SiH-Mediated Intermolecular Radical Perfluoroalkylation Reactions of Olefins in Water. *J. Org. Chem.* **75**, 6141 (2010).
13. G. Solladie, A. Rubio, M. C. Carreno, J. L. Garcia Ruano, Asymmetric synthesis of orsellinic acid type macrolides: The example of lasiodiplodin. *Tetrahedron: Asymmetry* **1**, 187 (1990).
14. Y. Tamaru, H. Tanigawa, T. Yamamoto, Z.-i. Yoshida, Copper(I)-Promoted Michael-Addition Reaction of Organozincs of Esters, Nitriles, and α-Amino Acids. *Angew. Chem. Int. Ed.* **28**, 351 (1989).
15. Y. Y. Hu *et al.*, Photopromoted carbonylation of 1-bromo-6-chlorohexane under ambient conditions. *Chin. Chem. Lett.* **19**, 529 (2008).
16. M. R. L. Furst, T. Seidensticker, D. J. Cole-Hamilton, Polymerisable di- and triesters from Tall Oil Fatty Acids and related compounds. *Green Chem.* **15**, 1218 (2013).
17. C. Stueckler *et al.*, Stereo-Controlled Asymmetric Bioreduction of α,β-Dehydroamino Acid Derivatives. *Adv. Synth. Catal.* **353**, 1169 (2011).

Supplementary Figure 13. Spectra of products 4-6 and 2a-2x

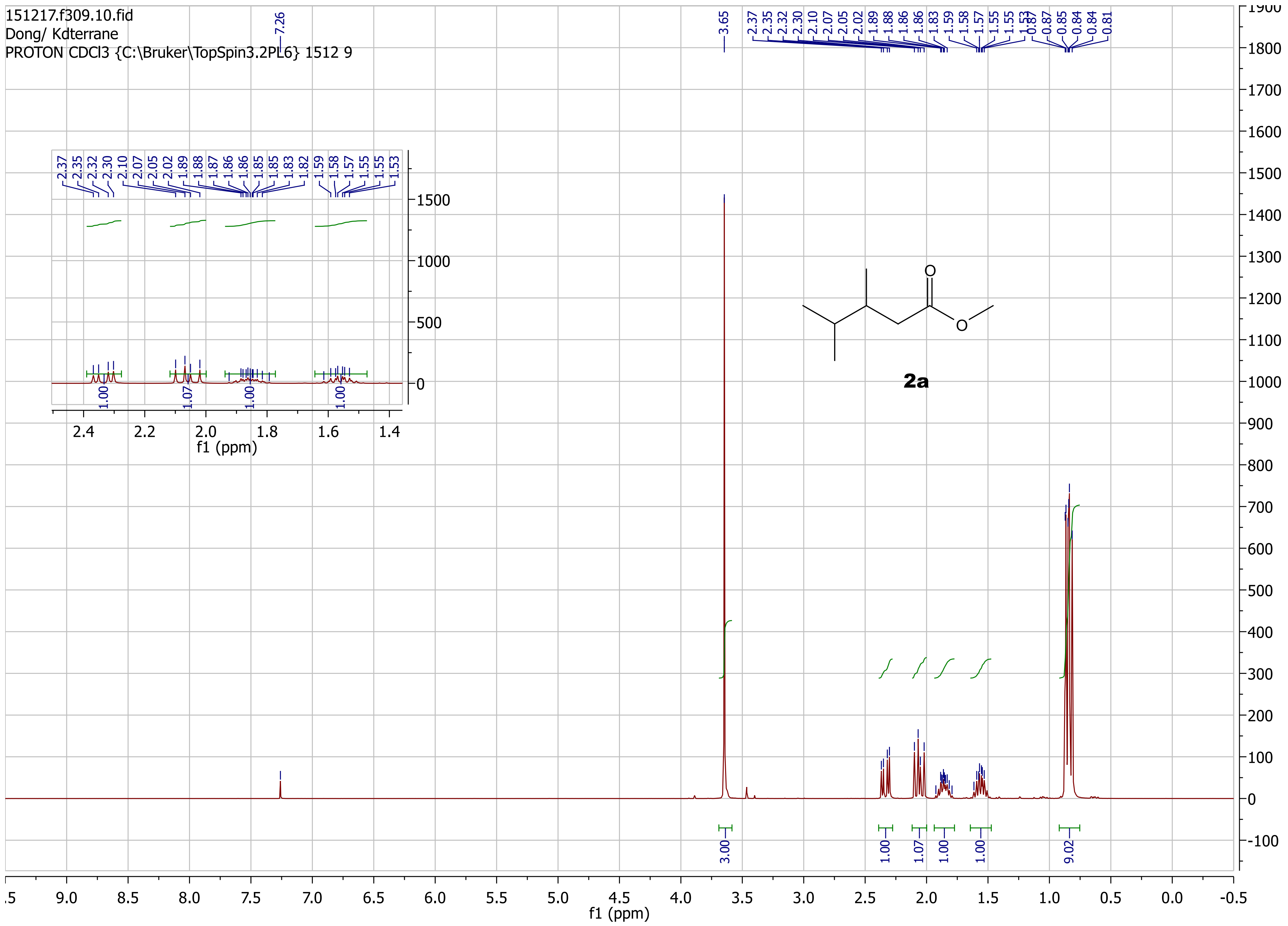
151217.f309.10.fid
Dong/ Kdterrane
PROTON CDCl3 {C:\Bruker\TopSpin3.2PL6} 1512 9
7.26
3.65
2.37 2.35 2.32 2.30 2.10 2.07 2.05 2.02 1.89 1.88 1.86 1.86 1.83 1.59 1.58 1.57 1.55 1.53 0.87 0.87 0.85 0.84 0.84 0.81
2.37 2.35 2.32 2.30 2.10 2.07 2.05 2.02 1.89 1.88 1.87 1.86 1.86 1.85 1.85 1.83 1.82 1.59 1.58 1.57 1.55 1.55 1.53
1.00
1.07
1.00
1.00
f1 (ppm)
O
O
2a
3.00
1.00
1.07
1.00
1.00
9.02
f1 (ppm)

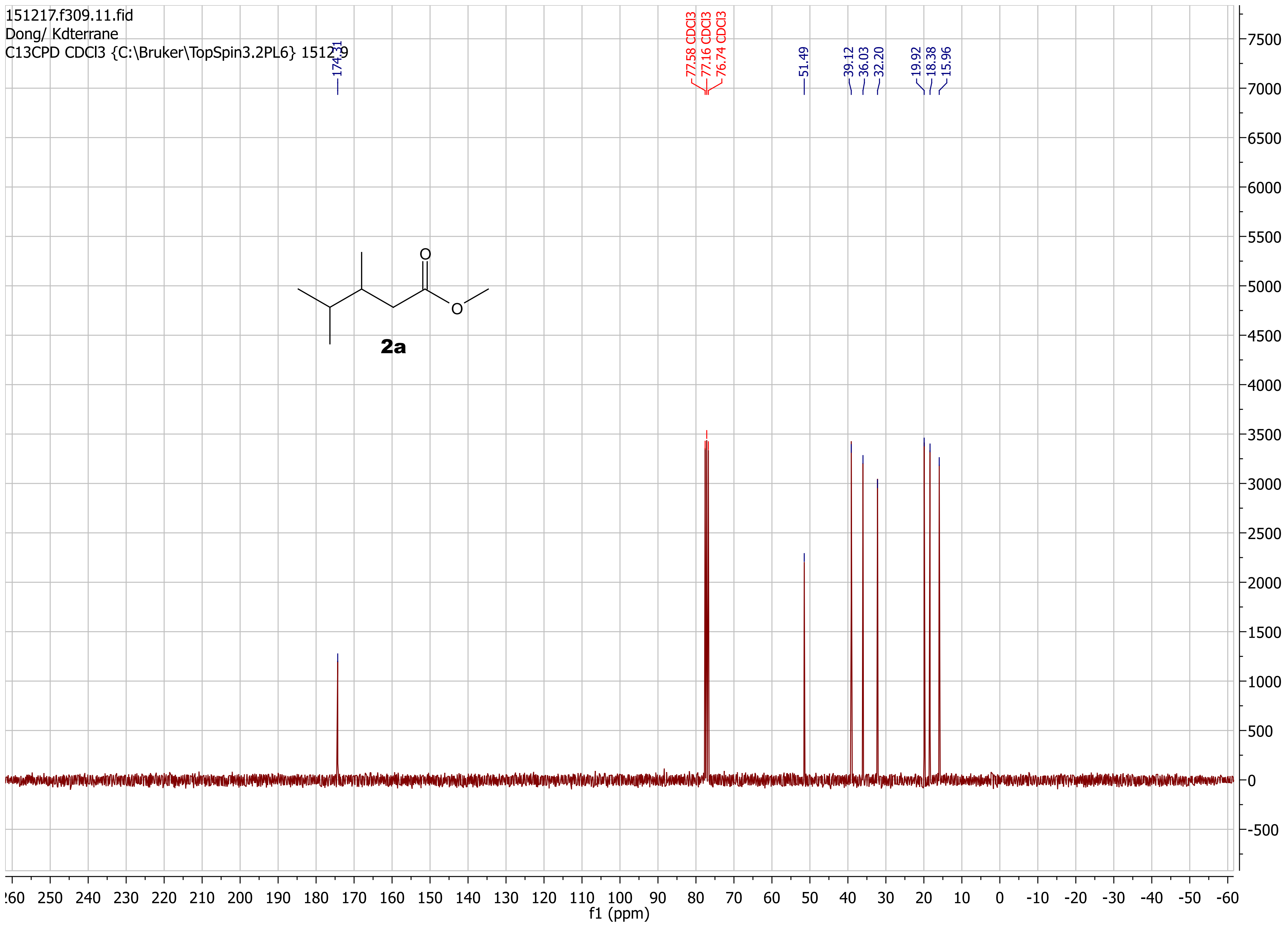
151217.f309.11.fid
Dong/ Kdterrane
C13CPD CDCl3 {C:\Bruker\TopSpin3.2PL6} 151219
174.31
77.58 CDCl3
77.16 CDCl3
76.74 CDCl3
51.49
39.12
36.03
32.20
19.92
18.38
15.96
O
O
2a
f1 (ppm)

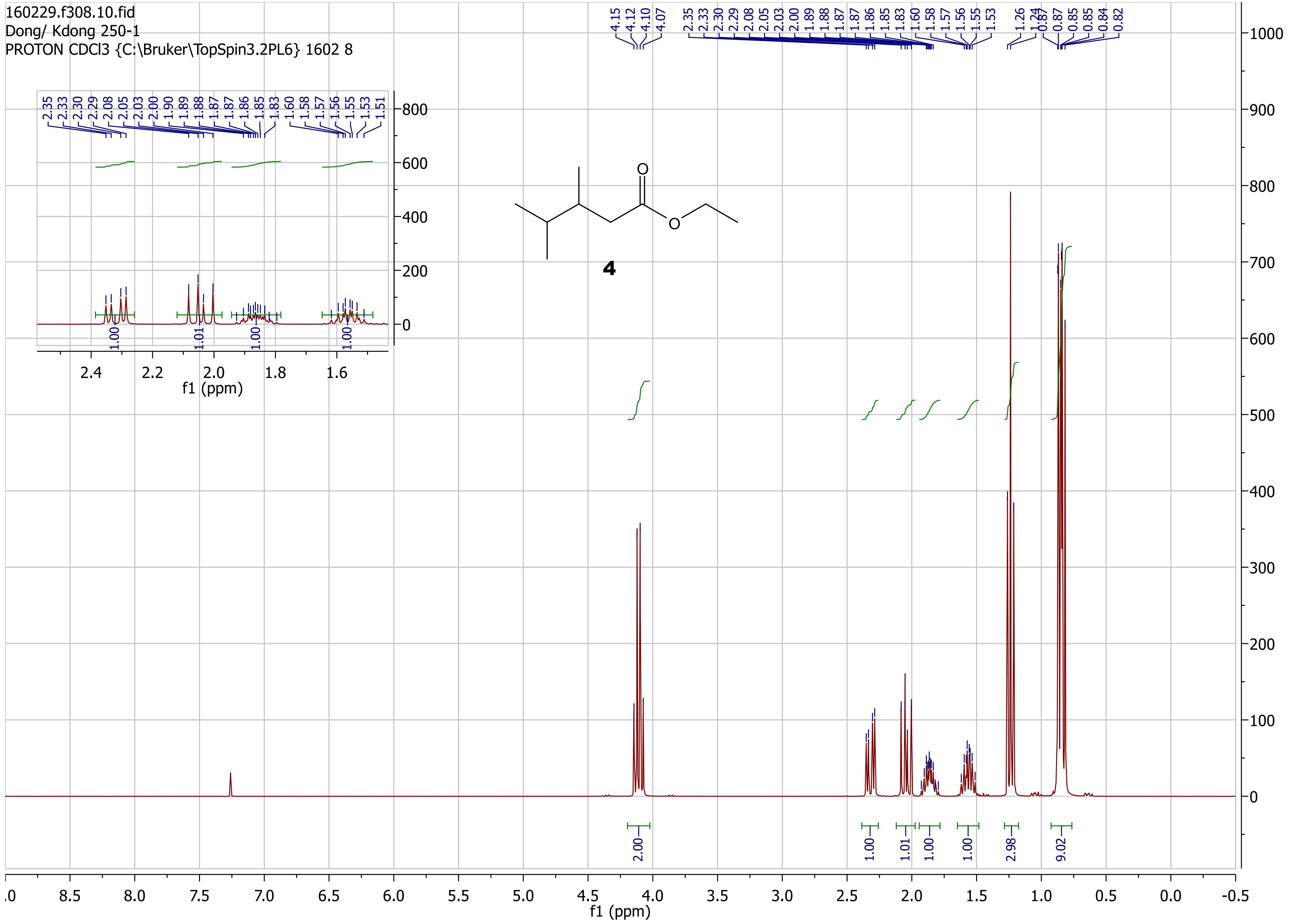
160229.f308.10.fid
Dong/ Kdong 250-1
PROTON CDCl3 {C:\Bruker\TopSpin3.2PL6} 1602 8
4
O
O
f1 (ppm)
f1 (ppm)
2.00
1.00
1.01
1.00
1.00
2.98
9.02

160229.f308.11.fid
Dong/ Kdong 250-1
C13CPD CDCl3 {C:\Bruker\TopSpin3.2PL6} 160228

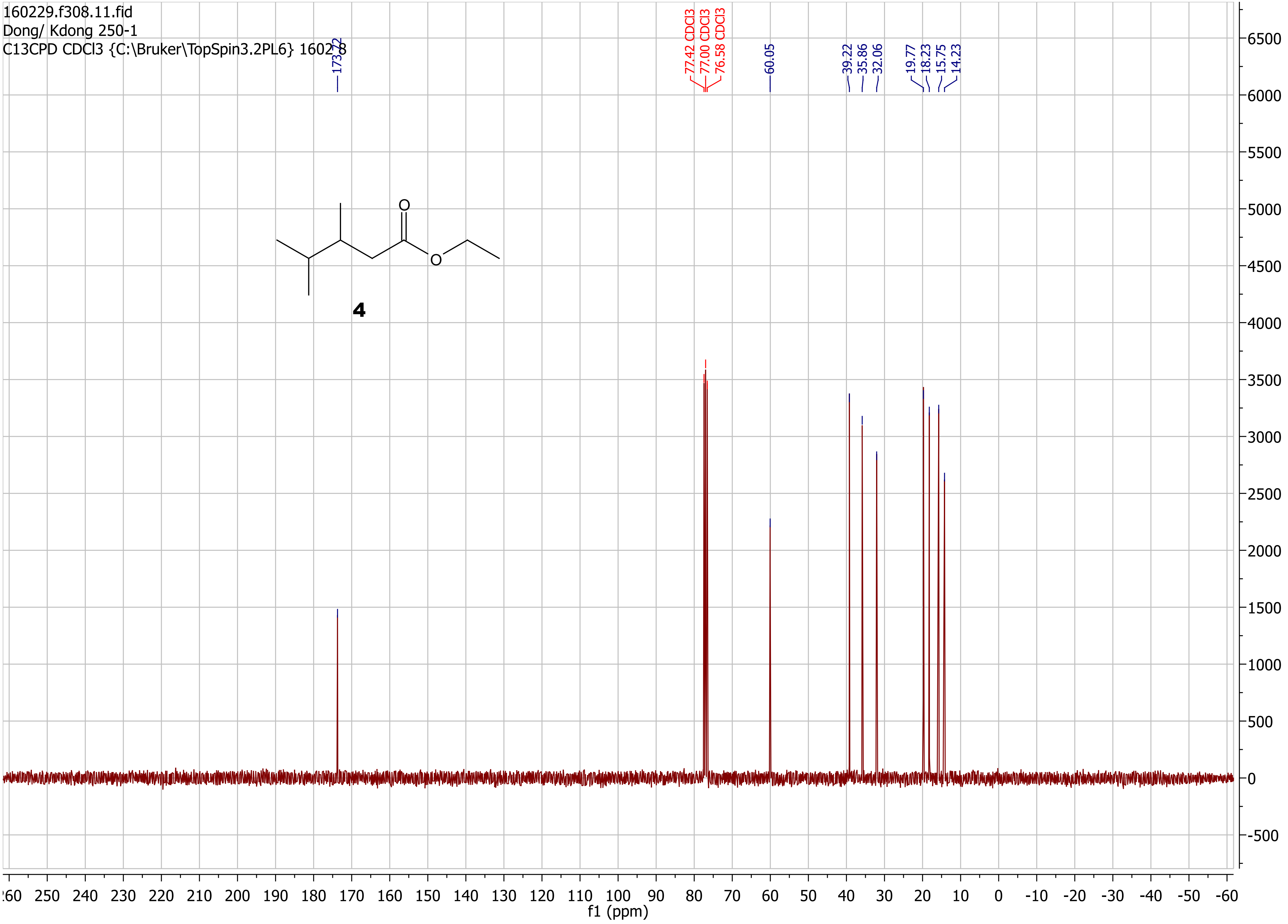

160115.f301.10.fid
Kaiwu Dong kd250-2
PROTON CDCl3 {C:\Bruker\TopSpin3.2PL6} 1601 1

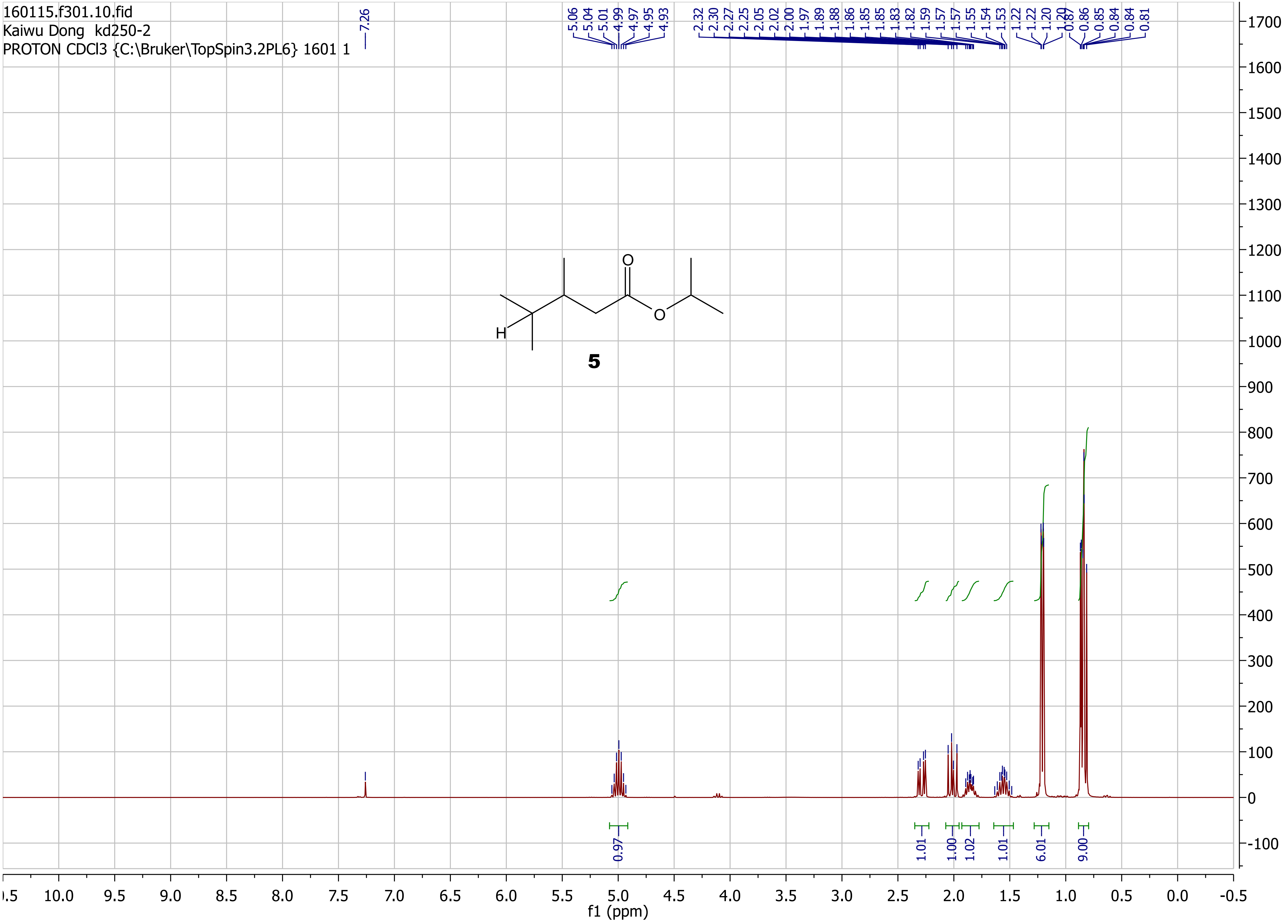

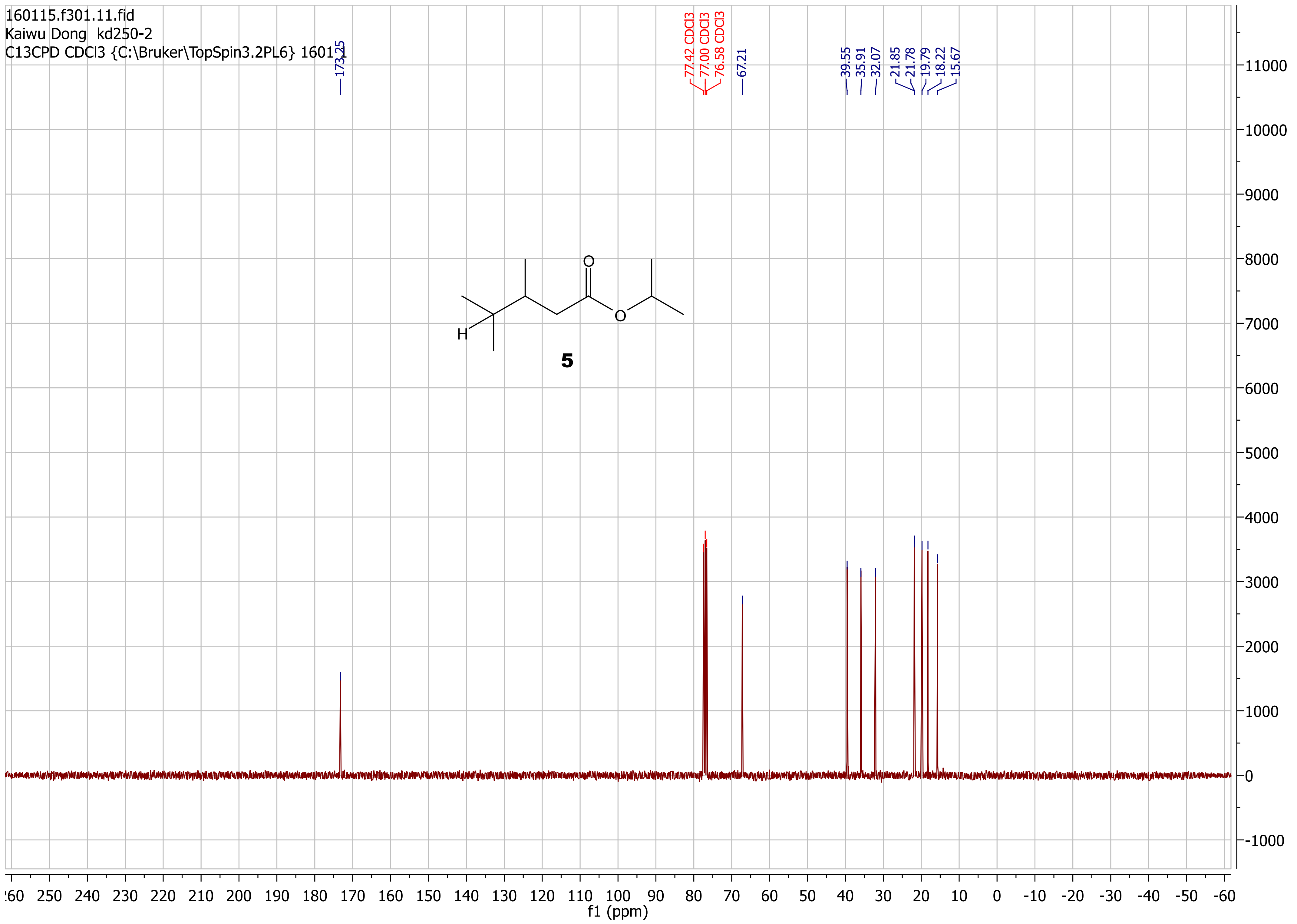

160115.f301.11.fid
Kaiwu Dong kd250-2
C13CPD CDCl3 {C:\Bruker\TopSpin3.2PL6} 16011
173.25
77.42 CDCl3
77.00 CDCl3
76.58 CDCl3
67.21
39.55
35.91
32.07
21.85
21.78
19.79
18.22
15.67
O
O
H
5
f1 (ppm)

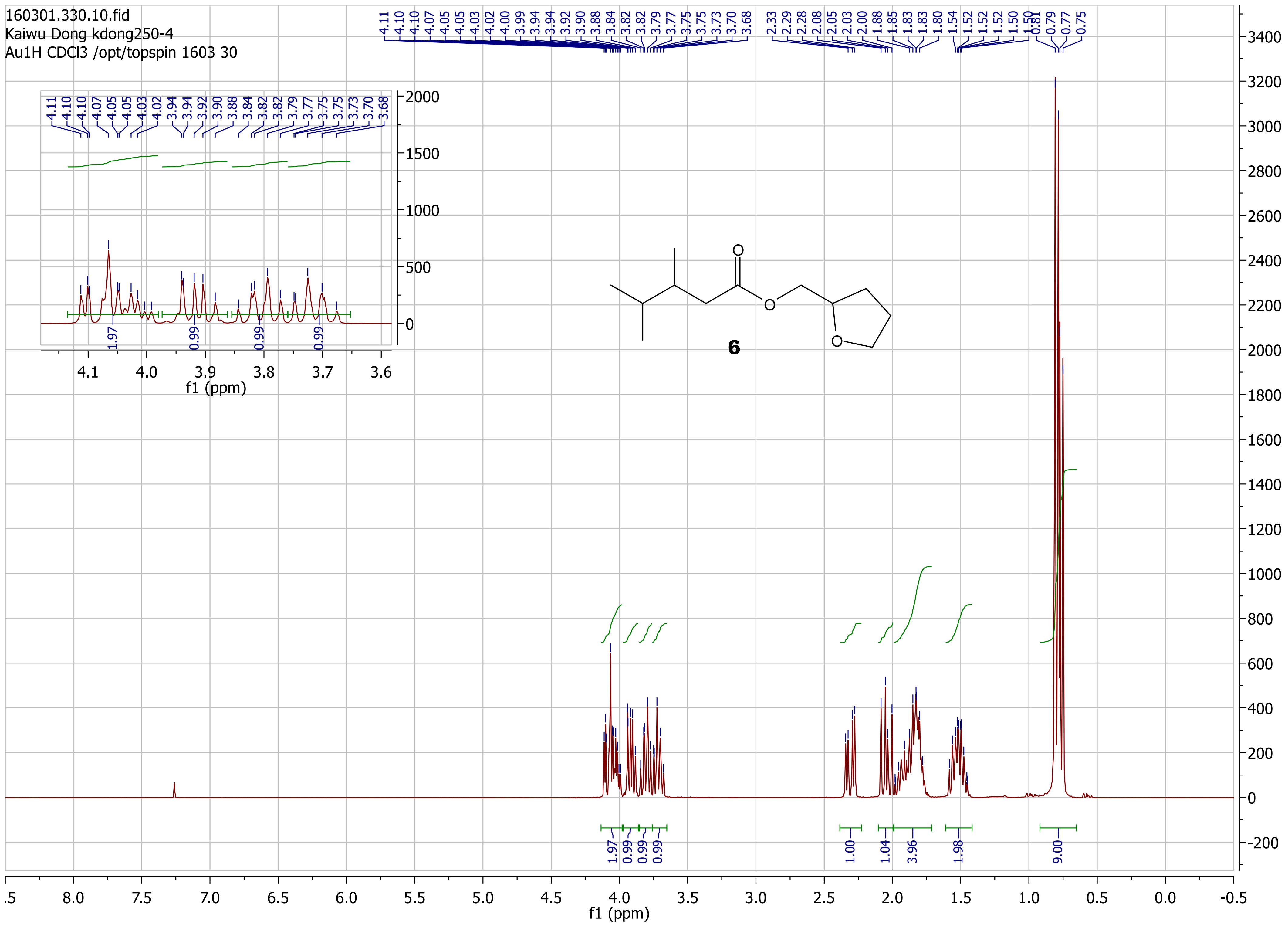
160301.330.10.fid
Kaiwu Dong kdong250-4
Au1H CDCl3 /opt/topspin 1603 30
4.11 4.10 4.10 4.07 4.05 4.05 4.03 4.02 4.00 3.99 3.94 3.94 3.92 3.90 3.88 3.84 3.82 3.82 3.79 3.77 3.75 3.75 3.73 3.70 3.68
2.33 2.29 2.28 2.08 2.05 2.03 2.00 1.88 1.85 1.83 1.83 1.80 1.54 1.52 1.52 1.52 1.50 0.81 0.79 0.77 0.75
1.97 0.99 0.99 0.99 1.00 1.04 3.96 1.98 9.00
O
O
O
6
f1 (ppm)
f1 (ppm)

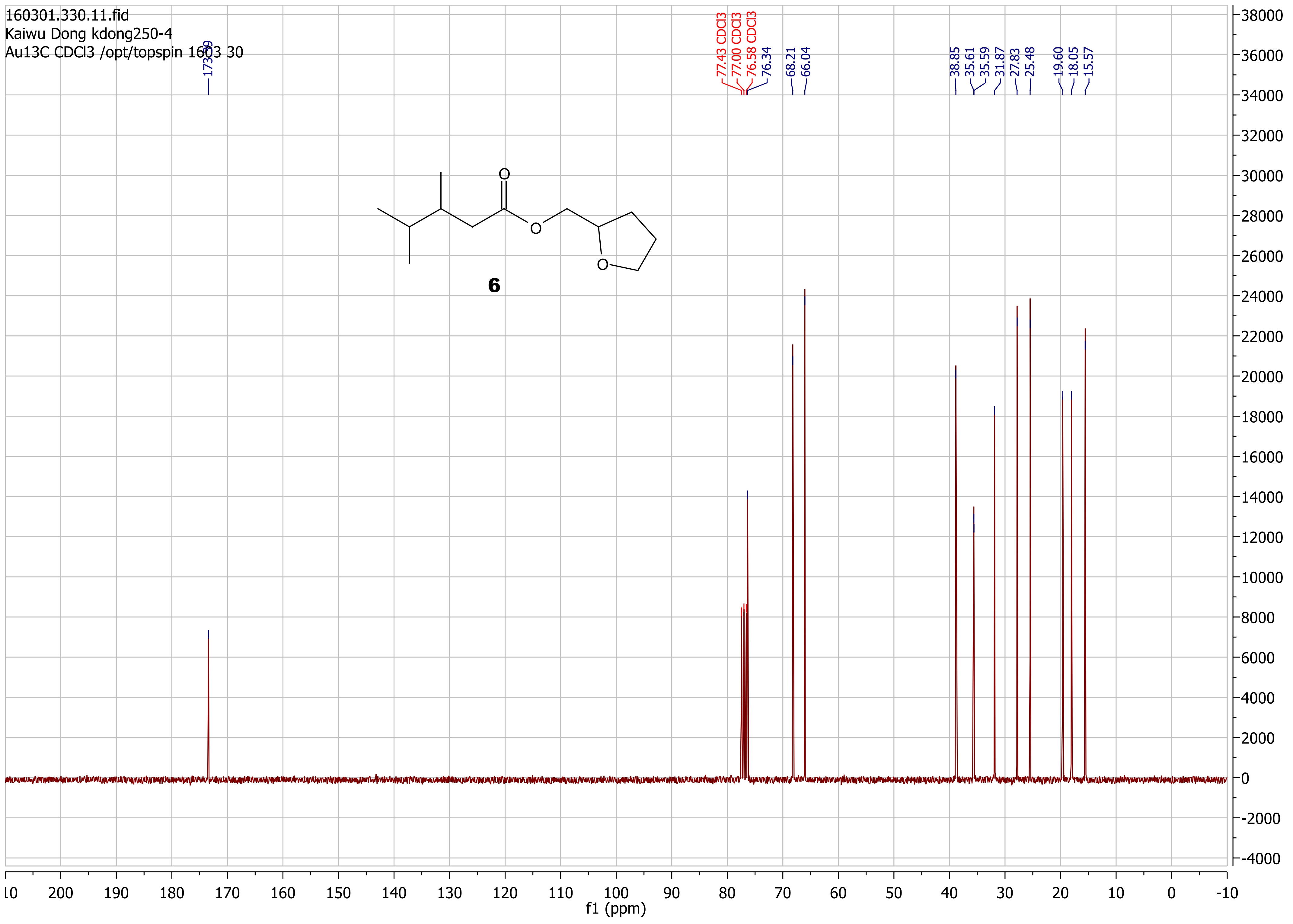
160301.330.11.fid
Kaiwu Dong kdong250-4
Au13C CDCl3 /opt/topspin 1603 30
173.39
77.43 CDCl3
77.00 CDCl3
76.58 CDCl3
76.34
68.21
66.04
38.85
35.61
35.59
31.87
27.83
25.48
19.60
18.05
15.57
O
O
O
6
f1 (ppm)

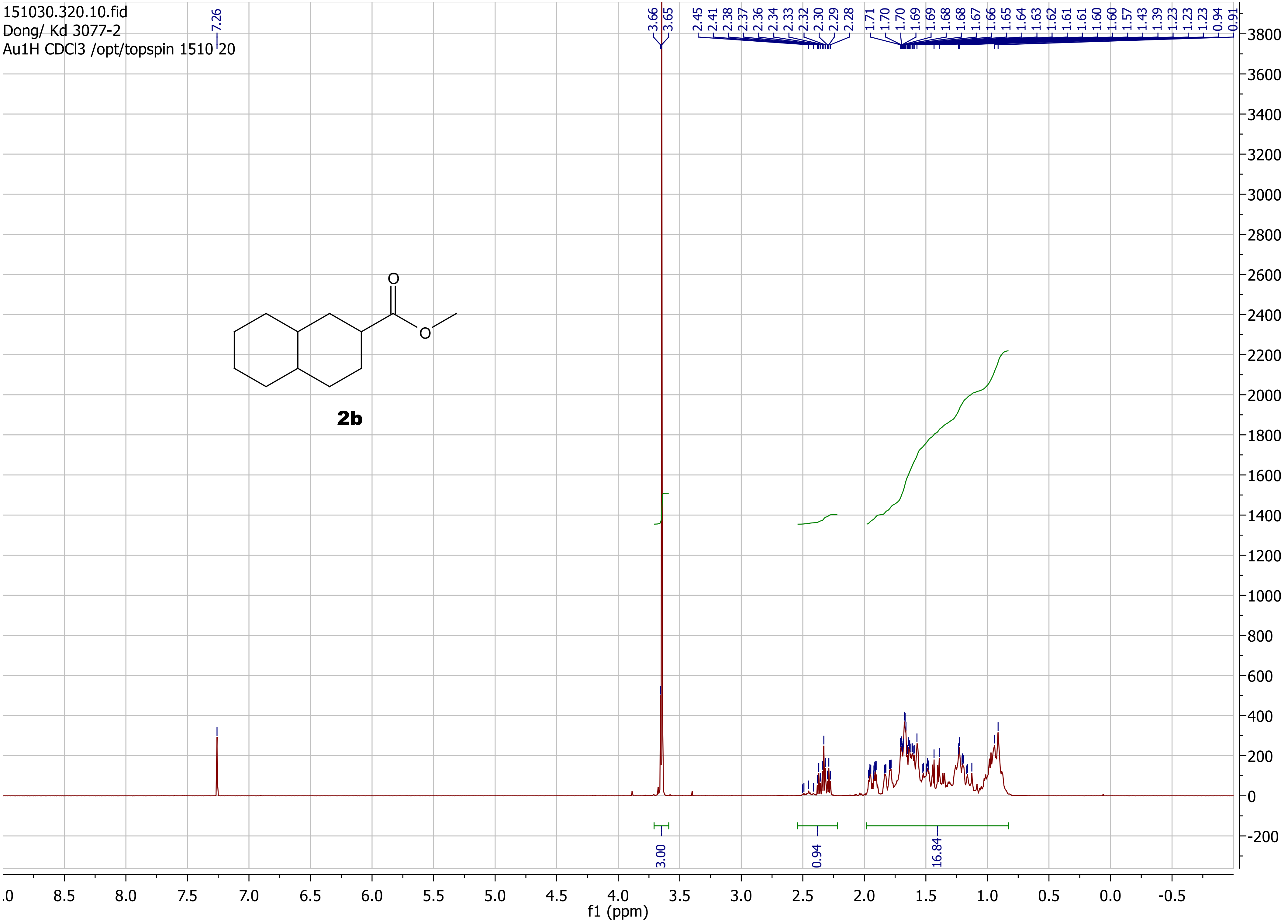
2b
151030.320.10.fid
Dong/ Kd 3077-2
Au1H CDCl3 /opt/topspin 1510 20
7.26
3.66
3.65
2.45
2.41
2.38
2.37
2.36
2.34
2.33
2.32
2.30
2.29
2.28
1.71
1.70
1.70
1.69
1.69
1.68
1.68
1.67
1.66
1.65
1.64
1.63
1.62
1.61
1.61
1.60
1.57
1.43
1.39
1.23
1.23
1.23
0.94
0.91
3.00
0.94
16.84
f1 (ppm)

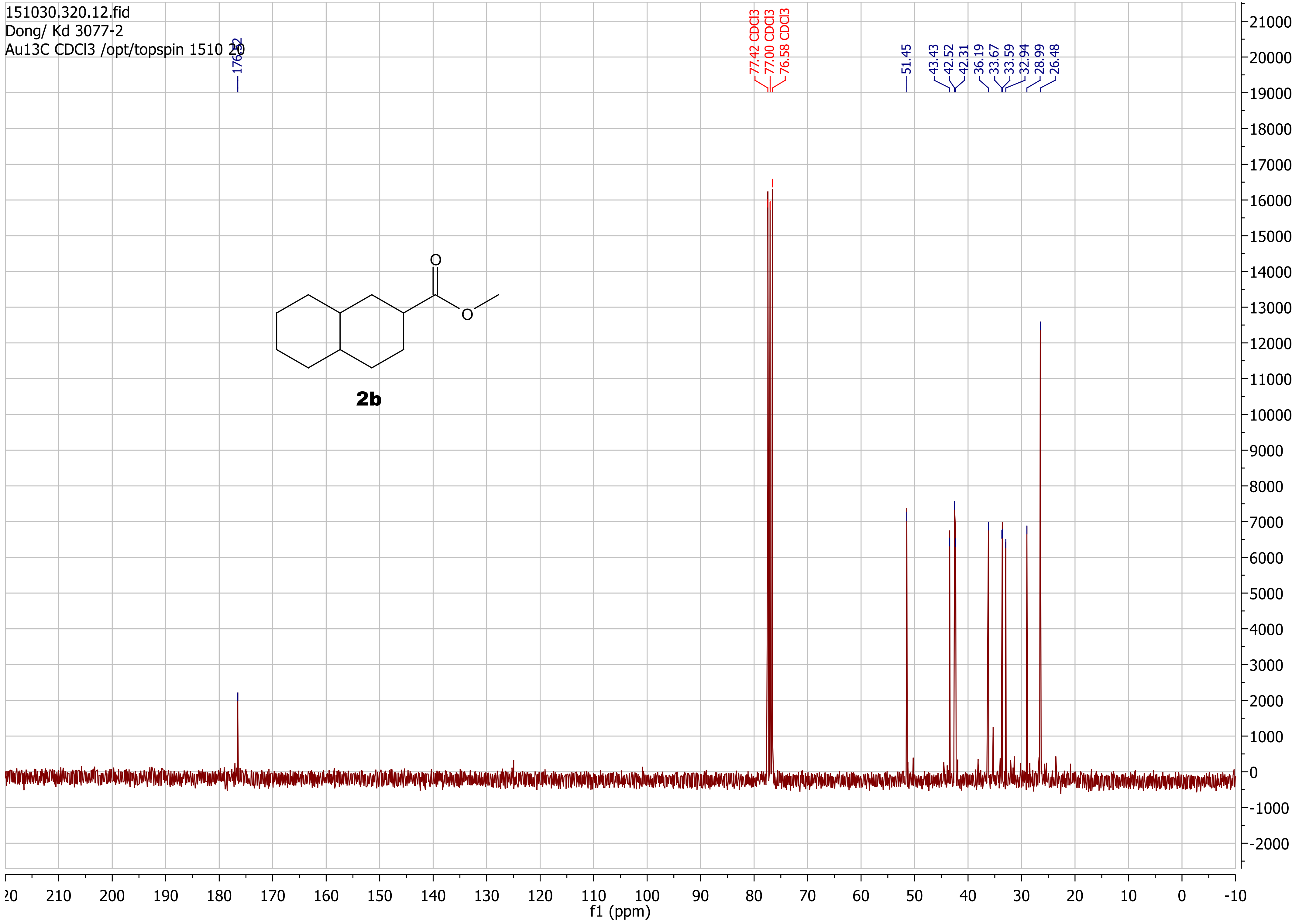

151030.320.12.fid
Dong/ Kd 3077-2
Au13C CDCl3 /opt/topspin 1510 20
176.52
77.42 CDCl3
77.00 CDCl3
76.58 CDCl3
51.45
43.43
42.52
42.31
36.19
33.67
33.59
32.94
28.99
26.48
O
O
2b
f1 (ppm)

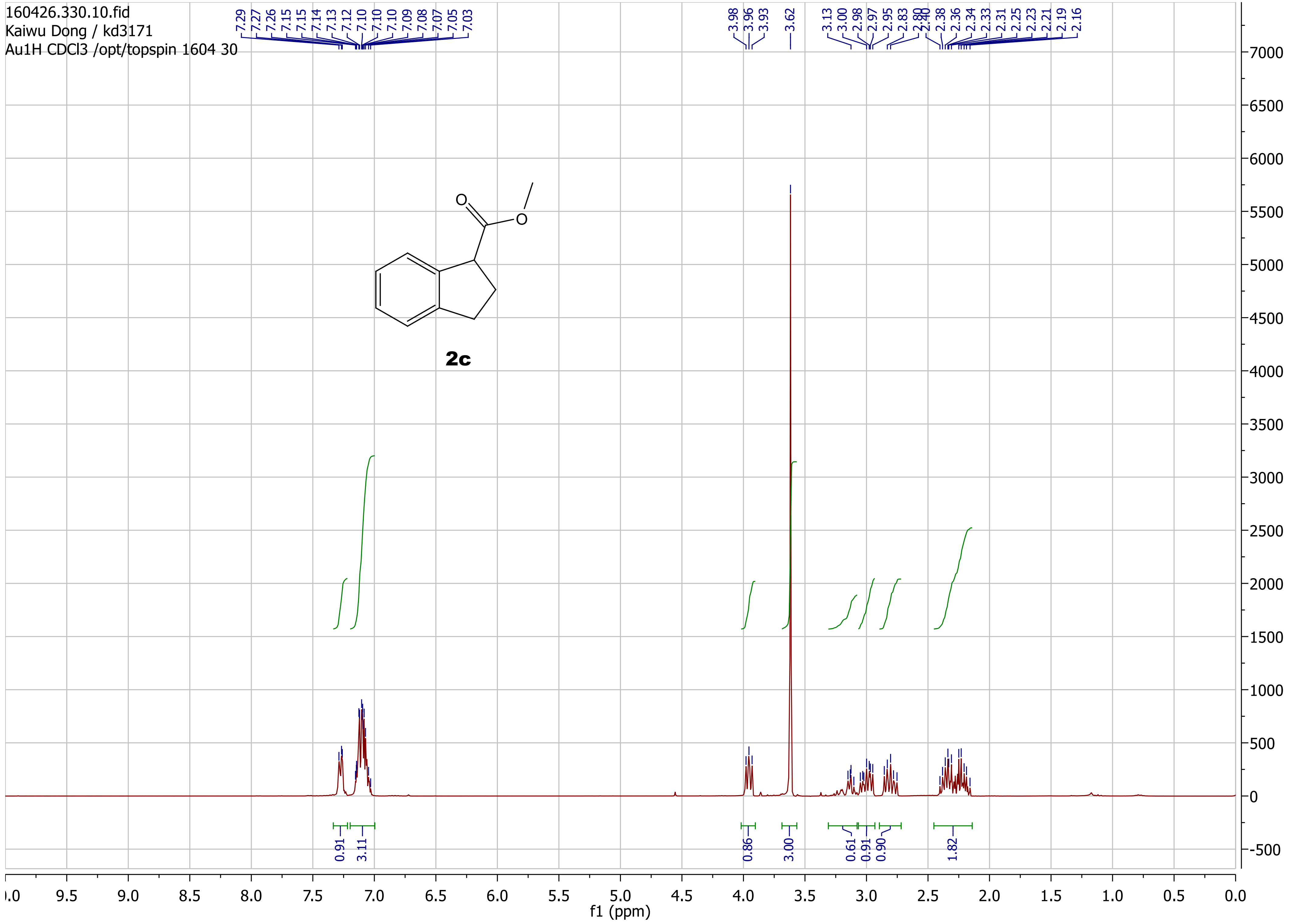
160426.330.10.fid
Kaiwu Dong / kd3171
Au1H CDCl3 /opt/topspin 1604 30
7.29
7.27
7.26
7.15
7.15
7.14
7.13
7.12
7.10
7.10
7.10
7.09
7.08
7.07
7.05
7.03
3.98
3.96
3.93
3.62
3.13
3.00
2.98
2.97
2.95
2.83
2.80
2.40
2.38
2.36
2.34
2.33
2.31
2.25
2.23
2.21
2.19
2.16
O
O
2c
0.91
3.11
0.86
3.00
0.61
0.91
0.90
1.82
f1 (ppm)

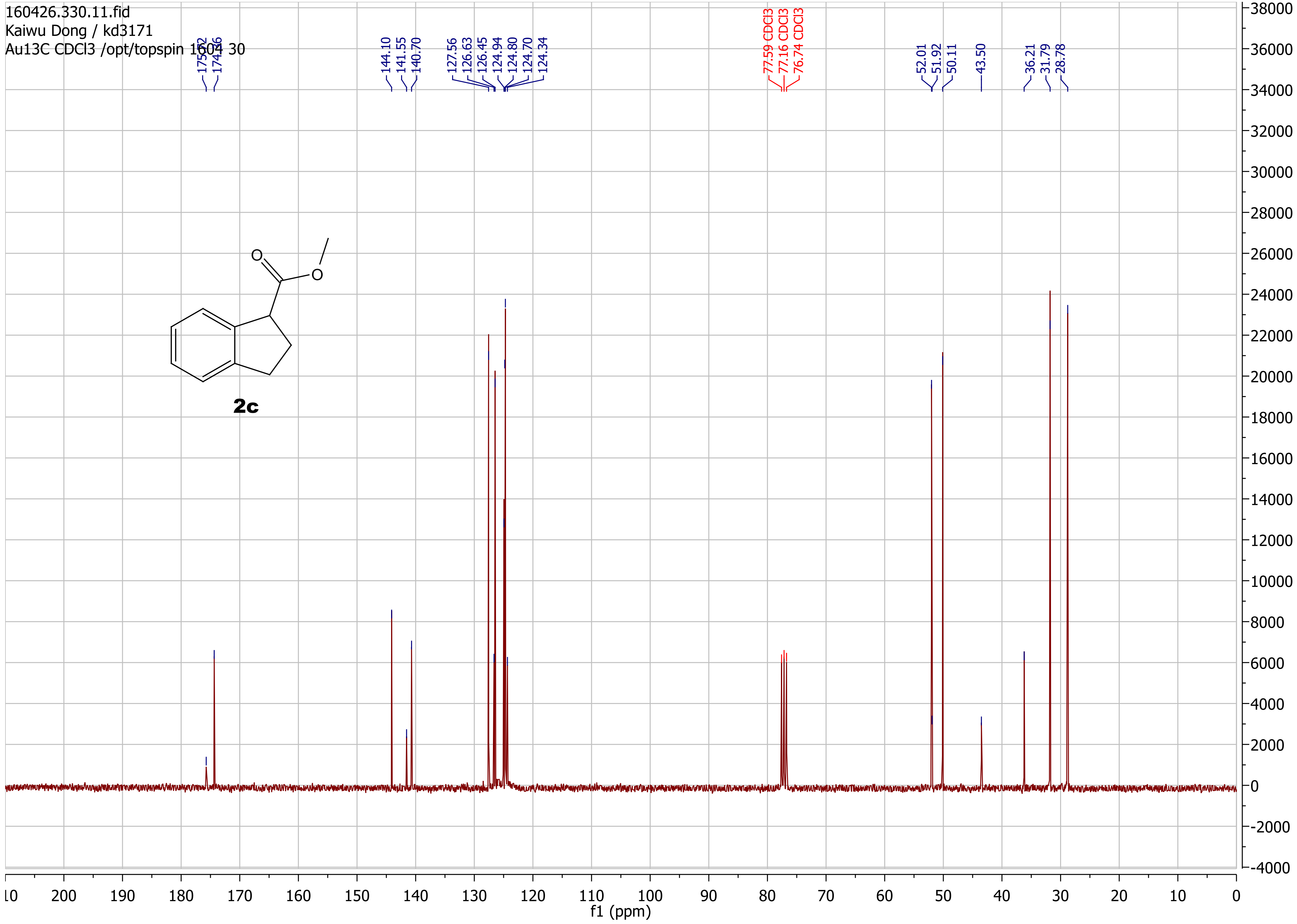

160426.330.11.fid
Kaiwu Dong / kd3171
Au13C CDCl3 /opt/topspin 1604 30
175.72
174.36
144.10
141.55
140.70
127.56
126.63
126.45
124.94
124.80
124.70
124.34
77.59 CDCl3
77.16 CDCl3
76.74 CDCl3
52.01
51.92
50.11
43.50
36.21
31.79
28.78
O
O
2c
f1 (ppm)

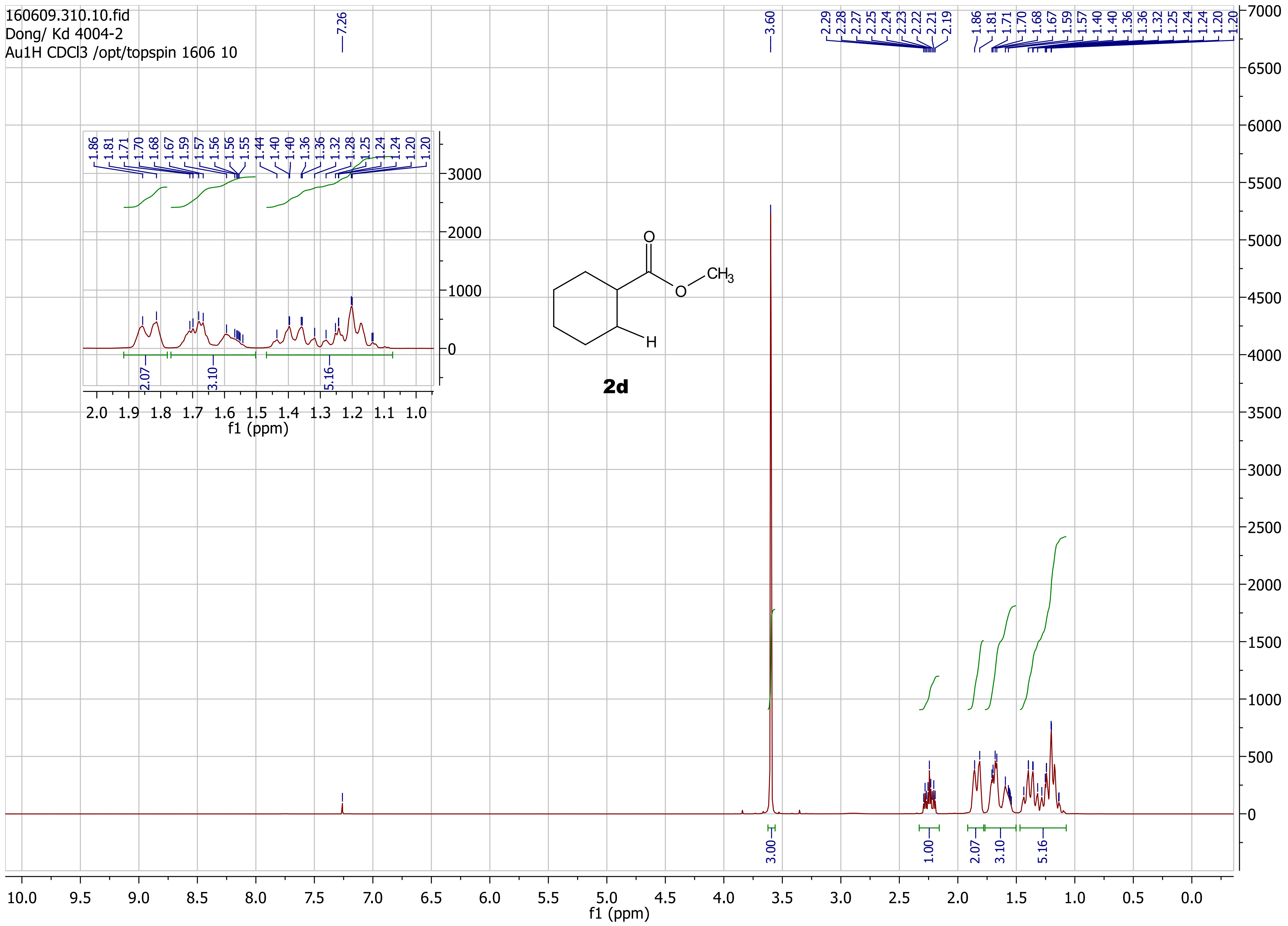
160609.310.10.fid
Dong/ Kd 4004-2
Au1H CDCl3 /opt/topspin 1606 10
7.26
3.60
2.29 2.28 2.27 2.25 2.24 2.23 2.22 2.21 2.19
1.86 1.81 1.71 1.70 1.68 1.67 1.59 1.57 1.40 1.40 1.36 1.32 1.25 1.24 1.24 1.20 1.20
3.00
1.00
2.07
3.10
5.16
f1 (ppm)
O
CH3
O
H
2d

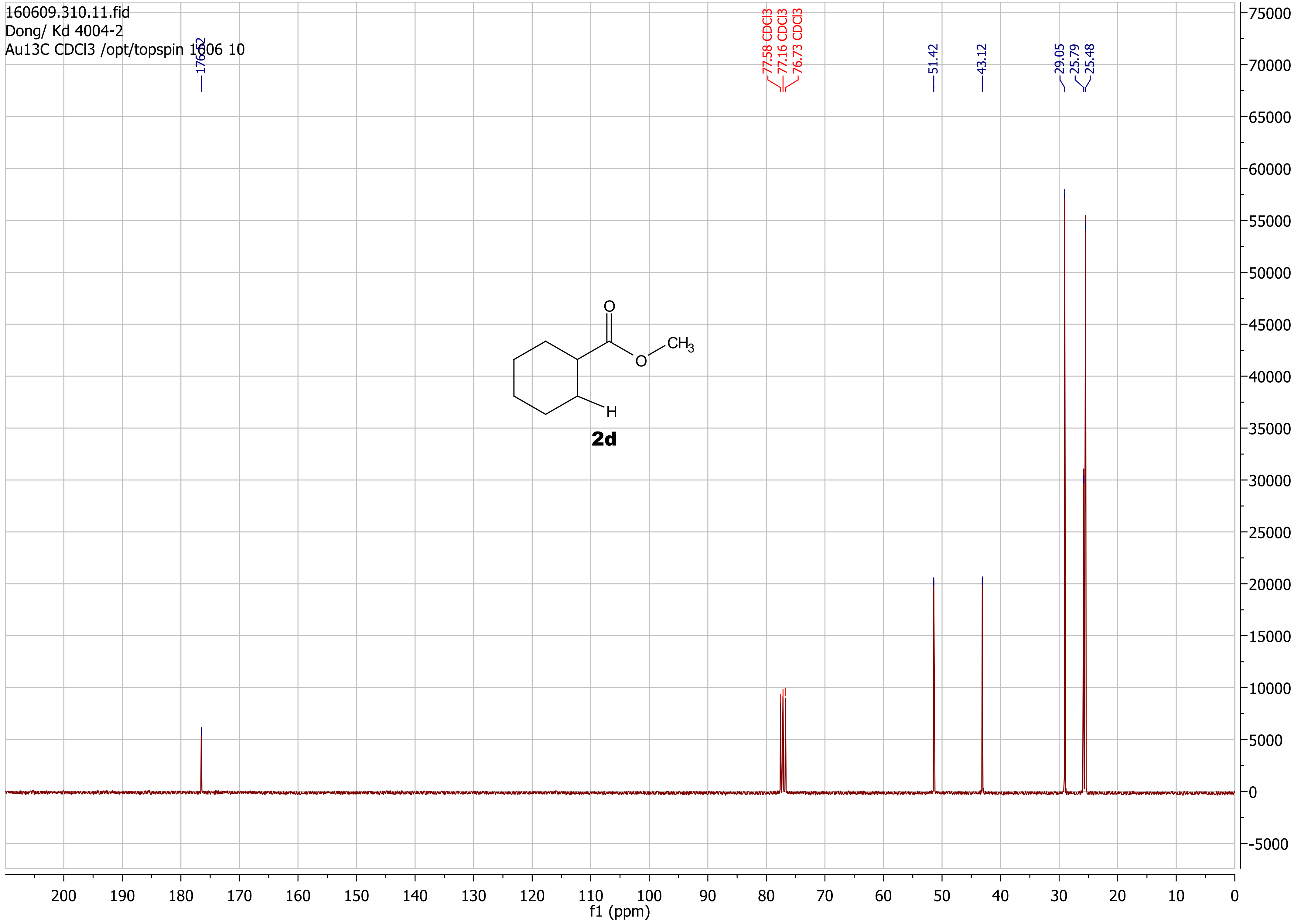

160609.310.11.fid
Dong/ Kd 4004-2
Au13C CDCl3 /opt/topspin 1606 10
176.52
77.58 CDCl3
77.16 CDCl3
76.73 CDCl3
51.42
43.12
29.05
25.79
25.48
O
O
CH3
H
2d
f1 (ppm)

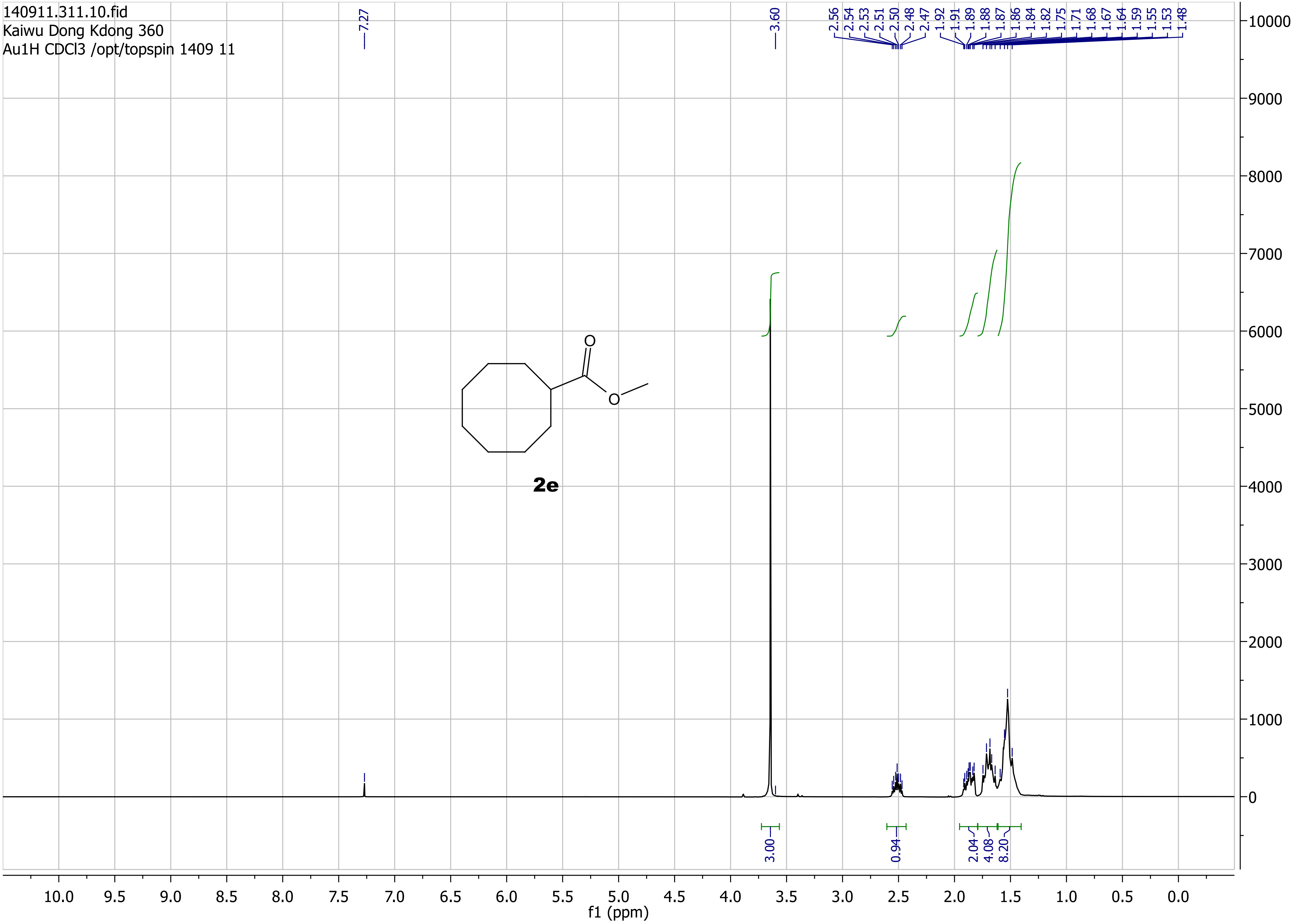

140911.311.10.fid
Kaiwu Dong Kdong 360
Au1H CDCl3 /opt/topspin 1409 11
7.27
3.60
2.56 2.54 2.53 2.51 2.50 2.48 2.47
1.92 1.91 1.89 1.88 1.87 1.86 1.84 1.82 1.75 1.71 1.68 1.67 1.64 1.59 1.55 1.53 1.48
O
O
2e
3.00
0.94
2.04
4.08
8.20
f1 (ppm)

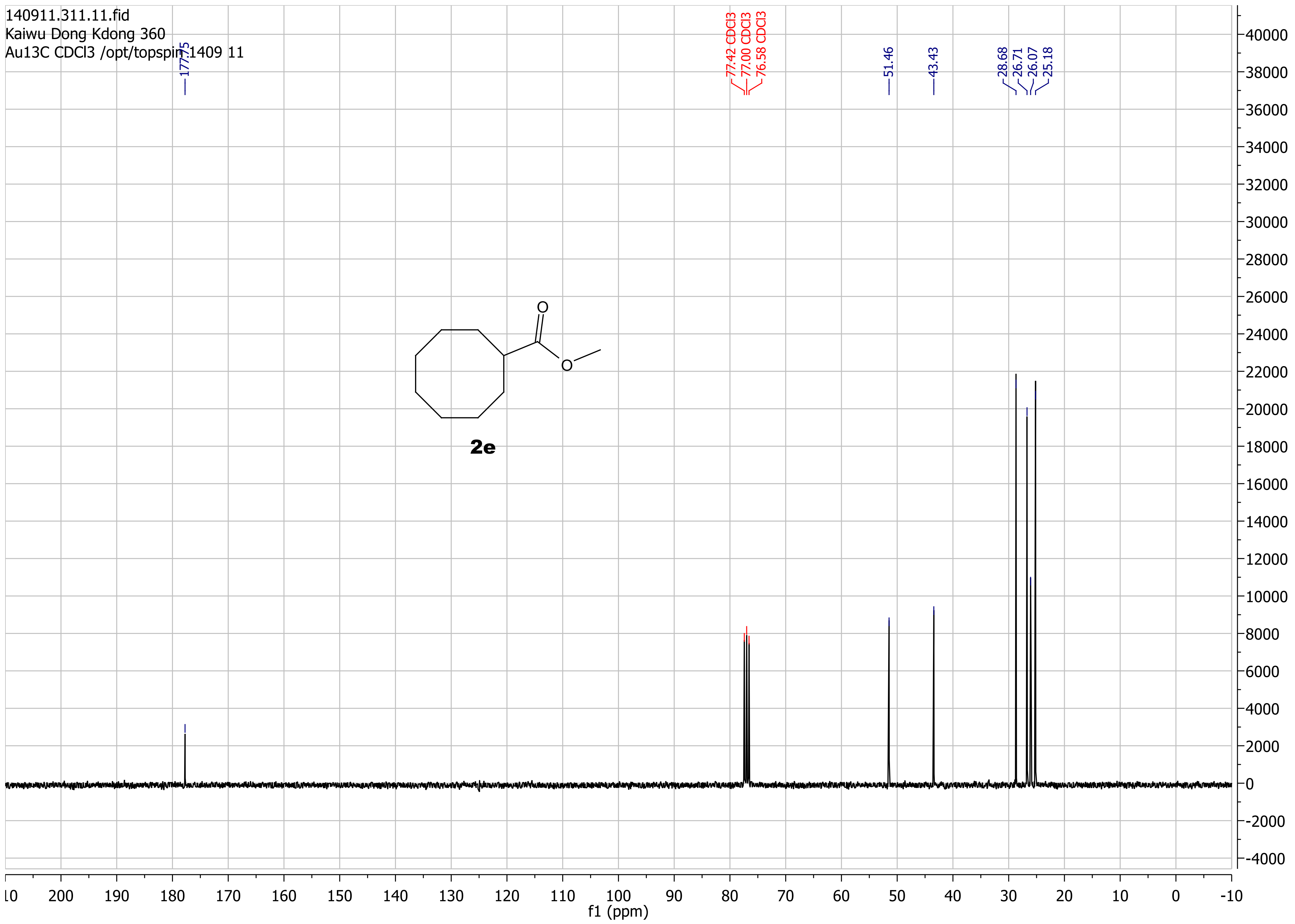

140911.311.11.fid
Kaiwu Dong Kdong 360
Au13C CDCl3 /opt/topspin 1409 11
177.75
77.42 CDCl3
77.00 CDCl3
76.58 CDCl3
51.46
43.43
28.68
26.71
26.07
25.18
O
O
2e
f1 (ppm)

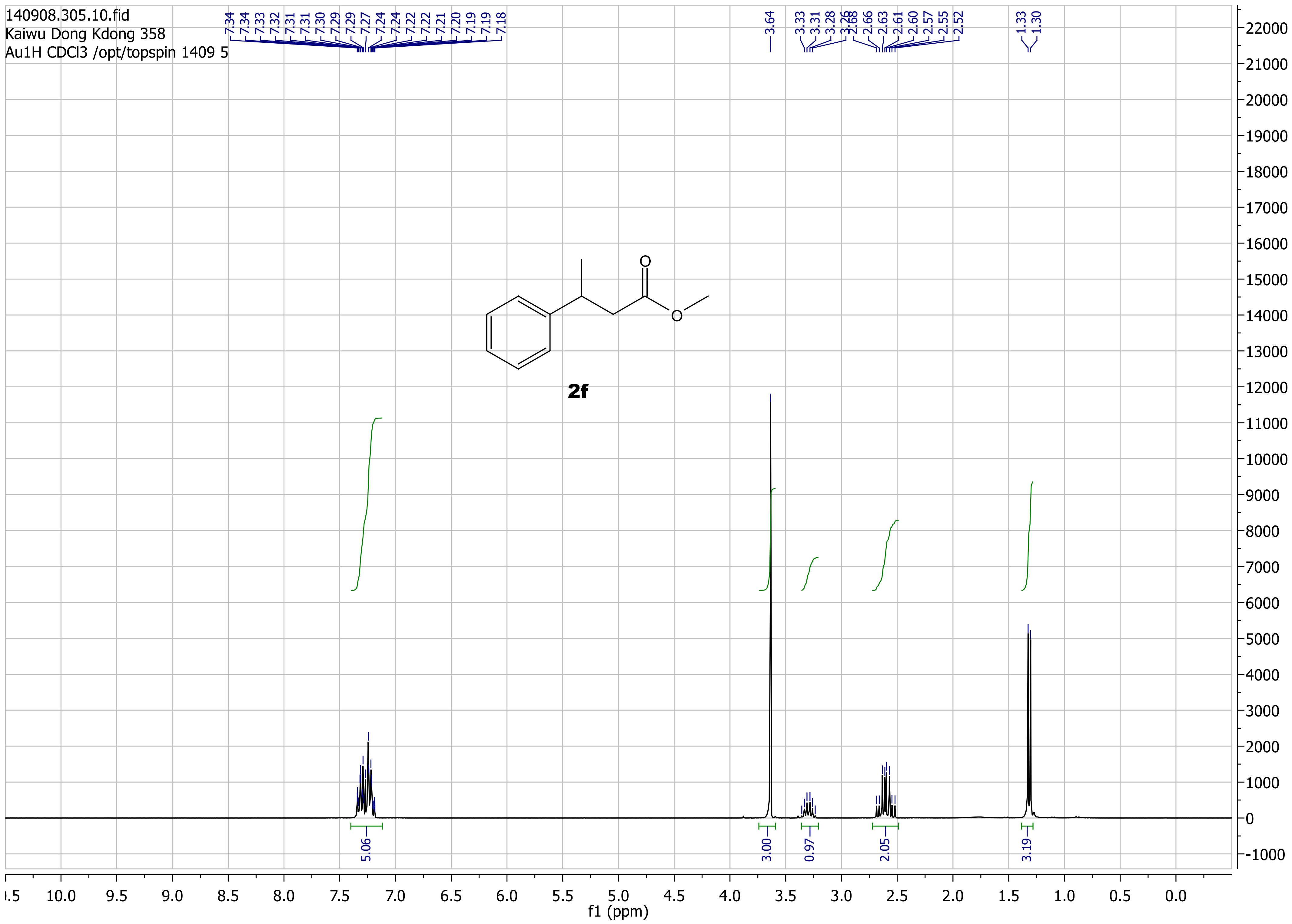
140908.305.10.fid
Kaiwu Dong Kdong 358
Au1H CDCl3 /opt/topspin 1409 5
7.34 7.34 7.33 7.32 7.31 7.31 7.30 7.29 7.29 7.27 7.24 7.24 7.22 7.22 7.21 7.20 7.19 7.19 7.18
3.64
3.33 3.31 3.28 3.26 2.68 2.66 2.63 2.61 2.60 2.57 2.55 2.52
1.33 1.30
O
O
2f
5.06
3.00
0.97
2.05
3.19
f1 (ppm)

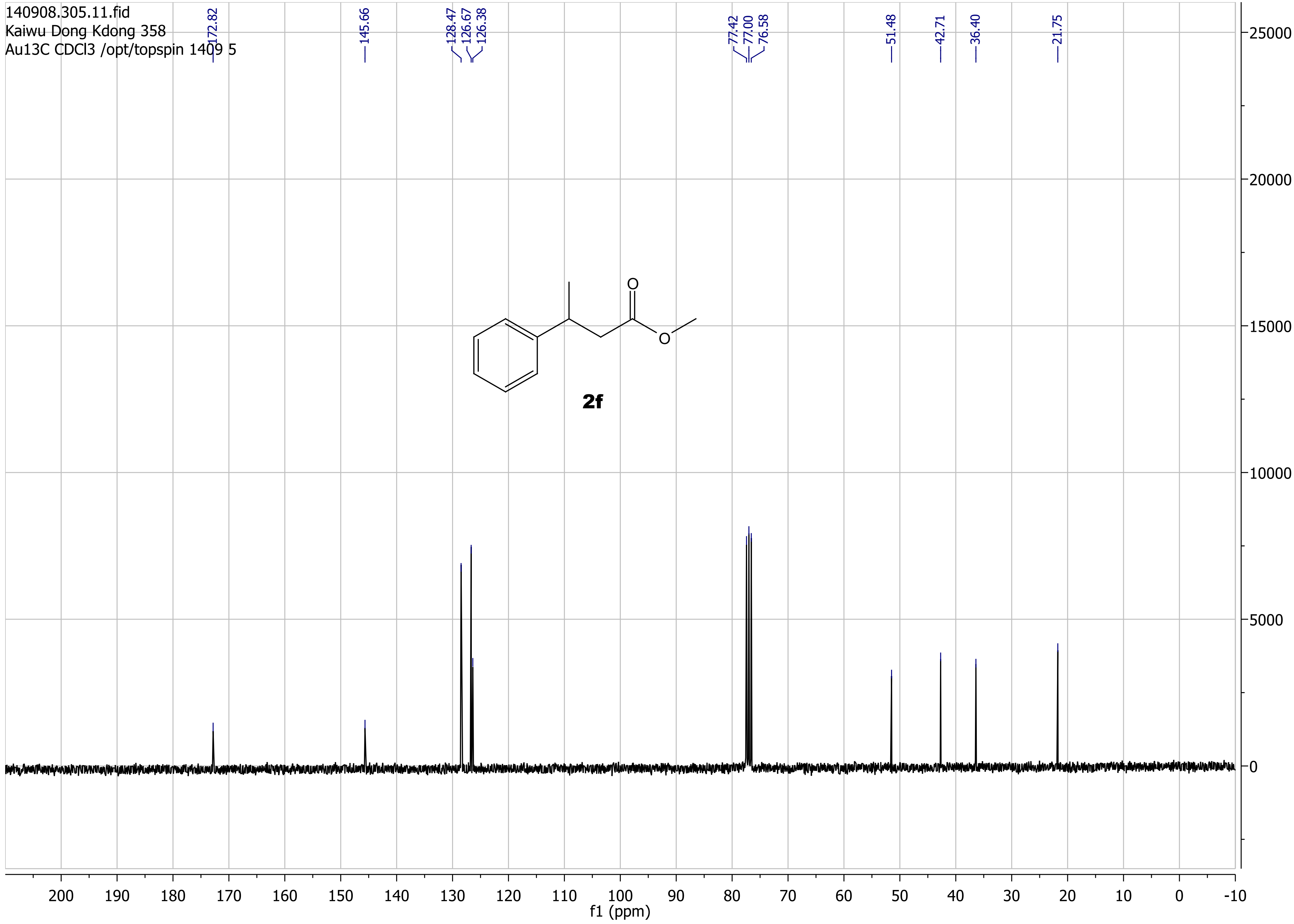
140908.305.11.fid
Kaiwu Dong Kdong 358
Au13C CDCl3 /opt/topspin 1409 5
172.82
145.66
128.47
126.67
126.38
77.42
77.00
76.58
51.48
42.71
36.40
21.75
O
O
2f
25000
20000
15000
10000
5000
0
200
190
180
170
160
150
140
130
120
110
100
90
80
70
60
50
40
30
20
10
0
-10
f1 (ppm)

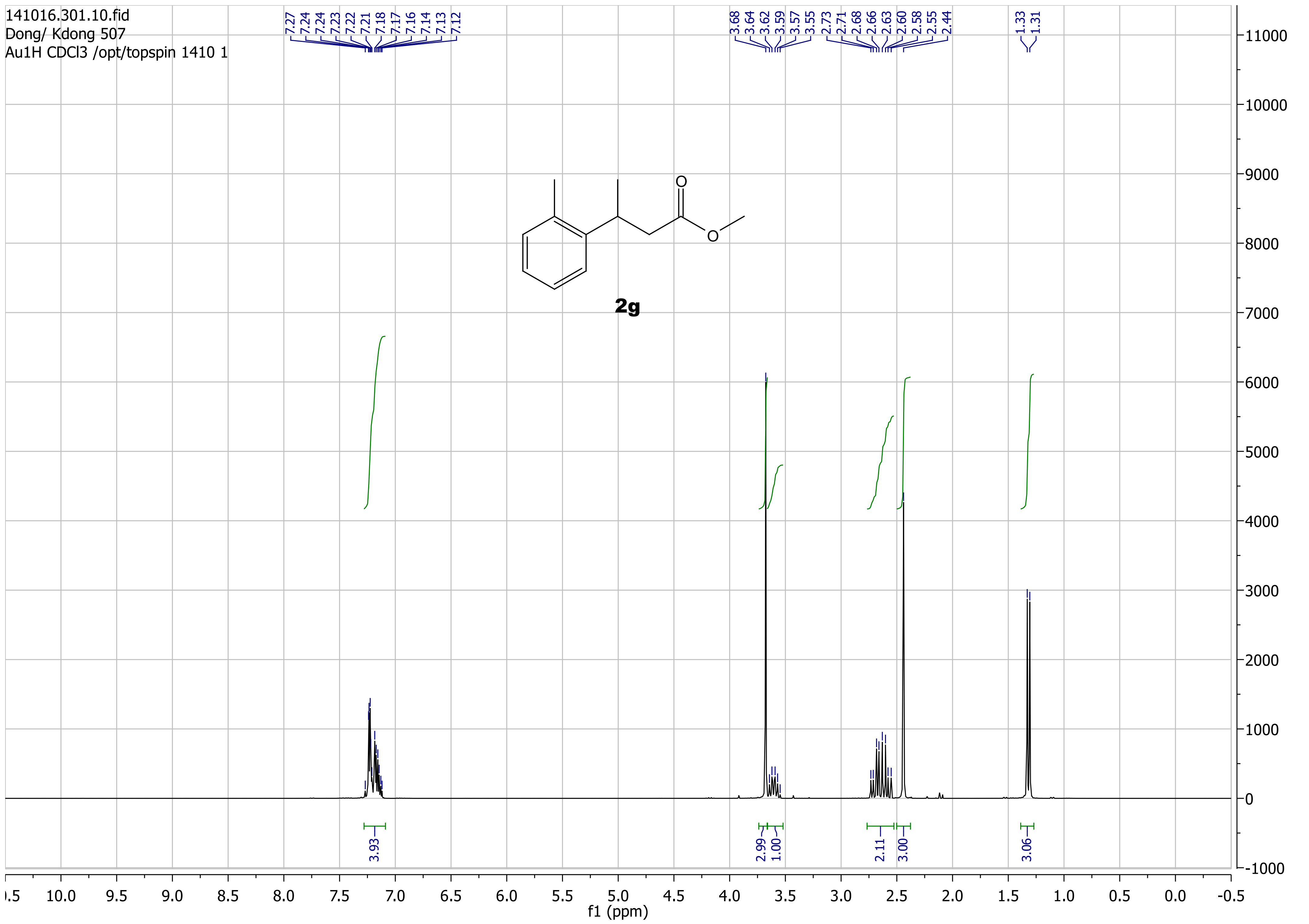

141016.301.10.fid
Dong/ Kdong 507
Au1H CDCl3 /opt/topspin 1410 1
7.27 7.24 7.24 7.23 7.22 7.21 7.18 7.17 7.16 7.14 7.13 7.12
3.68 3.64 3.62 3.59 3.57 3.55 2.73 2.71 2.68 2.66 2.63 2.60 2.58 2.55 2.44
1.33 1.31
O
O
2g
3.93
2.99
1.00
2.11
3.00
3.06
f1 (ppm)

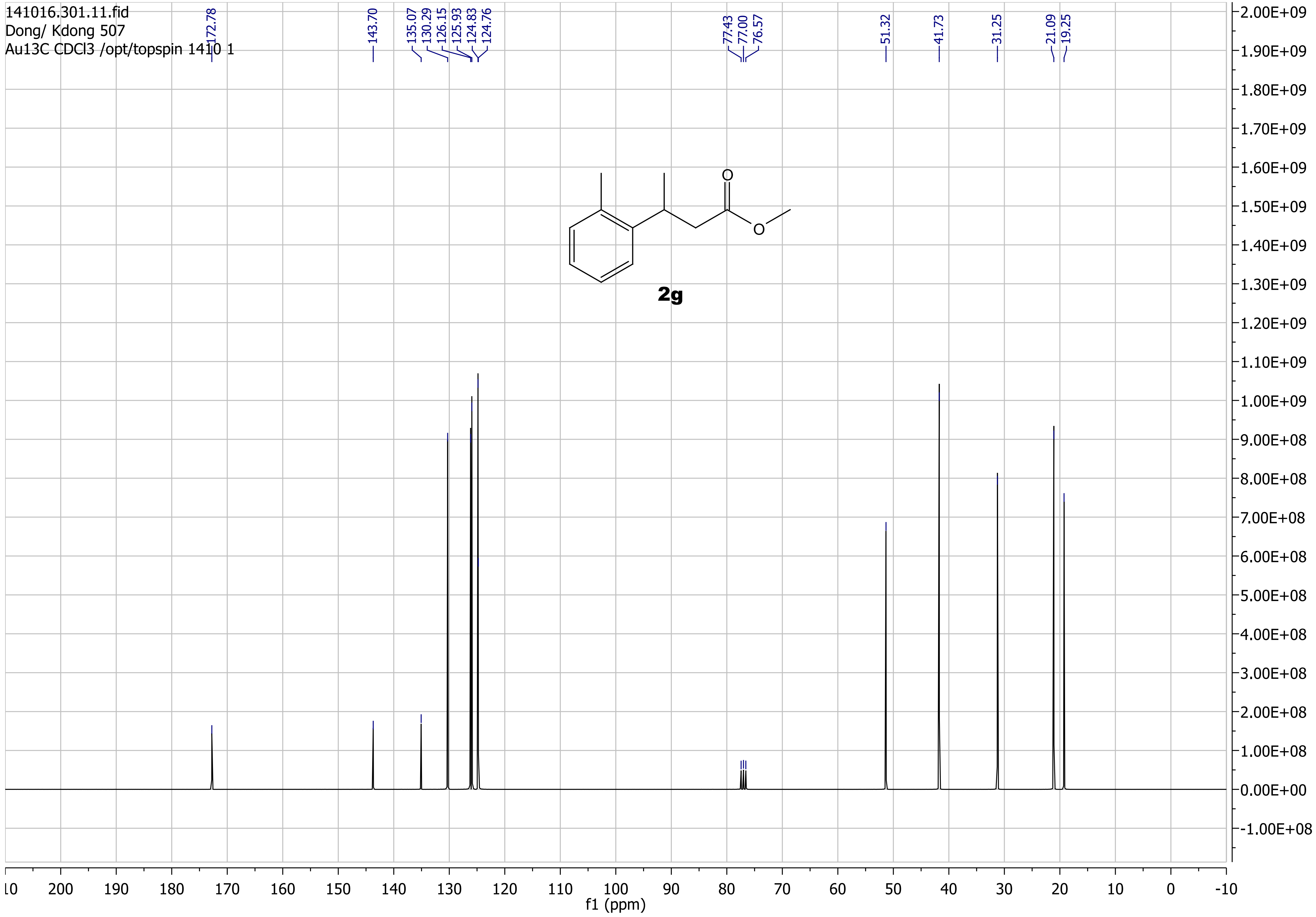
141016.301.11.fid
Dong/ Kdong 507
Au13C CDCl3 /opt/topspin 1410 1
172.78
143.70
135.07
130.29
126.15
125.93
124.83
124.76
77.43
77.00
76.57
51.32
41.73
31.25
21.09
19.25
O
O
2g
f1 (ppm)

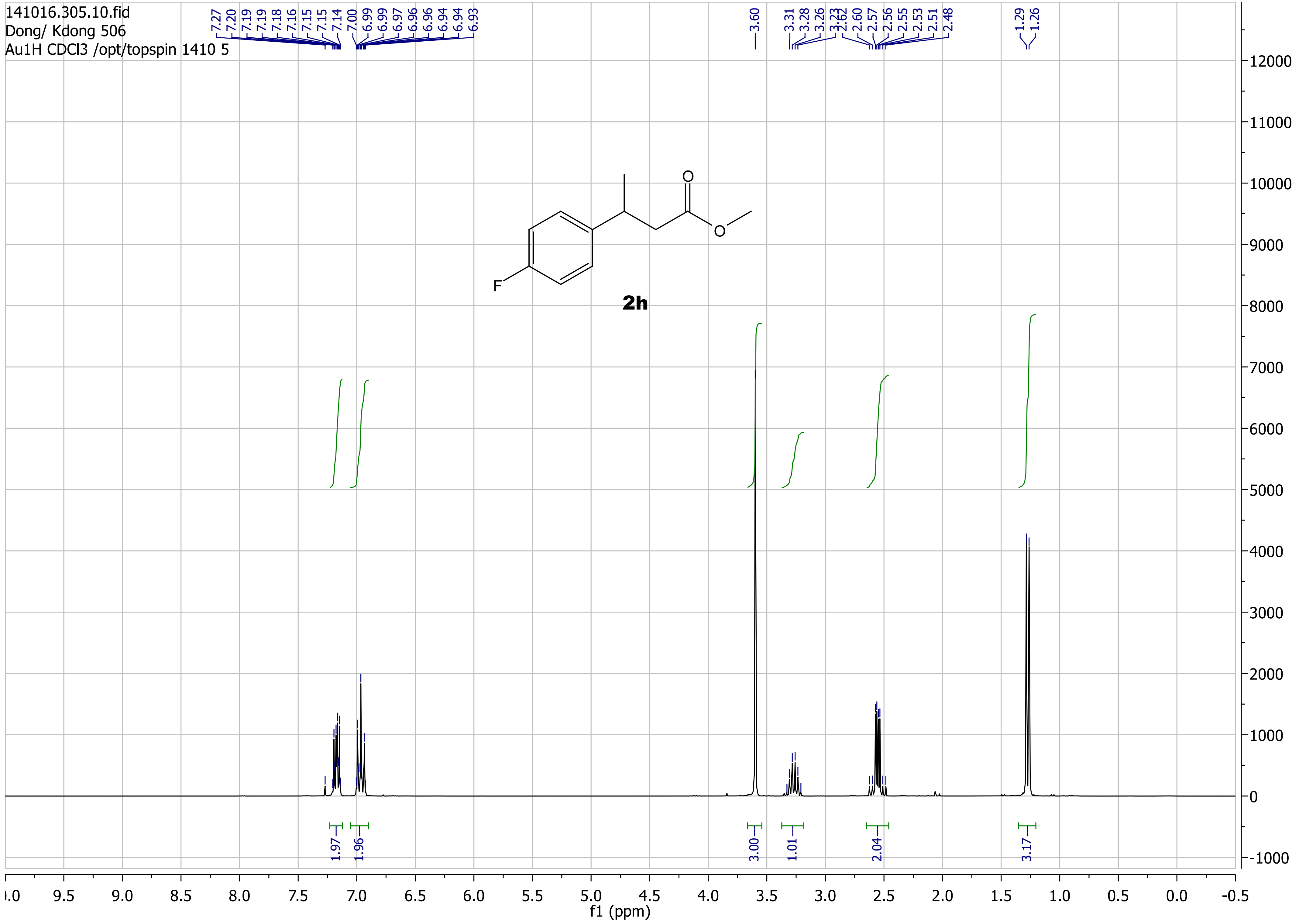

141016.305.10.fid
Dong/ Kdong 506
Au1H CDCl3 /opt/topspin 1410 5
7.27
7.20
7.19
7.19
7.18
7.16
7.15
7.15
7.14
7.00
6.99
6.99
6.97
6.96
6.96
6.94
6.94
6.93
3.60
3.31
3.28
3.26
3.23
2.62
2.60
2.57
2.56
2.55
2.53
2.51
2.48
1.29
1.26
O
O
F
2h
1.97
1.96
3.00
1.01
2.04
3.17
f1 (ppm)

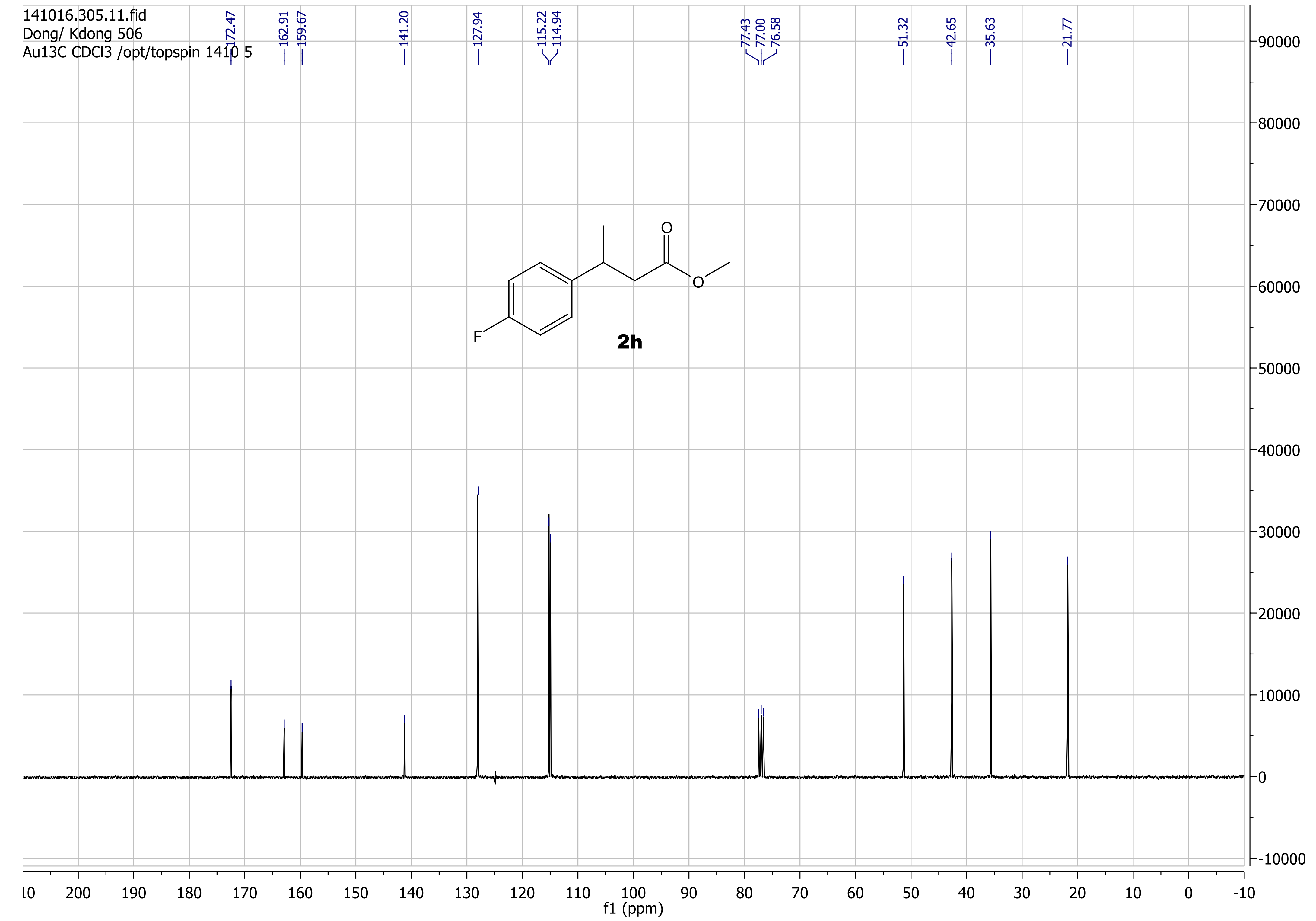
141016.305.11.fid
Dong/ Kdong 506
Au13C CDCl3 /opt/topspin 1410 5
172.47
162.91
159.67
141.20
127.94
115.22
114.94
77.43
77.00
76.58
51.32
42.65
35.63
21.77
O
O
F
2h
f1 (ppm)

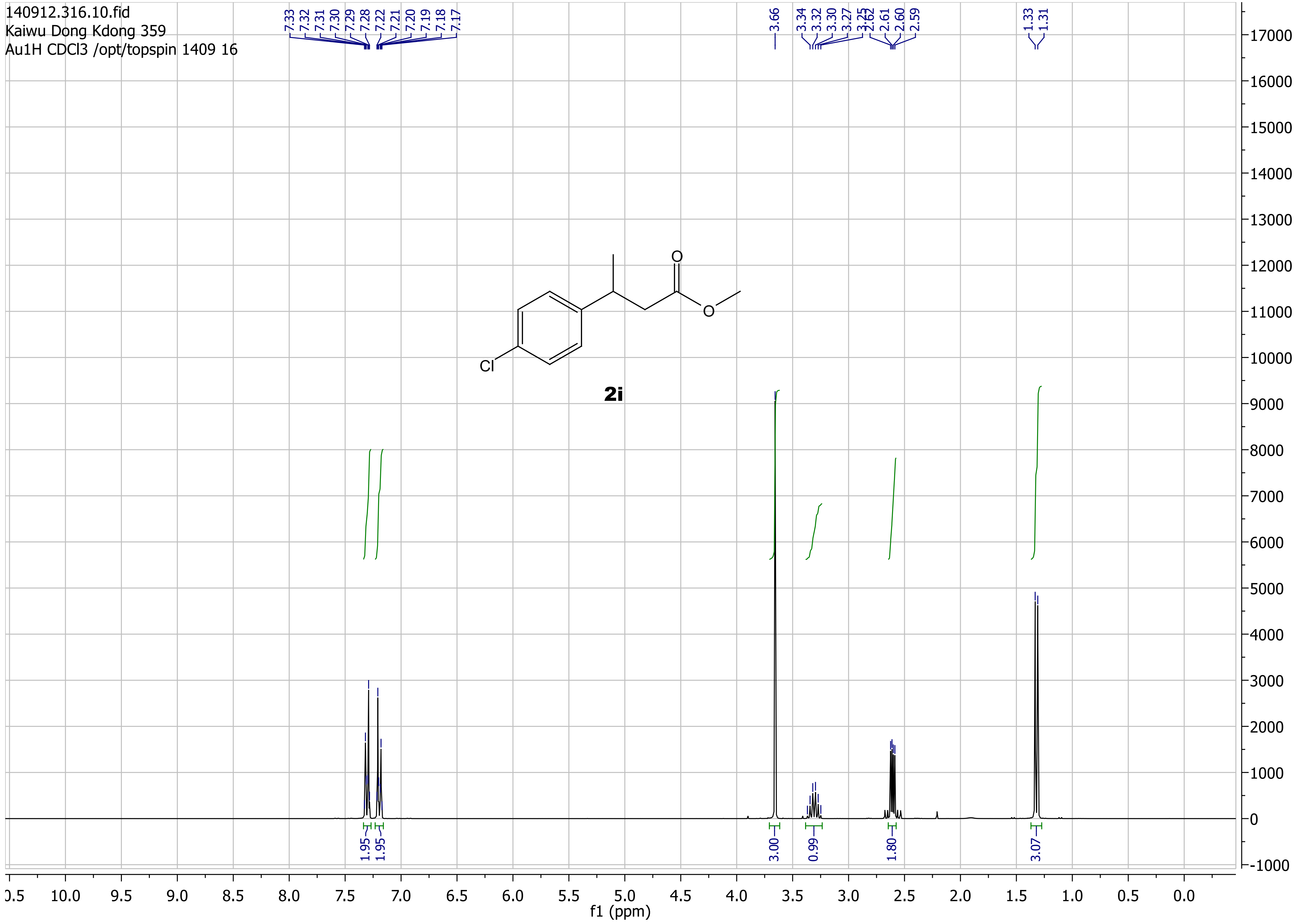
140912.316.10.fid
Kaiwu Dong Kdong 359
Au1H CDCl3 /opt/topspin 1409 16
7.33 7.32 7.31 7.30 7.29 7.28 7.22 7.21 7.20 7.19 7.18 7.17
3.66
3.34 3.32 3.30 3.27 3.25 2.62 2.61 2.60 2.59
1.33 1.31
O
O
Cl
2i
1.95 1.95 3.00 0.99 1.80 3.07
f1 (ppm)

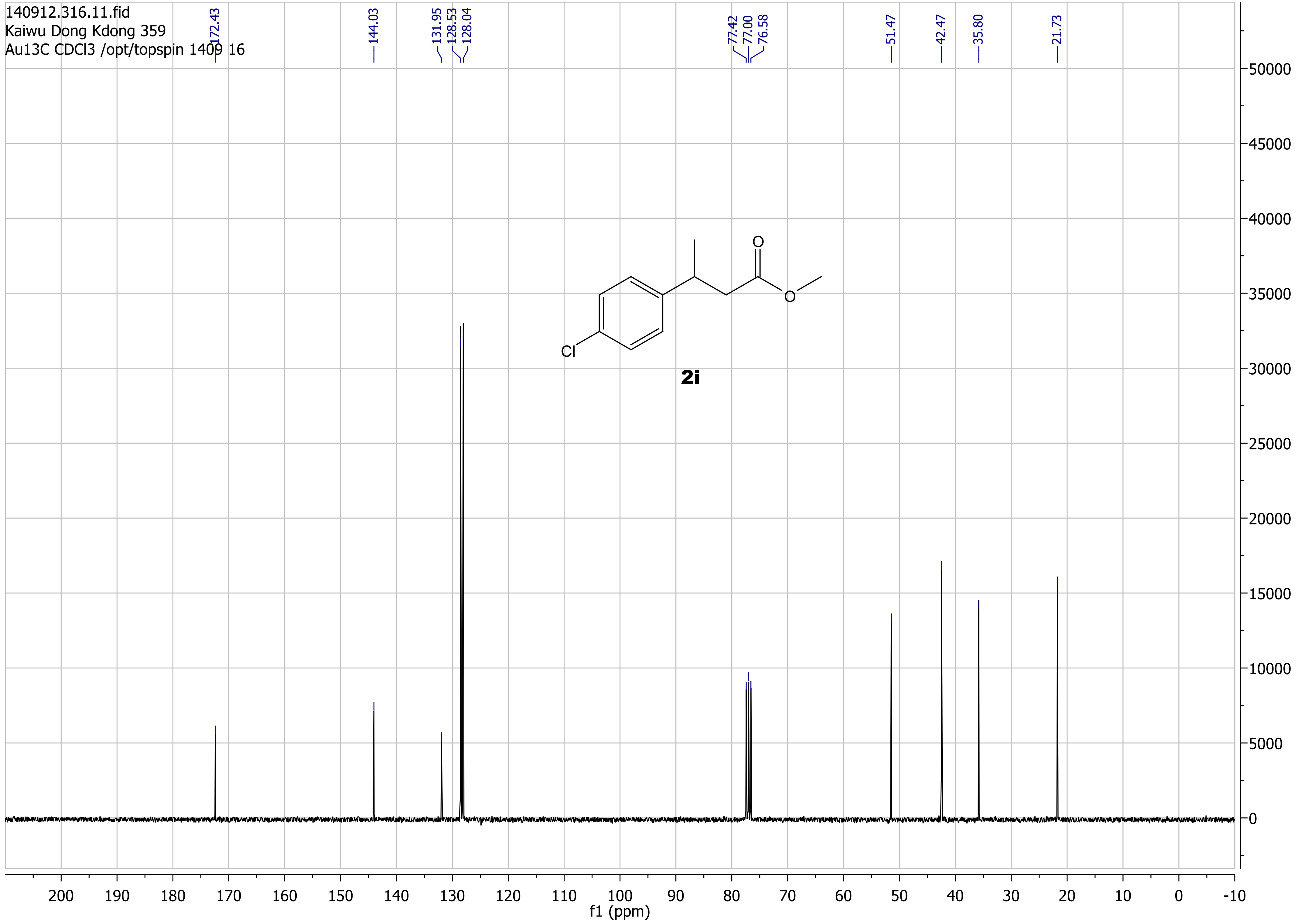
140912.316.11.fid
Kaiwu Dong Kdong 359
Au13C CDCl3 /opt/topspin 1409 16
172.43
144.03
131.95
128.53
128.04
77.42
77.00
76.58
51.47
42.47
35.80
21.73
O
O
Cl
2i
f1 (ppm)

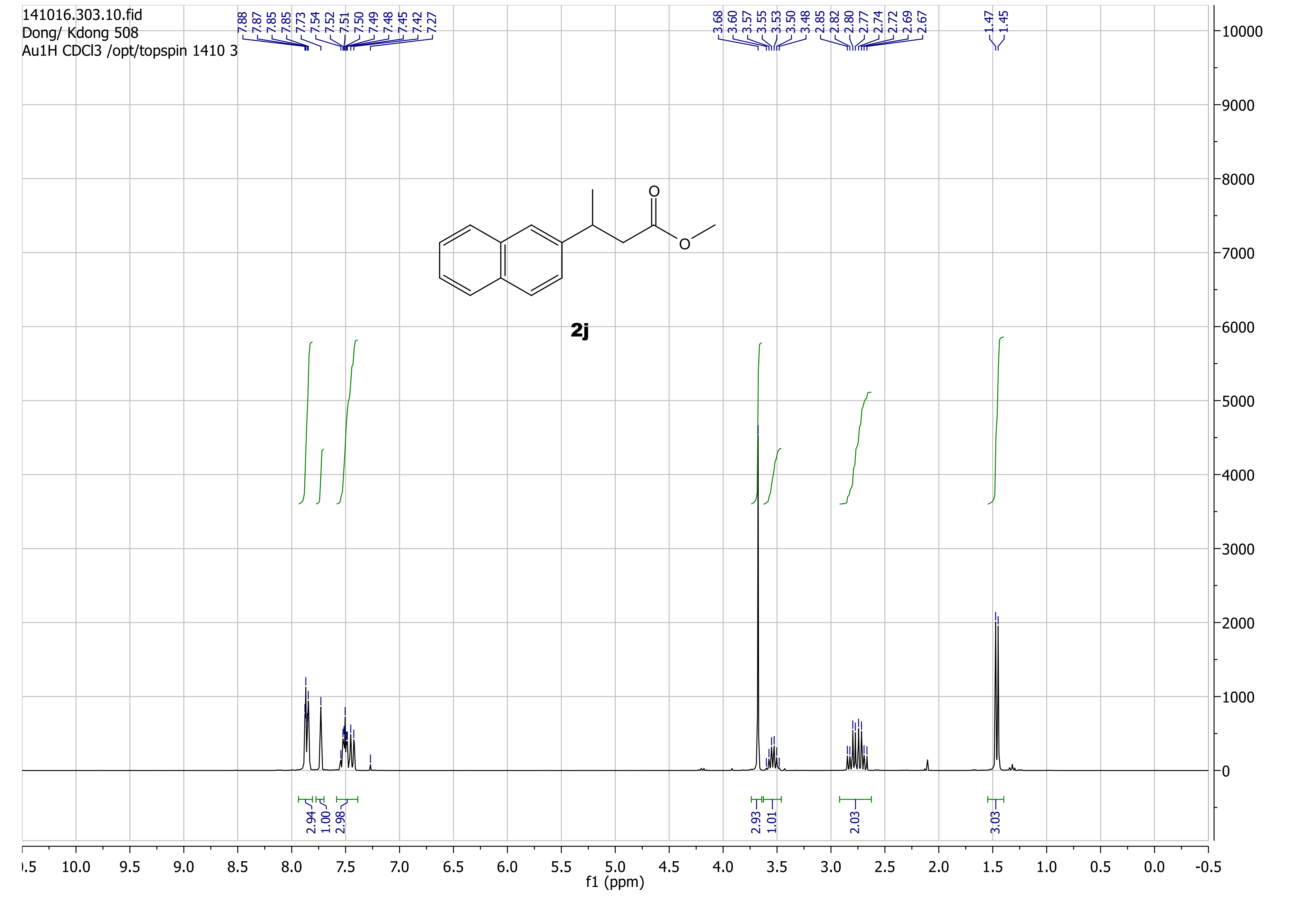
141016.303.10.fid
Dong/ Kdong 508
Au1H CDCl3 /opt/topspin 1410 3
7.88 7.87 7.85 7.85 7.73 7.54 7.52 7.51 7.50 7.49 7.48 7.45 7.42 7.27
3.68 3.60 3.57 3.55 3.53 3.50 3.48 2.85 2.82 2.80 2.77 2.74 2.72 2.69 2.67
1.47 1.45
O
O
2j
2.94 1.00 2.98
2.93 1.01
2.03
3.03
f1 (ppm)

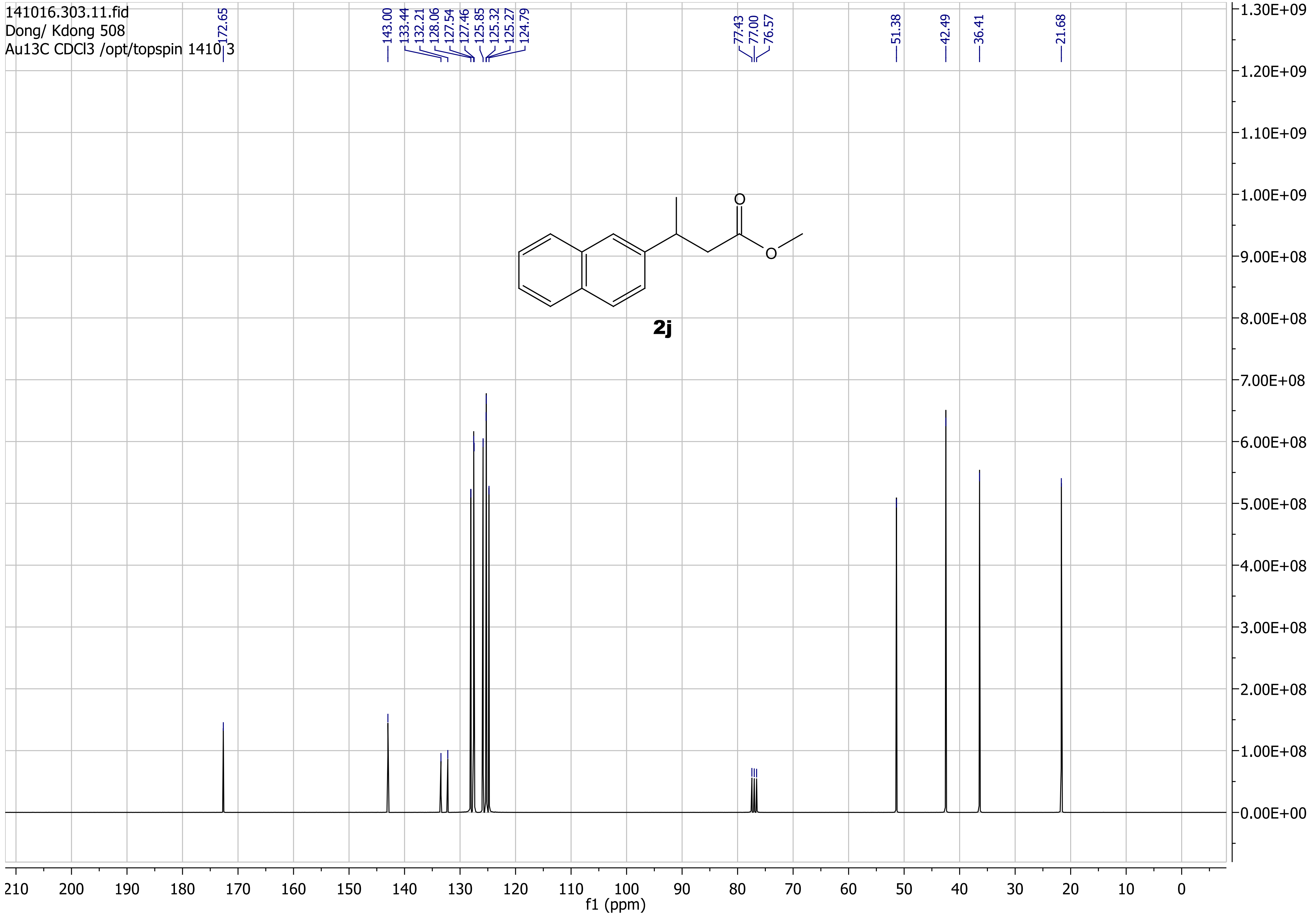
141016.303.11.fid
Dong/ Kdong 508
Au13C CDCl3 /opt/topspin 1410 3
172.65
143.00
133.44
132.21
128.06
127.54
127.46
125.85
125.32
125.27
124.79
77.43
77.00
76.57
51.38
42.49
36.41
21.68
O
O
2j
1.30E+09
1.20E+09
1.10E+09
1.00E+09
9.00E+08
8.00E+08
7.00E+08
6.00E+08
5.00E+08
4.00E+08
3.00E+08
2.00E+08
1.00E+08
0.00E+00
210
200
190
180
170
160
150
140
130
120
110
100
90
80
70
60
50
40
30
20
10
0
f1 (ppm)

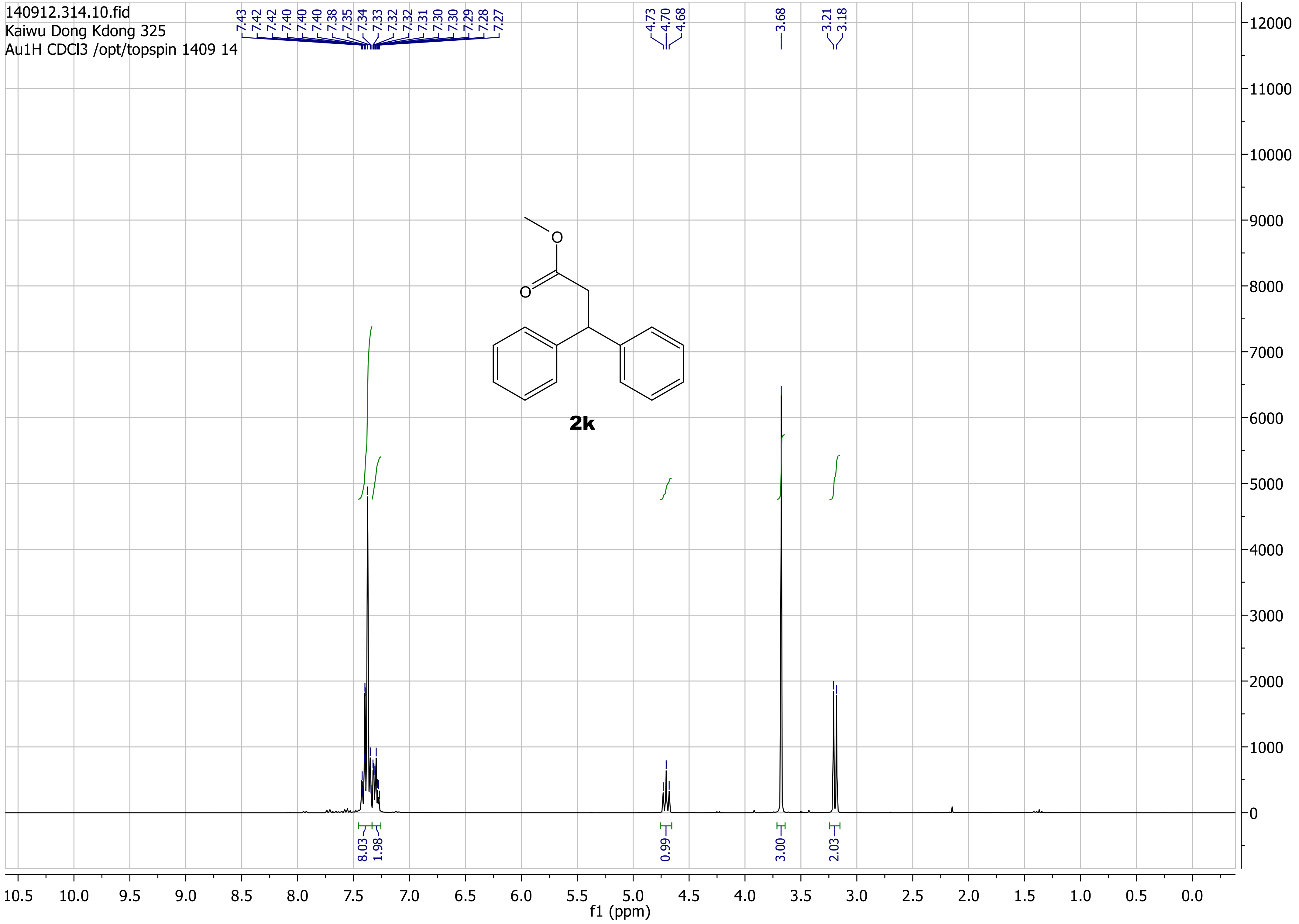
140912.314.10.fid
Kaiwu Dong Kdong 325
Au1H CDCl3 /opt/topspin 1409 14
7.43 7.42 7.42 7.40 7.40 7.40 7.38 7.35 7.34 7.33 7.32 7.32 7.31 7.30 7.30 7.29 7.28 7.27
4.73 4.70 4.68
3.68
3.21 3.18
O
O
2k
8.03
1.98
0.99
3.00
2.03
f1 (ppm)

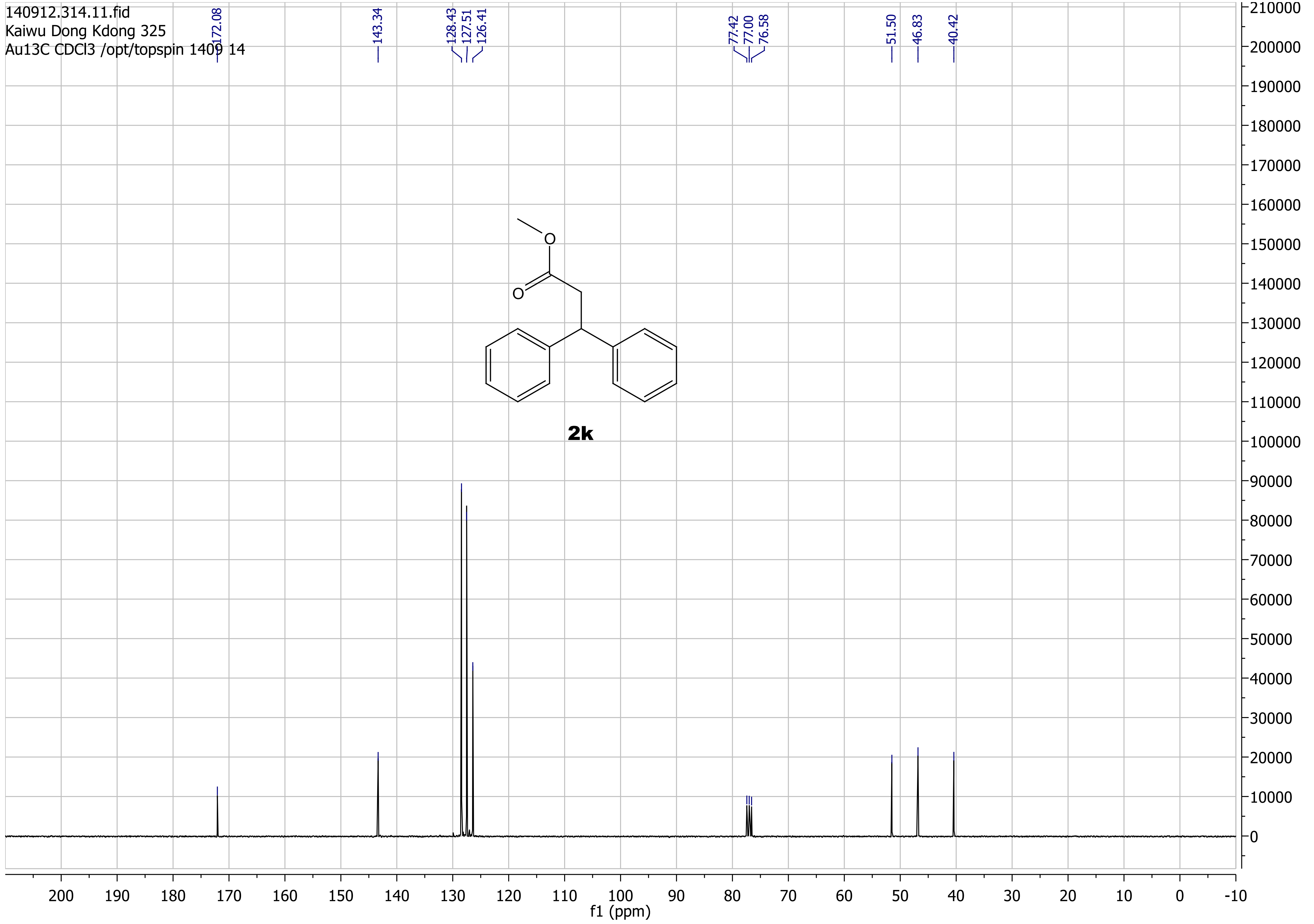
140912.314.11.fid
Kaiwu Dong Kdong 325
Au13C CDCl3 /opt/topspin 1409 14
172.08
143.34
128.43
127.51
126.41
77.42
77.00
76.58
51.50
46.83
40.42
O
O
2k
f1 (ppm)
200
190
180
170
160
150
140
130
120
110
100
90
80
70
60
50
40
30
20
10
0
-10
210000
200000
190000
180000
170000
160000
150000
140000
130000
120000
110000
100000
90000
80000
70000
60000
50000
40000
30000
20000
10000
0

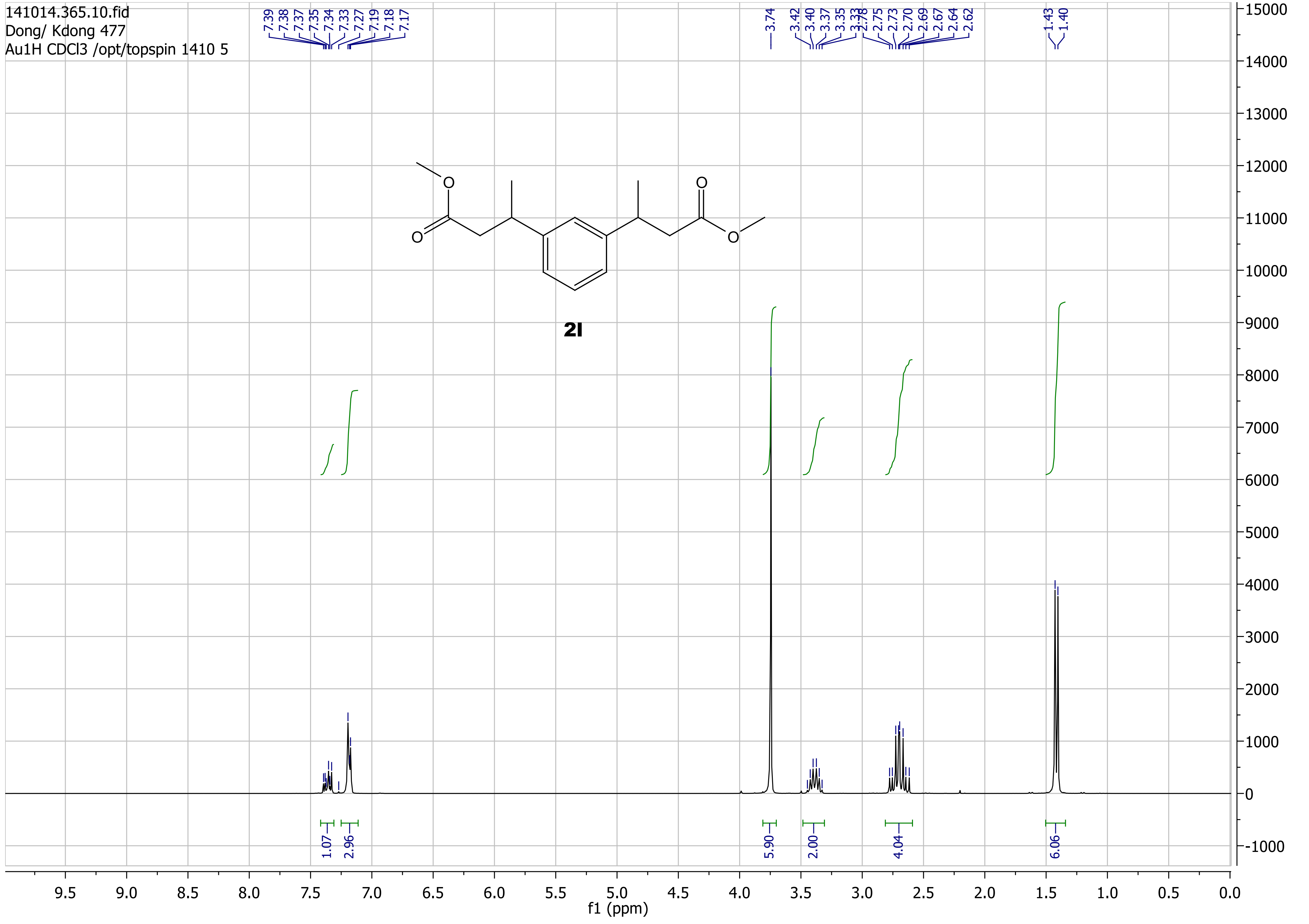
141014.365.10.fid
Dong/ Kdong 477
Au1H CDCl3 /opt/topspin 1410 5
7.39
7.38
7.37
7.35
7.34
7.33
7.27
7.19
7.18
7.17
3.74
3.42
3.40
3.37
3.35
3.33
2.78
2.75
2.73
2.70
2.69
2.67
2.64
2.62
1.43
1.40
O
O
O
O
2l
1.07
2.96
5.90
2.00
4.04
6.06
f1 (ppm)

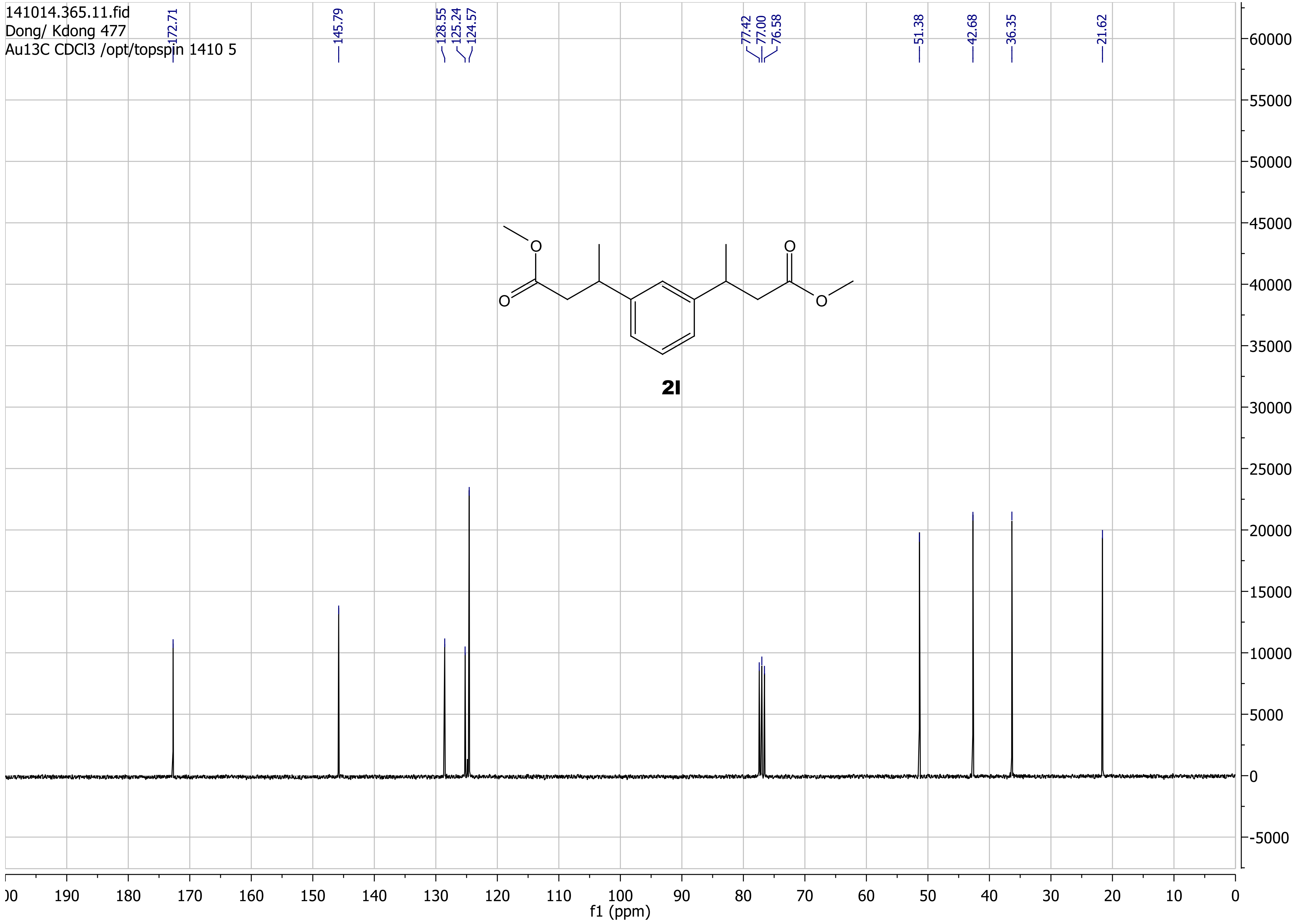

141014.365.11.fid
Dong/ Kdong 477
Au13C CDCl3 /opt/topspin 1410 5
172.71
145.79
128.55
125.24
124.57
77.42
77.00
76.58
51.38
42.68
36.35
21.62
O
O
O
O
2l
f1 (ppm)

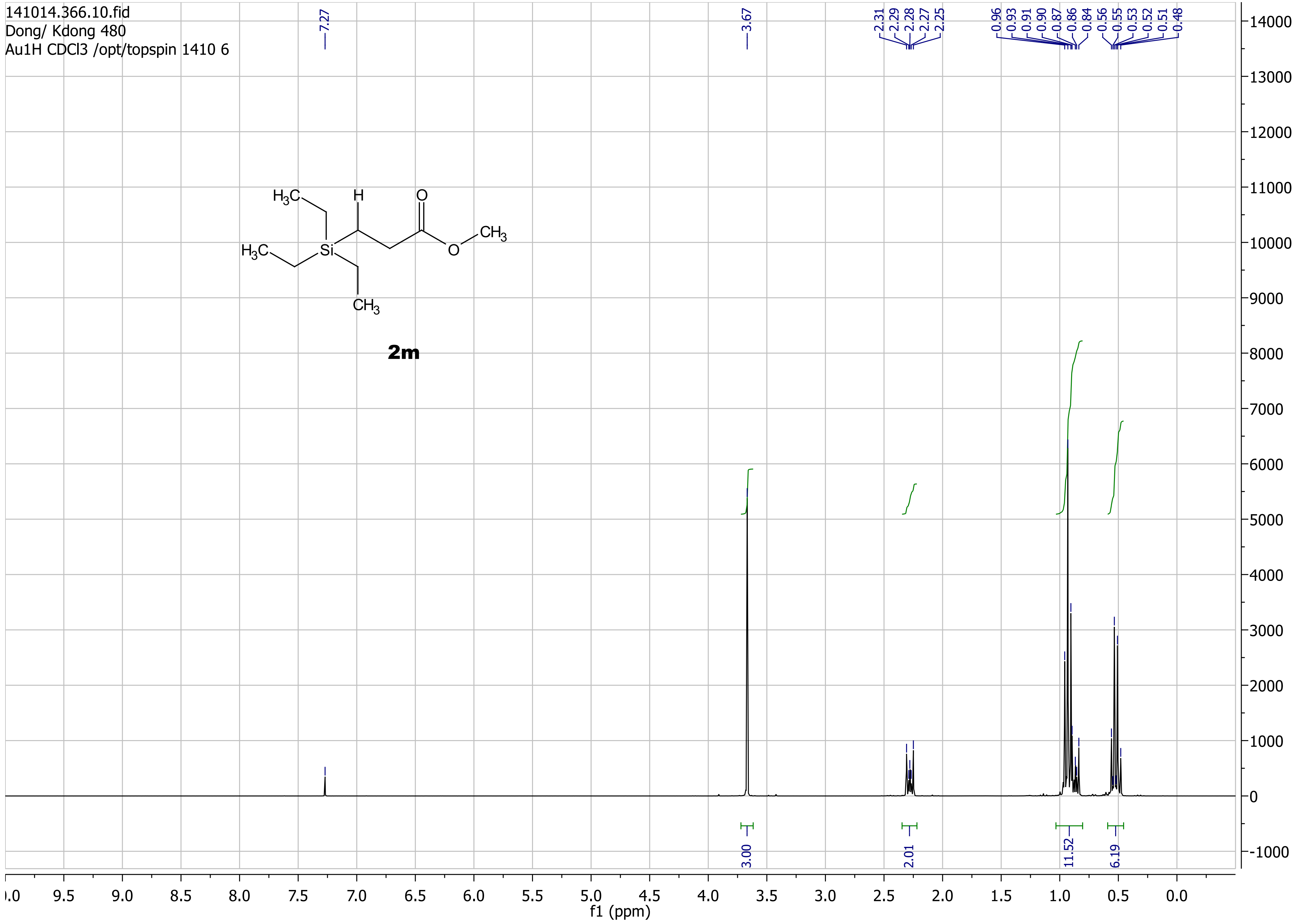
141014.366.10.fid
Dong/ Kdong 480
Au1H CDCl3 /opt/topspin 1410 6
7.27
3.67
2.31
2.29
2.28
2.27
2.25
0.96
0.93
0.91
0.90
0.87
0.86
0.84
0.56
0.55
0.53
0.52
0.51
0.48
H3C
H
O
CH3
H3C
Si
O
CH3
2m
3.00
2.01
11.52
6.19
f1 (ppm)

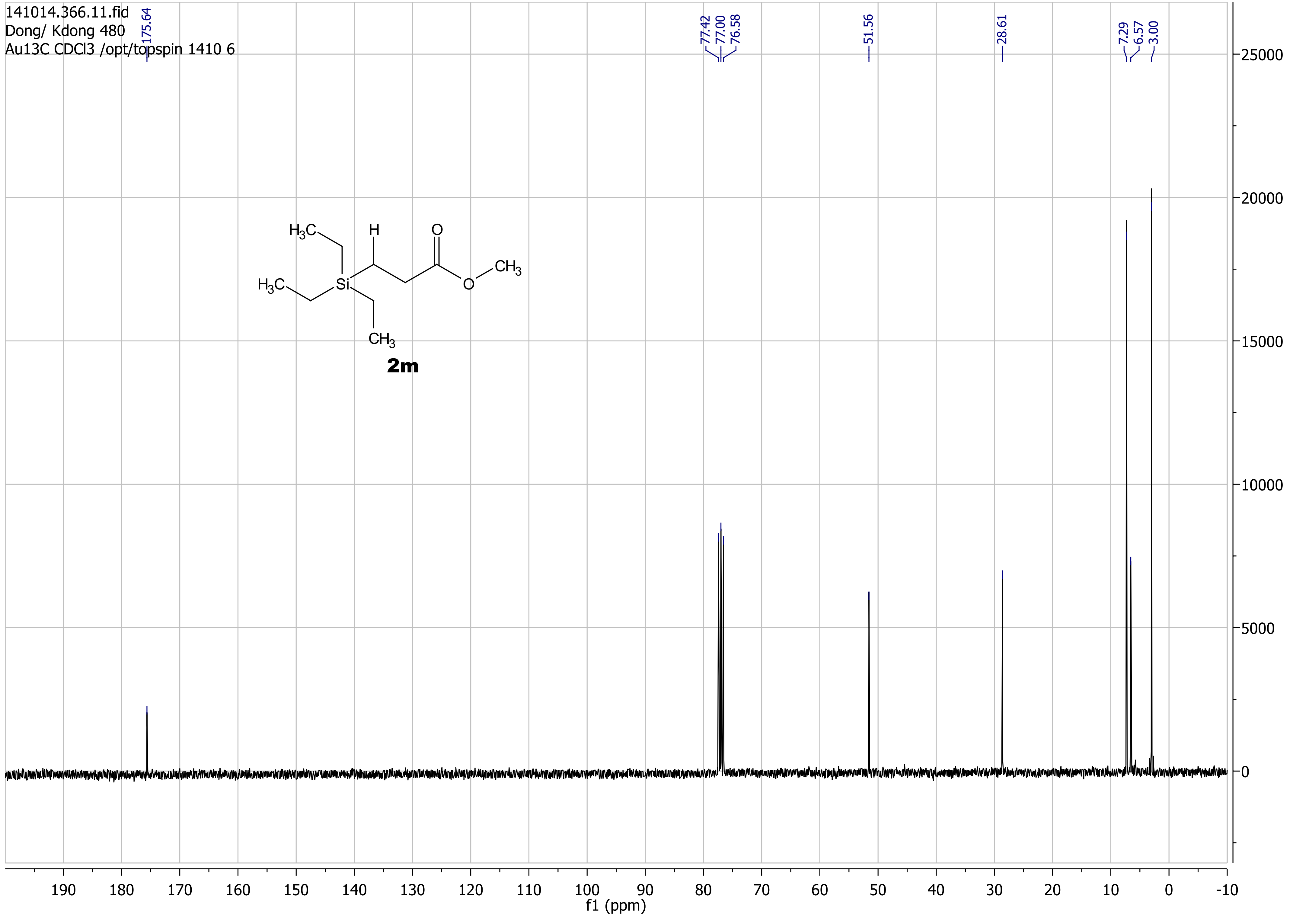

141014.366.11.fid
Dong/ Kdong 480
Au13C CDCl3 /opt/topspin 1410 6
175.64
77.42
77.00
76.58
51.56
28.61
7.29
6.57
3.00
H3C
H
O
CH3
H3C
Si
O
CH3
2m
f1 (ppm)

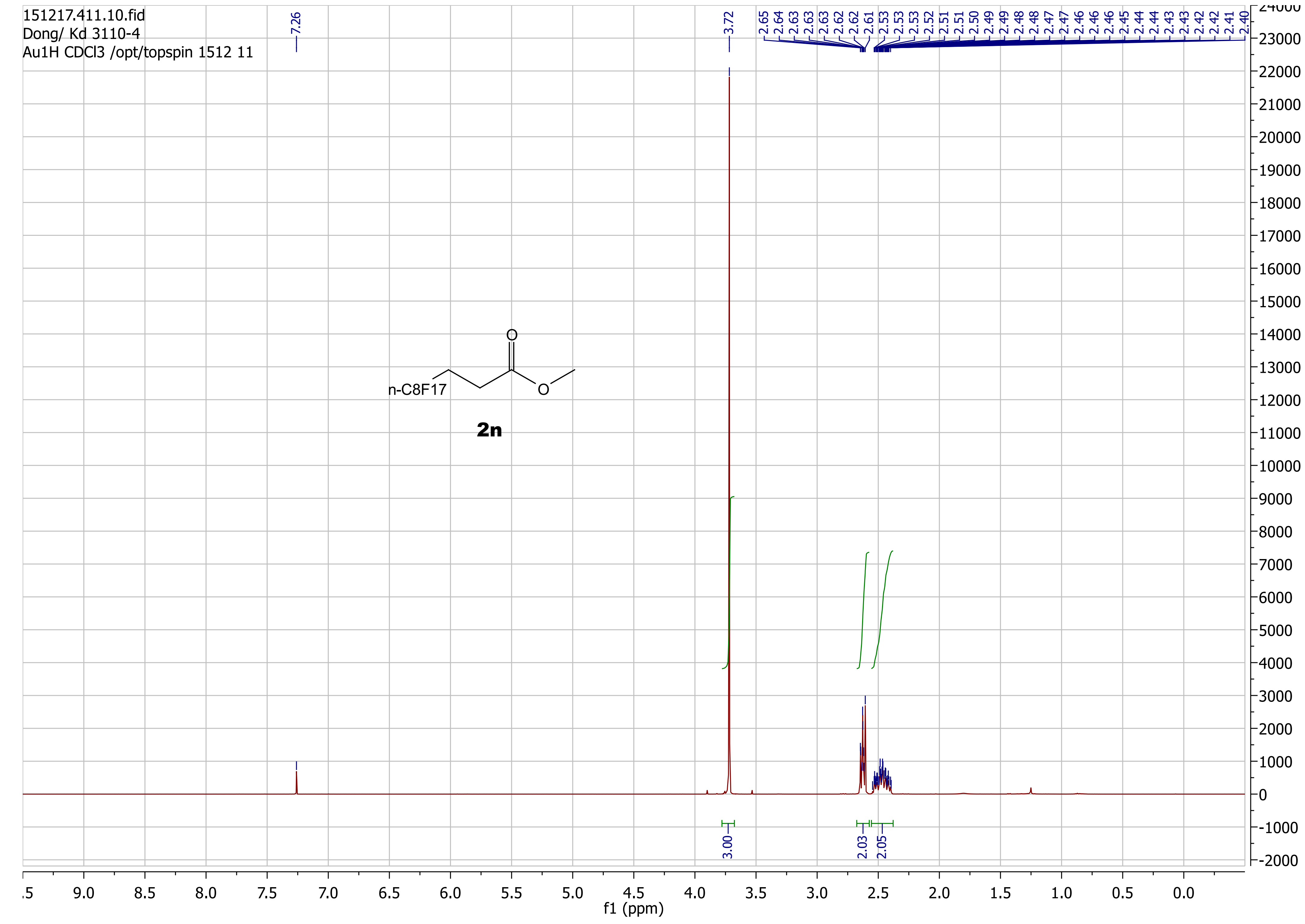

151217.411.10.fid
Dong/ Kd 3110-4
Au1H CDCl3 /opt/topspin 1512 11
7.26
3.72
2.65 2.64 2.63 2.63 2.63 2.62 2.62 2.61 2.53 2.53 2.53 2.52 2.51 2.51 2.50 2.49 2.49 2.48 2.48 2.47 2.47 2.46 2.46 2.46 2.45 2.44 2.44 2.43 2.43 2.42 2.42 2.41 2.40
O
n-C8F17
O
2n
3.00
2.03
2.05
f1 (ppm)

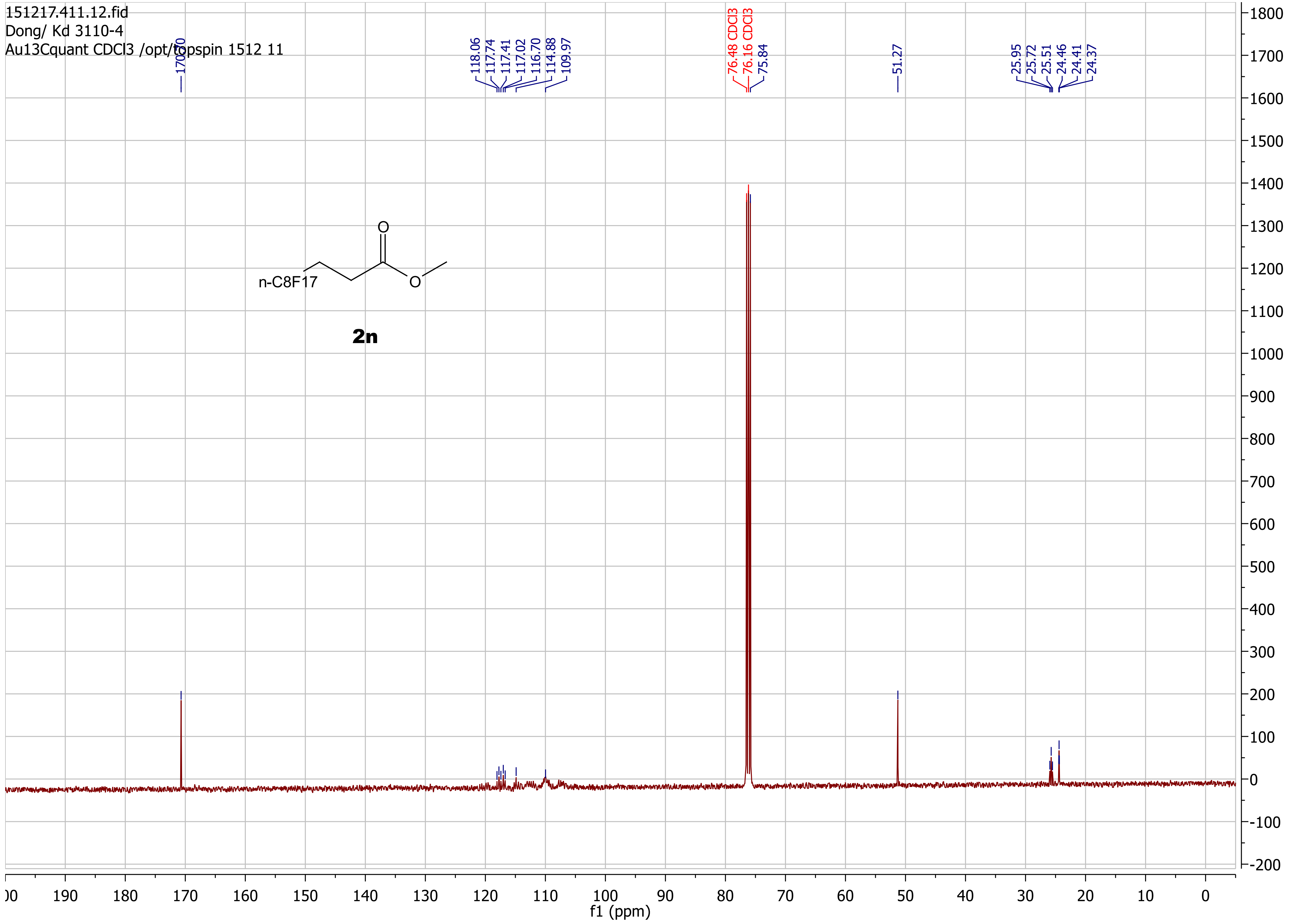
151217.411.12.fid
Dong/ Kd 3110-4
Au13Cquant CDCl3 /opt/topspin 1512 11
170.70
118.06
117.74
117.41
117.02
116.70
114.88
109.97
76.48 CDCl3
76.16 CDCl3
75.84
51.27
25.95
25.72
25.51
24.46
24.41
24.37
O
n-C8F17
O
2n
f1 (ppm)

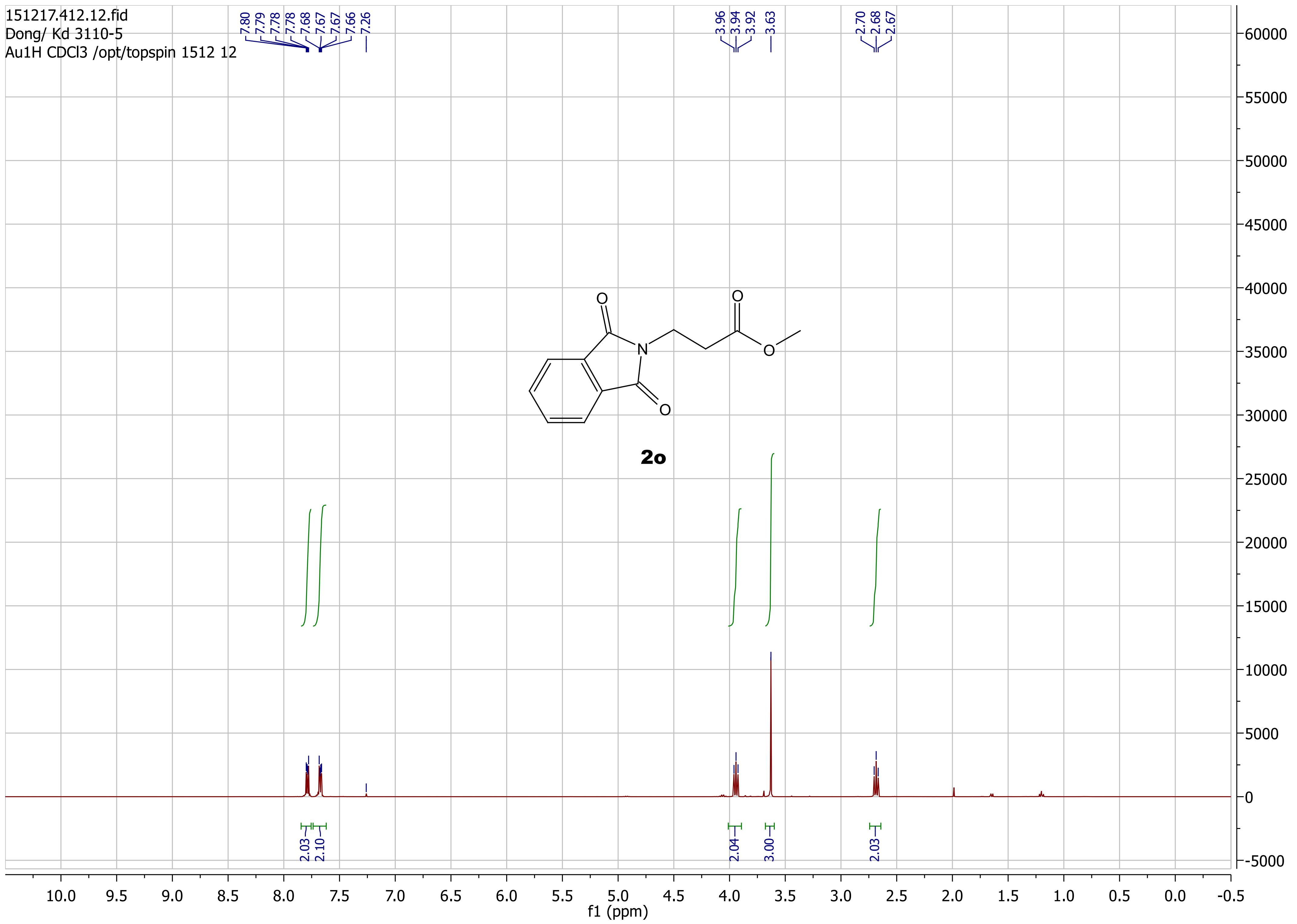
151217.412.12.fid
Dong/ Kd 3110-5
Au1H CDCl3 /opt/topspin 1512 12
7.80
7.79
7.78
7.78
7.68
7.67
7.67
7.66
7.26
3.96
3.94
3.92
3.63
2.70
2.68
2.67
2o
2.03
2.10
2.04
3.00
2.03
f1 (ppm)

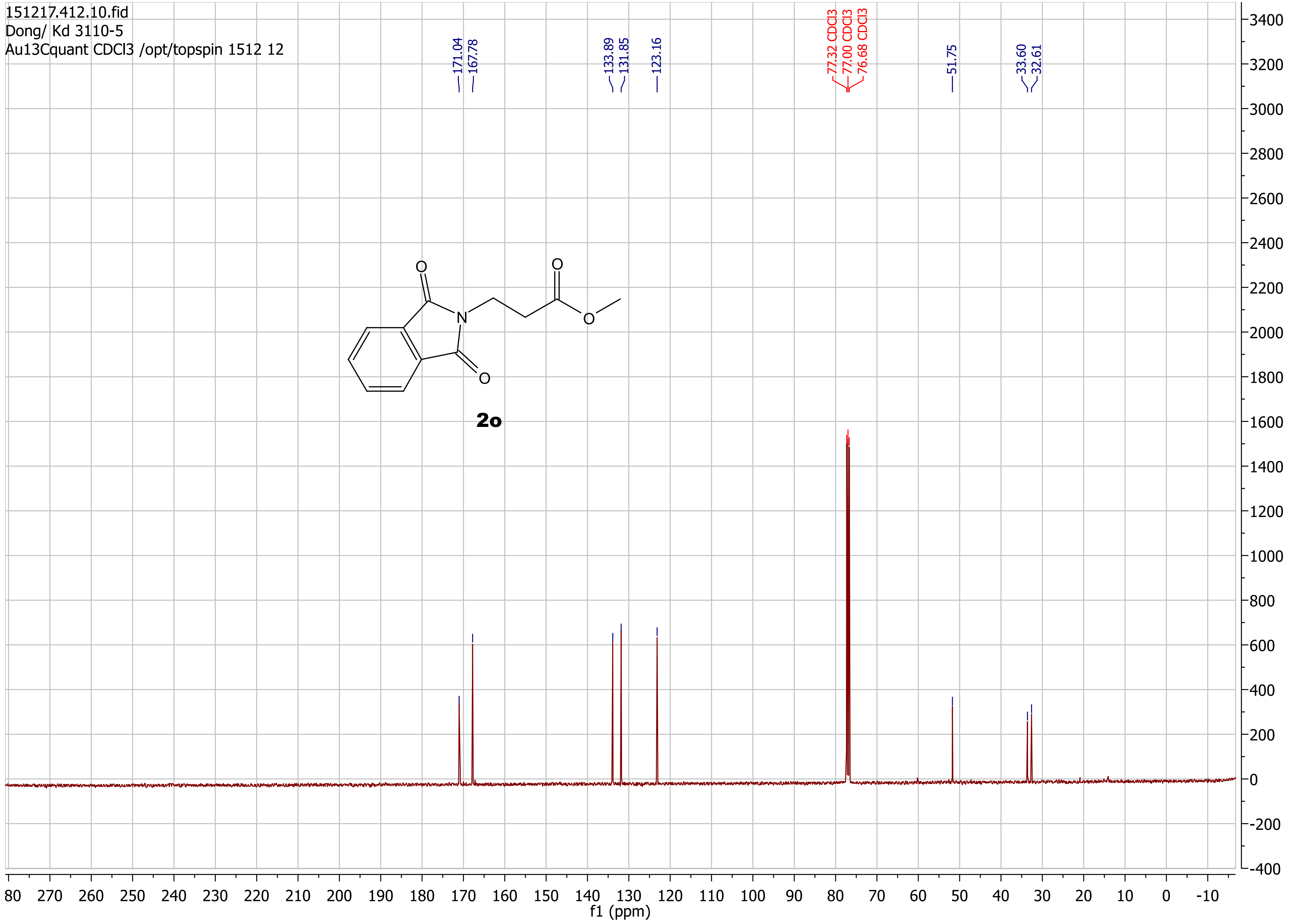
151217.412.10.fid
Dong/ Kd 3110-5
Au13Cquant CDCl3 /opt/topspin 1512 12
171.04
167.78
133.89
131.85
123.16
77.32 CDCl3
77.00 CDCl3
76.68 CDCl3
51.75
33.60
32.61
O
O
N
O
O
2o
f1 (ppm)

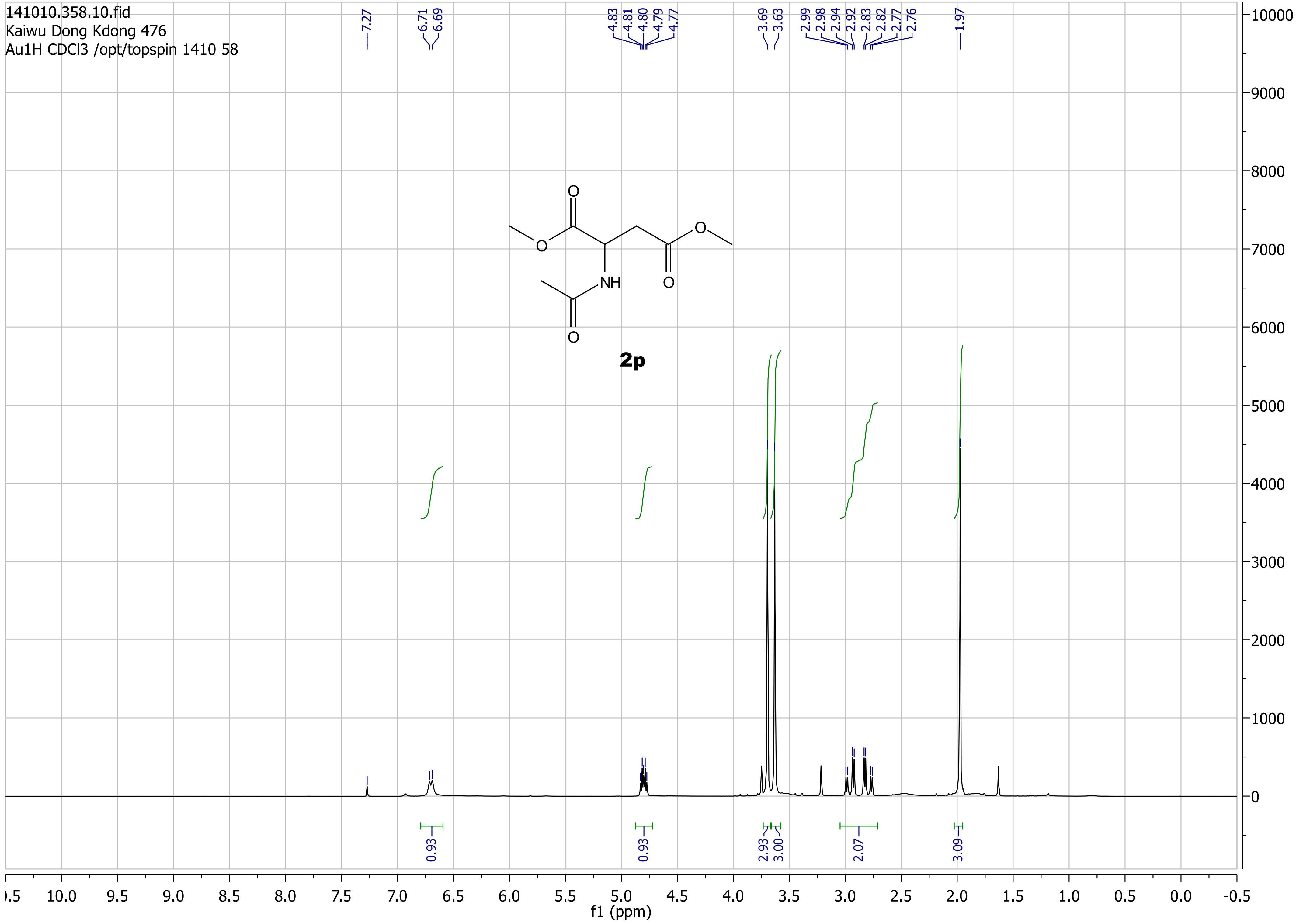
141010.358.10.fid
Kaiwu Dong Kdong 476
Au1H CDCl3 /opt/topspin 1410 58
7.27
6.71
6.69
4.83
4.81
4.80
4.79
4.77
3.69
3.63
2.99
2.98
2.94
2.92
2.83
2.82
2.77
2.76
1.97
O
O
NH
O
O
O
2p
0.93
0.93
2.93
3.00
2.07
3.09
10000
9000
8000
7000
6000
5000
4000
3000
2000
1000
0
10.0
9.5
9.0
8.5
8.0
7.5
7.0
6.5
6.0
5.5
5.0
4.5
4.0
3.5
3.0
2.5
2.0
1.5
1.0
0.5
0.0
-0.5
f1 (ppm)

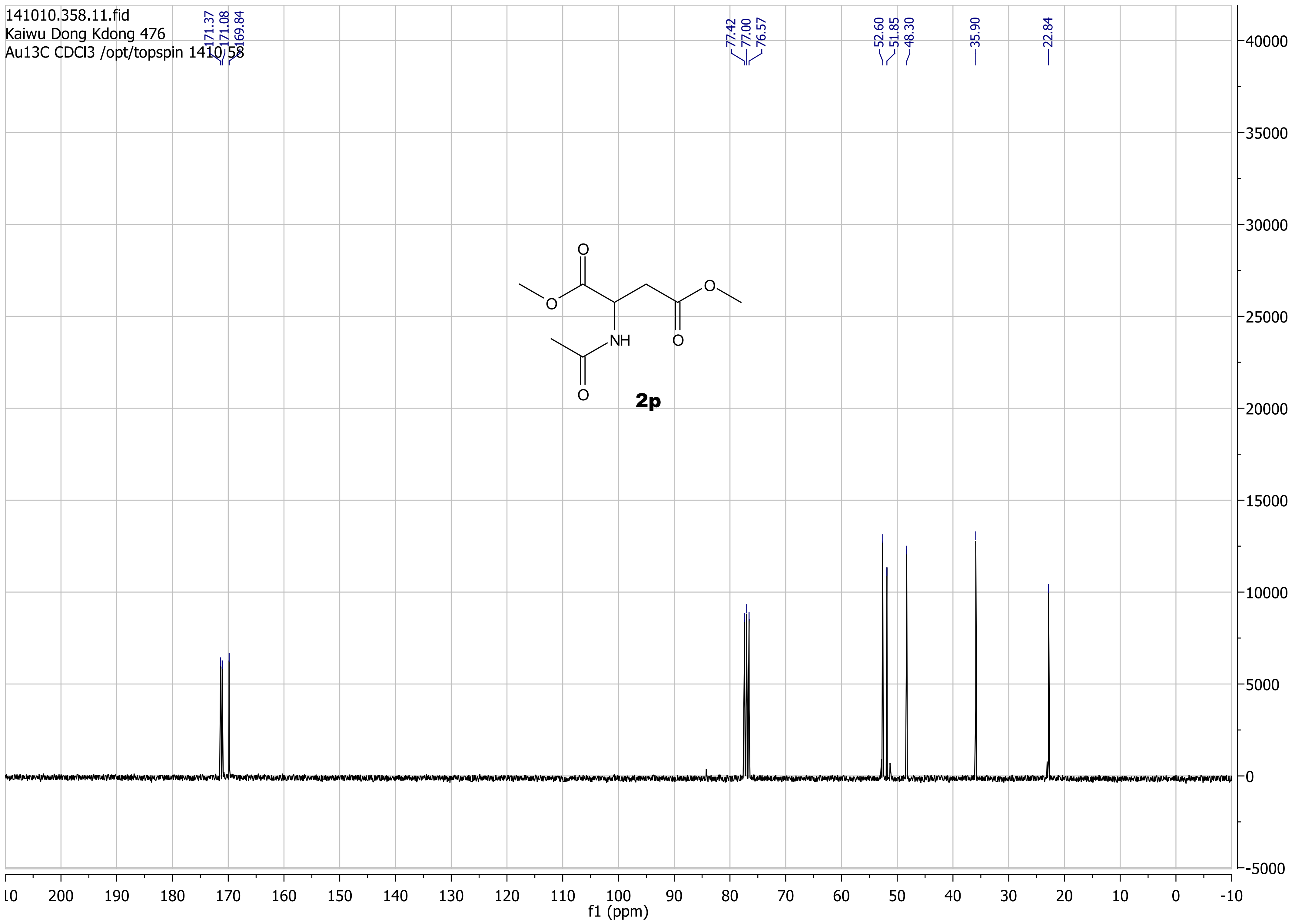
141010.358.11.fid
Kaiwu Dong Kdong 476
Au13C CDCl3 /opt/topspin 1410 58
171.37
171.08
169.84
77.42
77.00
76.57
52.60
51.85
48.30
35.90
22.84
O
O
O
O
O
NH
2p
f1 (ppm)

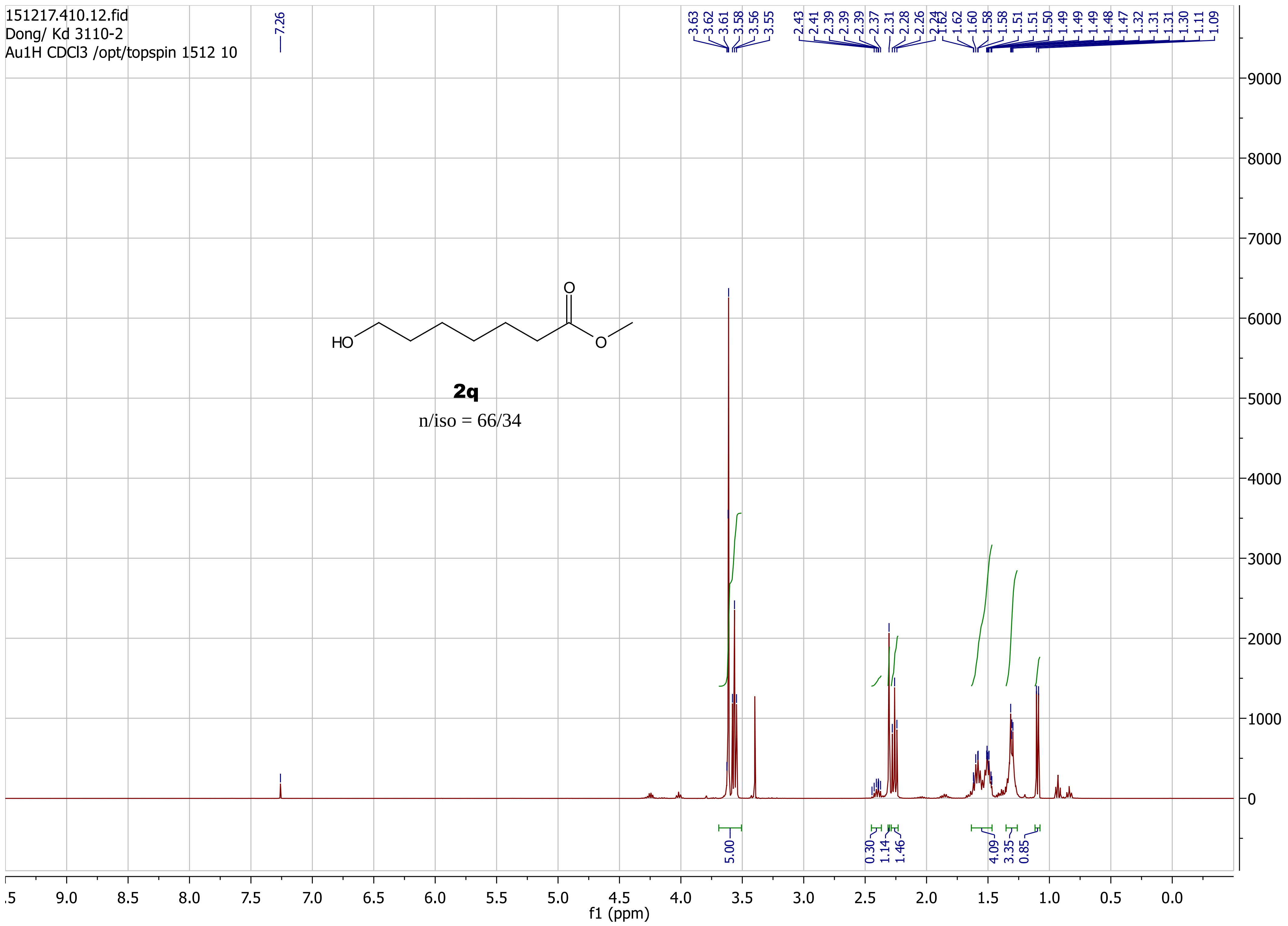
151217.410.12.fid
Dong/ Kd 3110-2
Au1H CDCl3 /opt/topspin 1512 10
7.26
3.63
3.62
3.61
3.58
3.56
3.55
2.43
2.41
2.39
2.39
2.39
2.37
2.31
2.28
2.26
2.24
1.62
1.62
1.60
1.58
1.58
1.51
1.51
1.50
1.49
1.49
1.49
1.48
1.47
1.32
1.31
1.31
1.30
1.11
1.09
HO
O
O
2q
n/iso = 66/34
5.00
0.30
1.14
1.46
4.09
3.35
0.85
f1 (ppm)

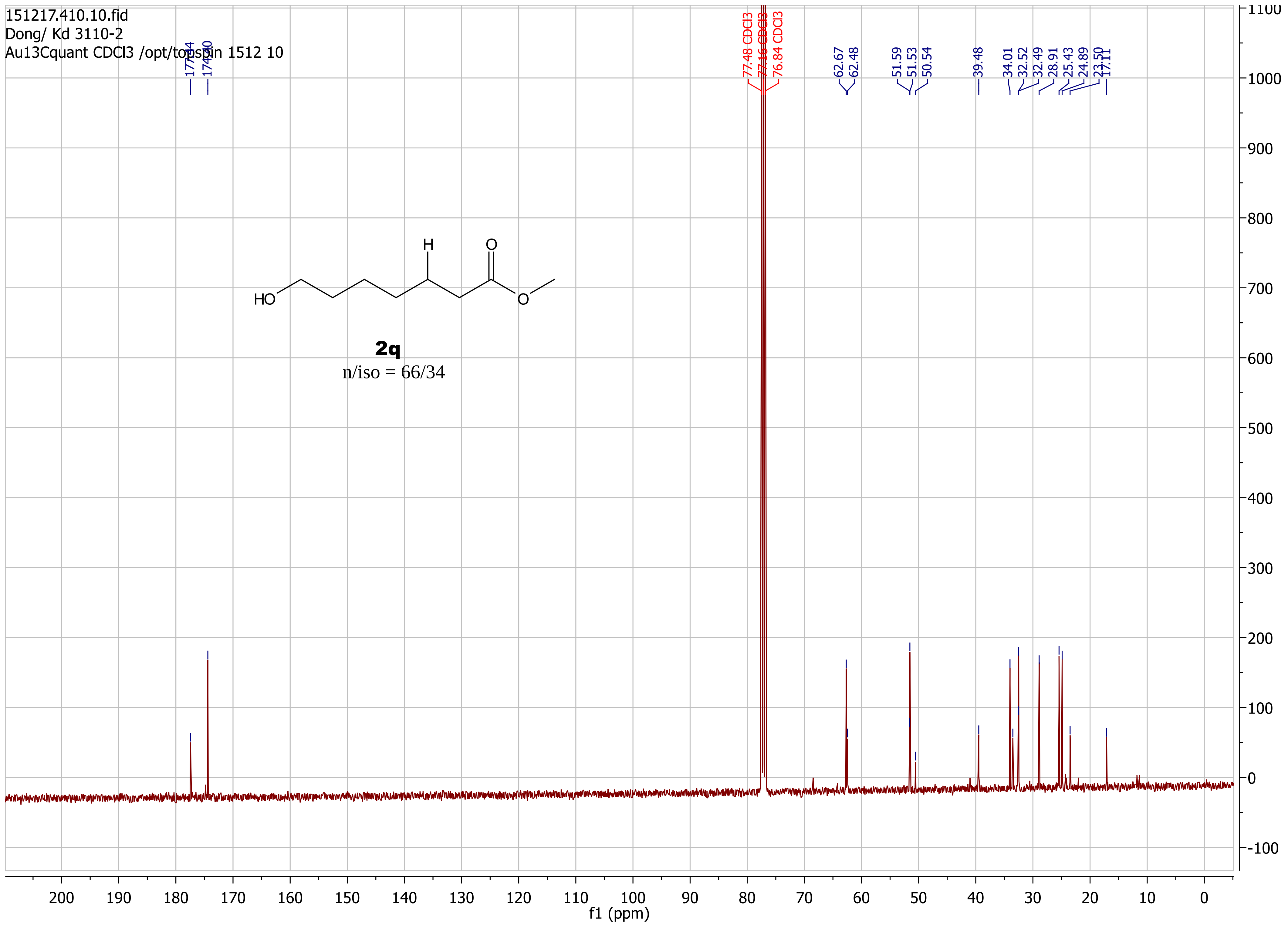
151217.410.10.fid
Dong/ Kd 3110-2
Au13Cquant CDCl3 /opt/topspin 1512 10
177.34
174.40
77.48 CDCl3
77.16 CDCl3
76.84 CDCl3
62.67
62.48
51.59
51.53
50.54
39.48
34.01
32.52
32.49
28.91
25.43
24.89
23.50
17.11
H
O
HO
O
2q
n/iso = 66/34
f1 (ppm)

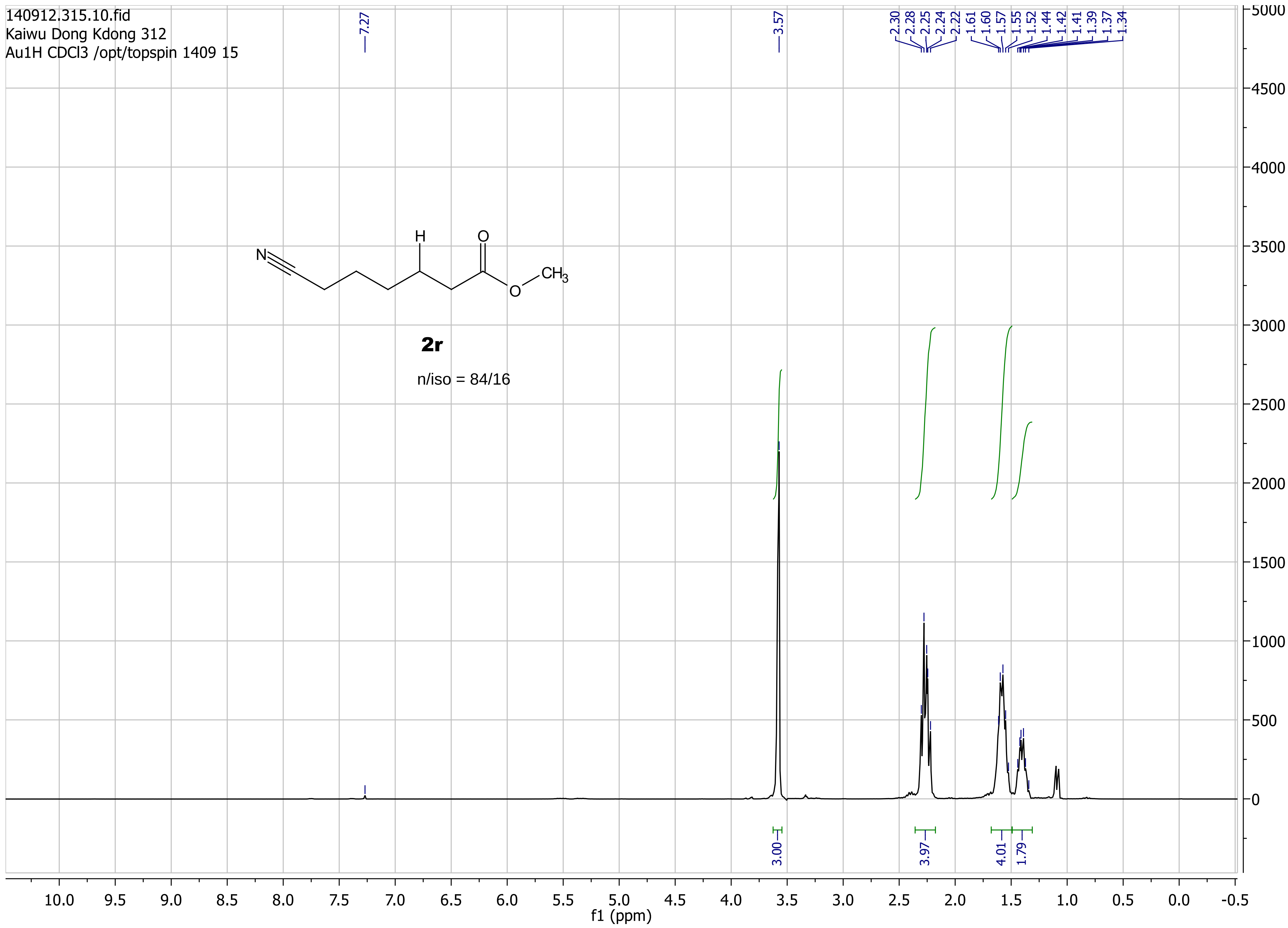

140912.315.10.fid
Kaiwu Dong Kdong 312
Au1H CDCl3 /opt/topspin 1409 15
7.27
3.57
2.30
2.28
2.25
2.24
2.22
1.61
1.60
1.57
1.55
1.52
1.44
1.42
1.41
1.39
1.37
1.34
N
H
O
O
CH3
2r
n/iso = 84/16
3.00
3.97
4.01
1.79
f1 (ppm)

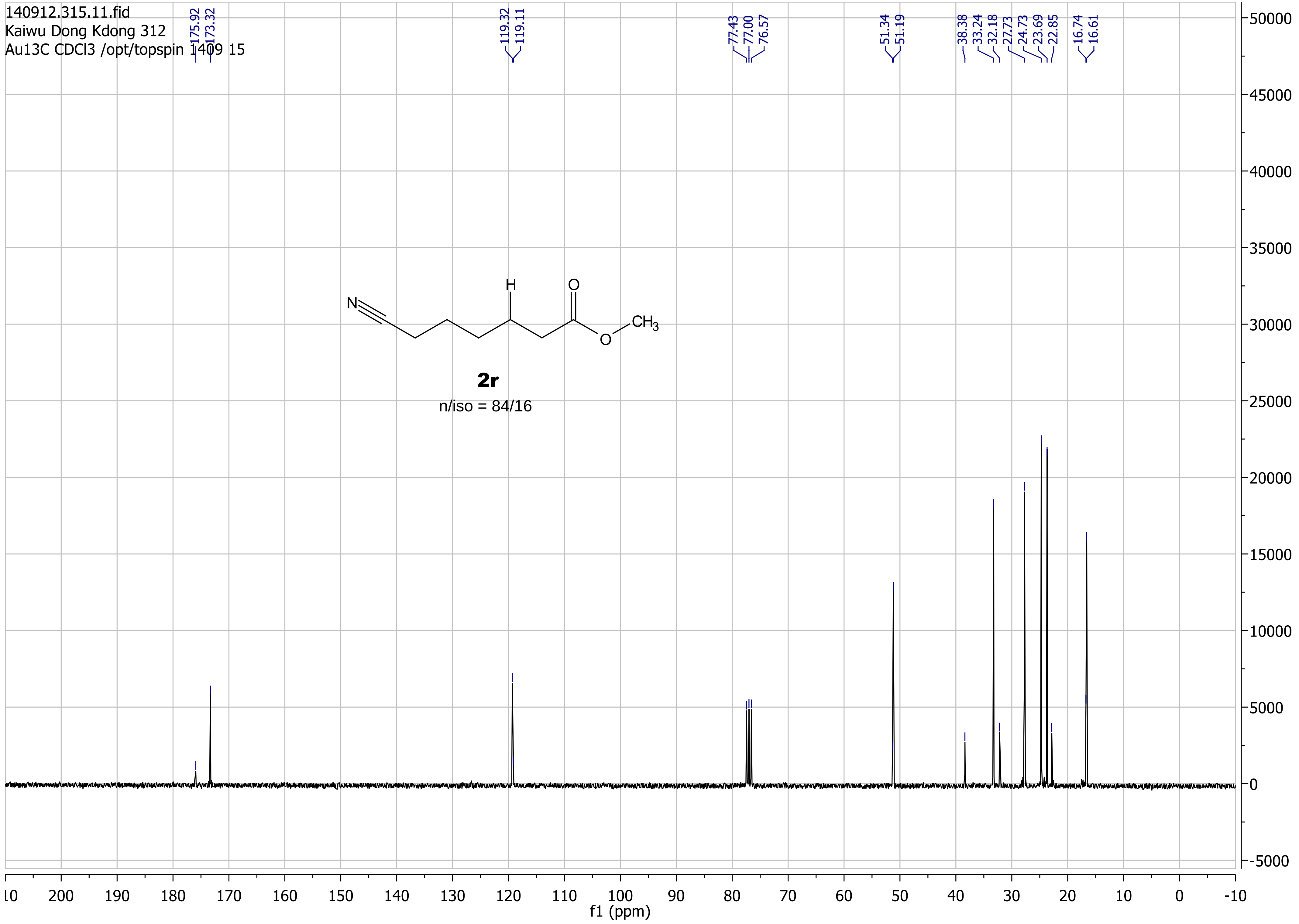
140912.315.11.fid
Kaiwu Dong Kdong 312
Au13C CDCl3 /opt/topspin 1409 15
175.92
173.32
119.32
119.11
77.43
77.00
76.57
51.34
51.19
38.38
33.24
32.18
27.73
24.73
23.69
22.85
16.74
16.61
N
H
O
O
CH3
2r
n/iso = 84/16
f1 (ppm)

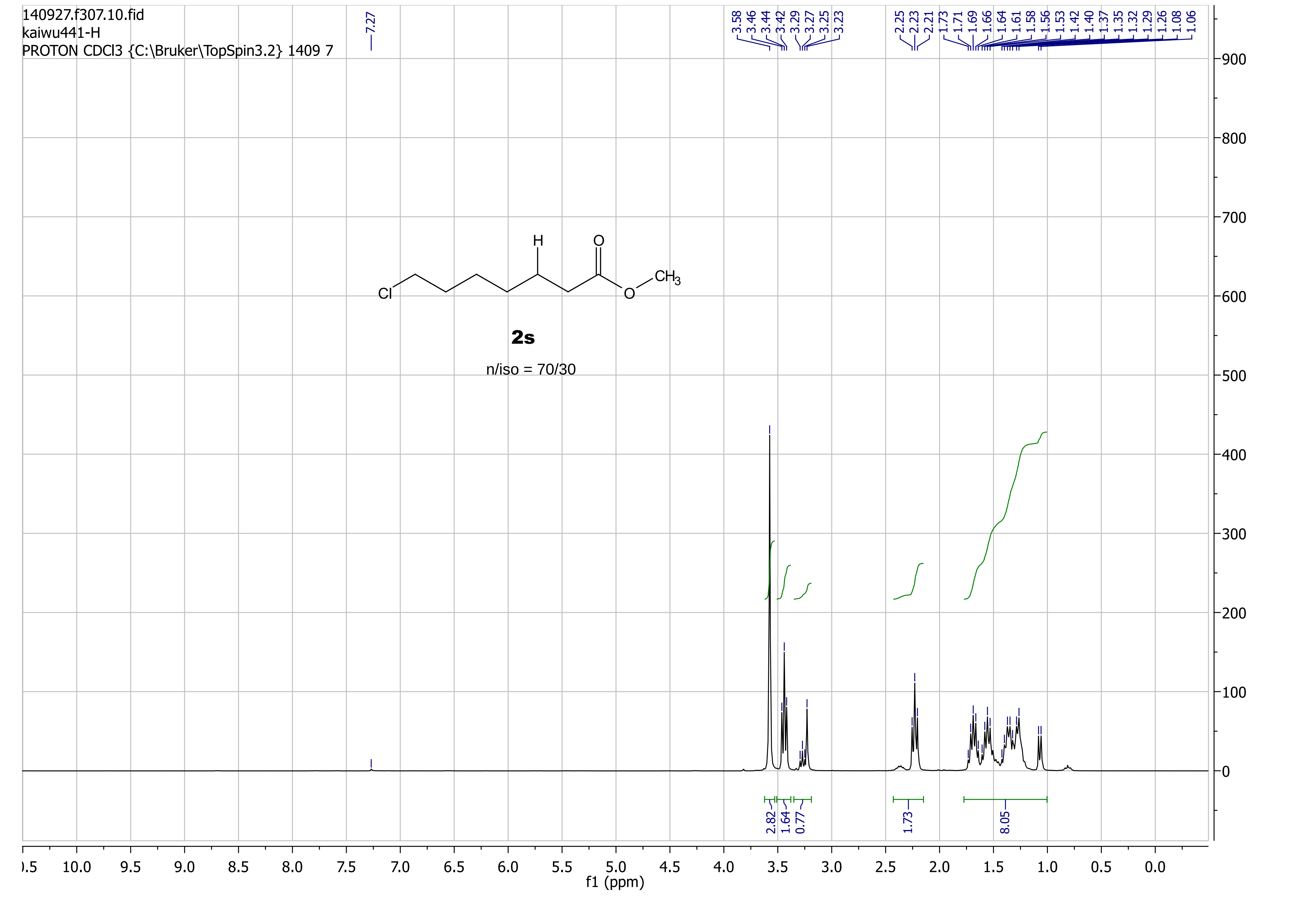
140927.f307.10.fid
kaiwu441-H
PROTON CDCl3 {C:\Bruker\TopSpin3.2} 1409 7
7.27
3.58 3.46 3.44 3.42 3.29 3.27 3.25 3.23
2.25 2.23 2.21 1.73 1.71 1.69 1.66 1.64 1.61 1.58 1.56 1.53 1.42 1.40 1.37 1.35 1.32 1.29 1.26 1.08 1.06
H
O
Cl
O
CH3
2s
n/iso = 70/30
2.82
1.64
0.77
1.73
8.05
f1 (ppm)

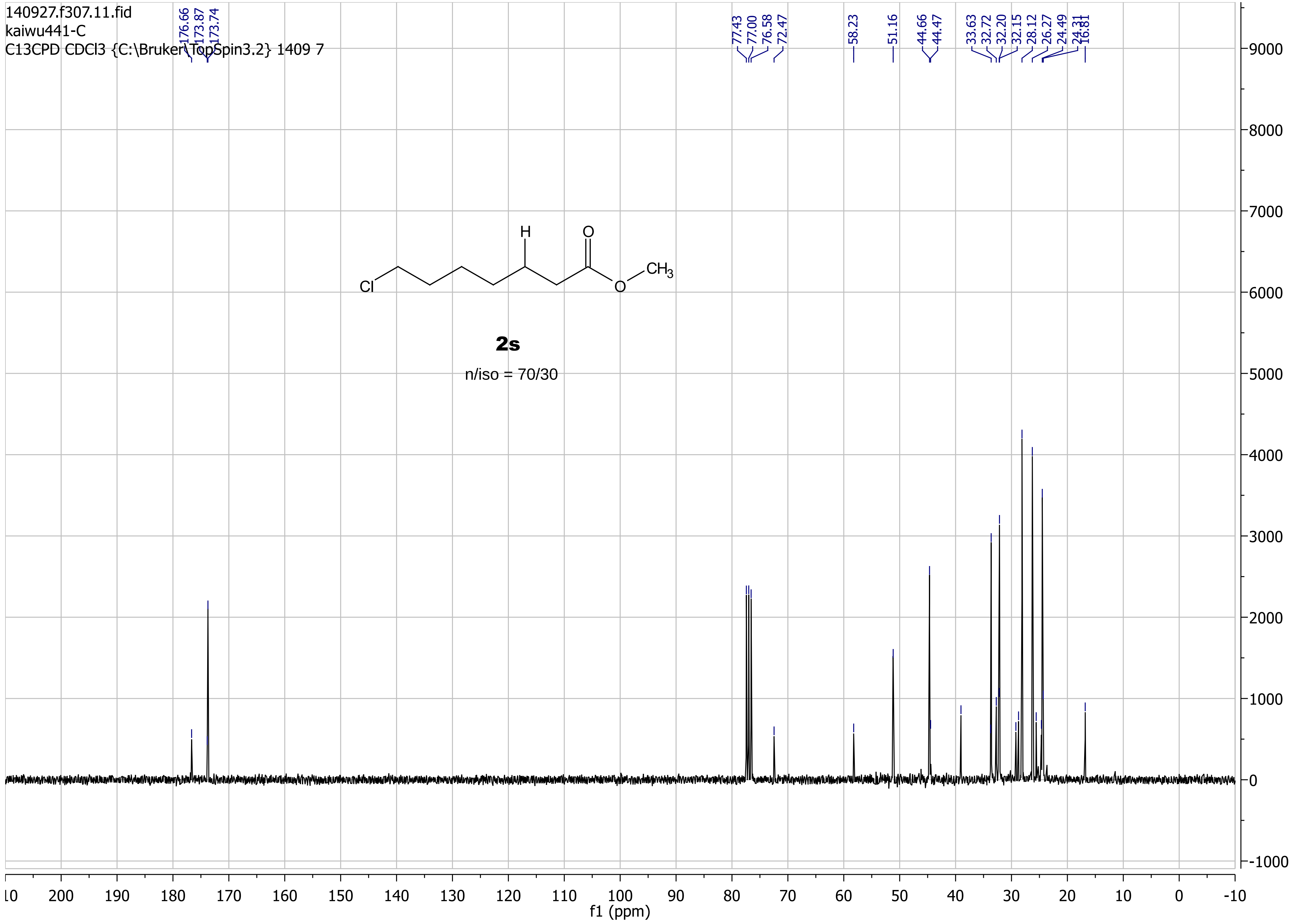
140927.f307.11.fid
kaiwu441-C
C13CPD CDCl3 {C:\Bruker\TopSpin3.2} 1409 7
176.66
173.87
173.74
77.43
77.00
76.58
72.47
58.23
51.16
44.66
44.47
33.63
32.72
32.20
32.15
28.12
26.27
24.49
24.31
16.81
Cl
H
O
O
CH3
2s
n/iso = 70/30
f1 (ppm)

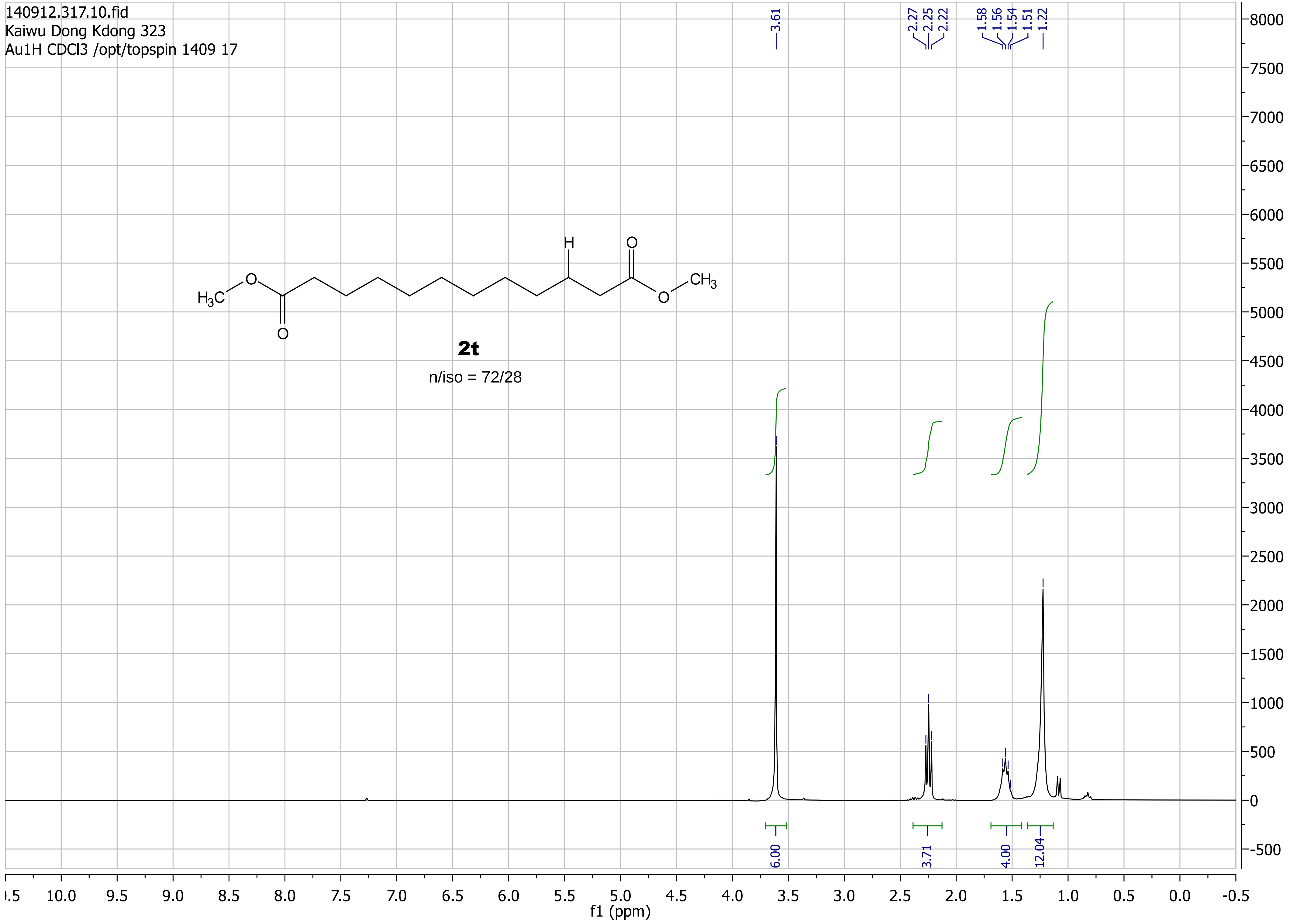

140912.317.10.fid
Kaiwu Dong Kdong 323
Au1H CDCl3 /opt/topspin 1409 17
3.61
2.27
2.25
2.22
1.58
1.56
1.54
1.51
1.22
2t
n/iso = 72/28
6.00
3.71
4.00
12.04
f1 (ppm)

140912.317.11.fid
Kaiwu Dong Kdong 323
Au13C CDCl3 /opt/topspin 1409 17

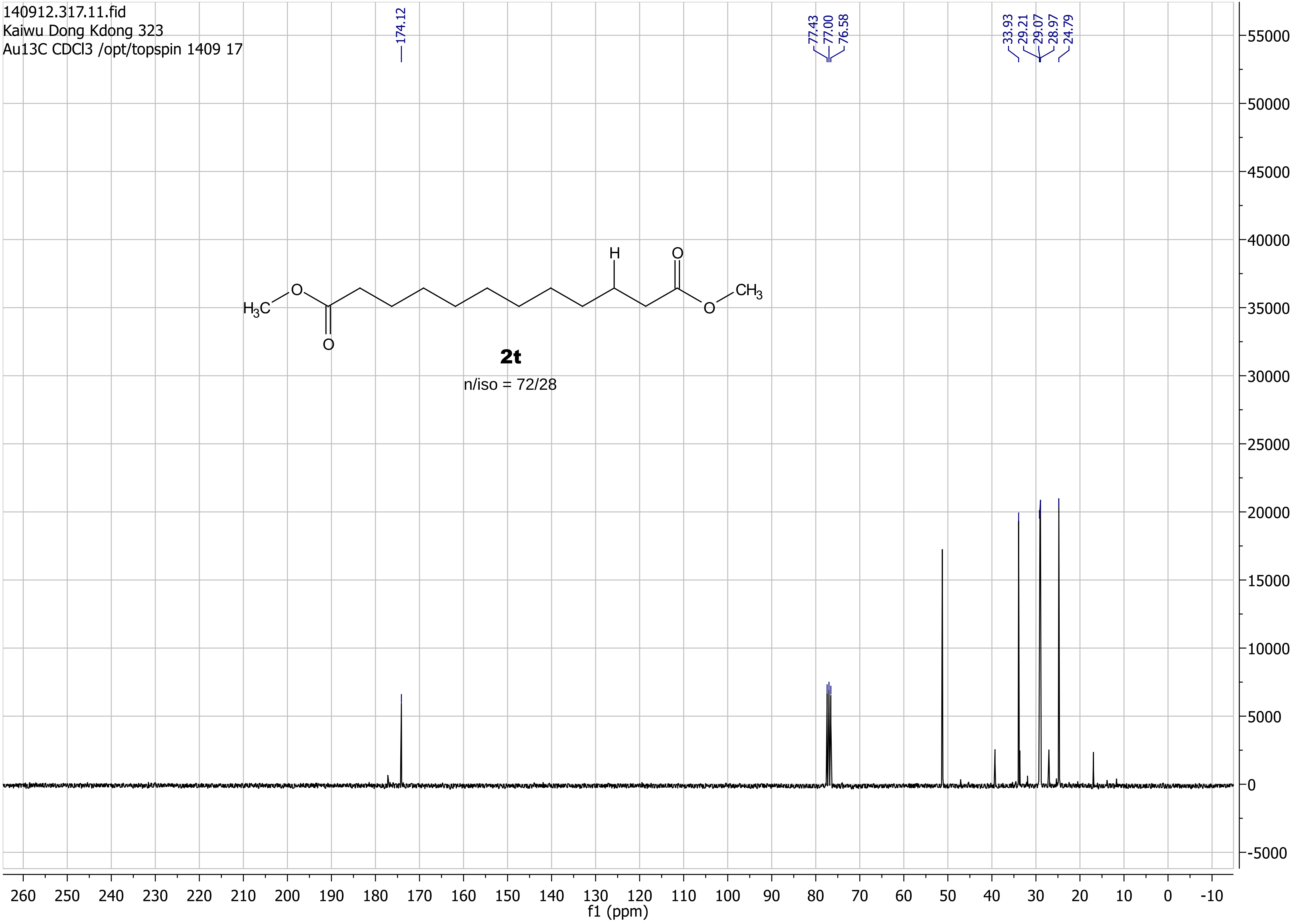

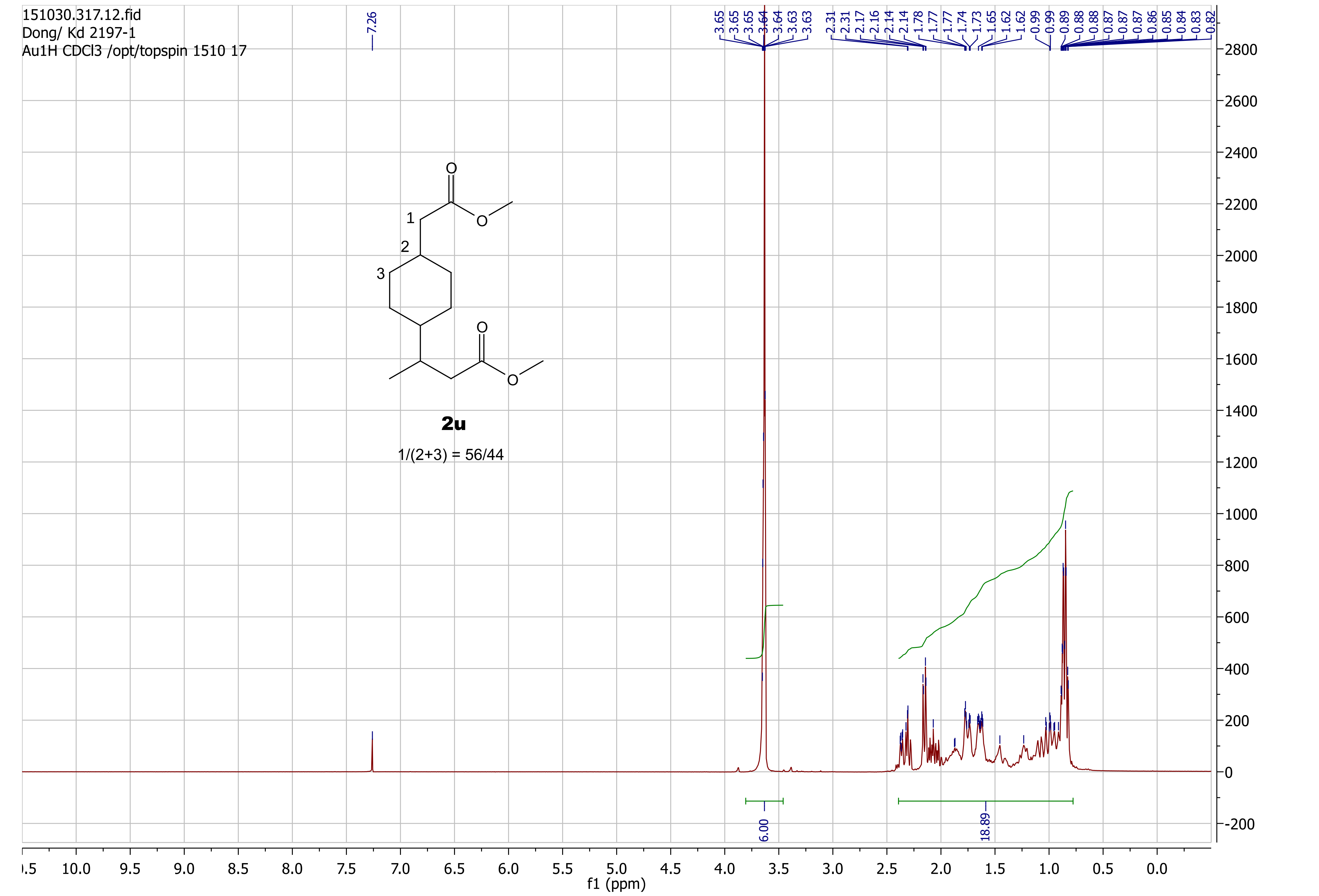
151030.317.12.fid
Dong/ Kd 2197-1
Au1H CDCl3 /opt/topspin 1510 17
7.26
3.65 3.65 3.65 3.64 3.64 3.63 3.63
2.31 2.31 2.17 2.16 2.14 2.14 1.78 1.77 1.77 1.74 1.73 1.65 1.62 1.62 0.99 0.99 0.89 0.88 0.88 0.87 0.87 0.87 0.86 0.85 0.84 0.83 0.82
O
O
1
2
3
O
O
2u
1/(2+3) = 56/44
6.00
18.89
f1 (ppm)

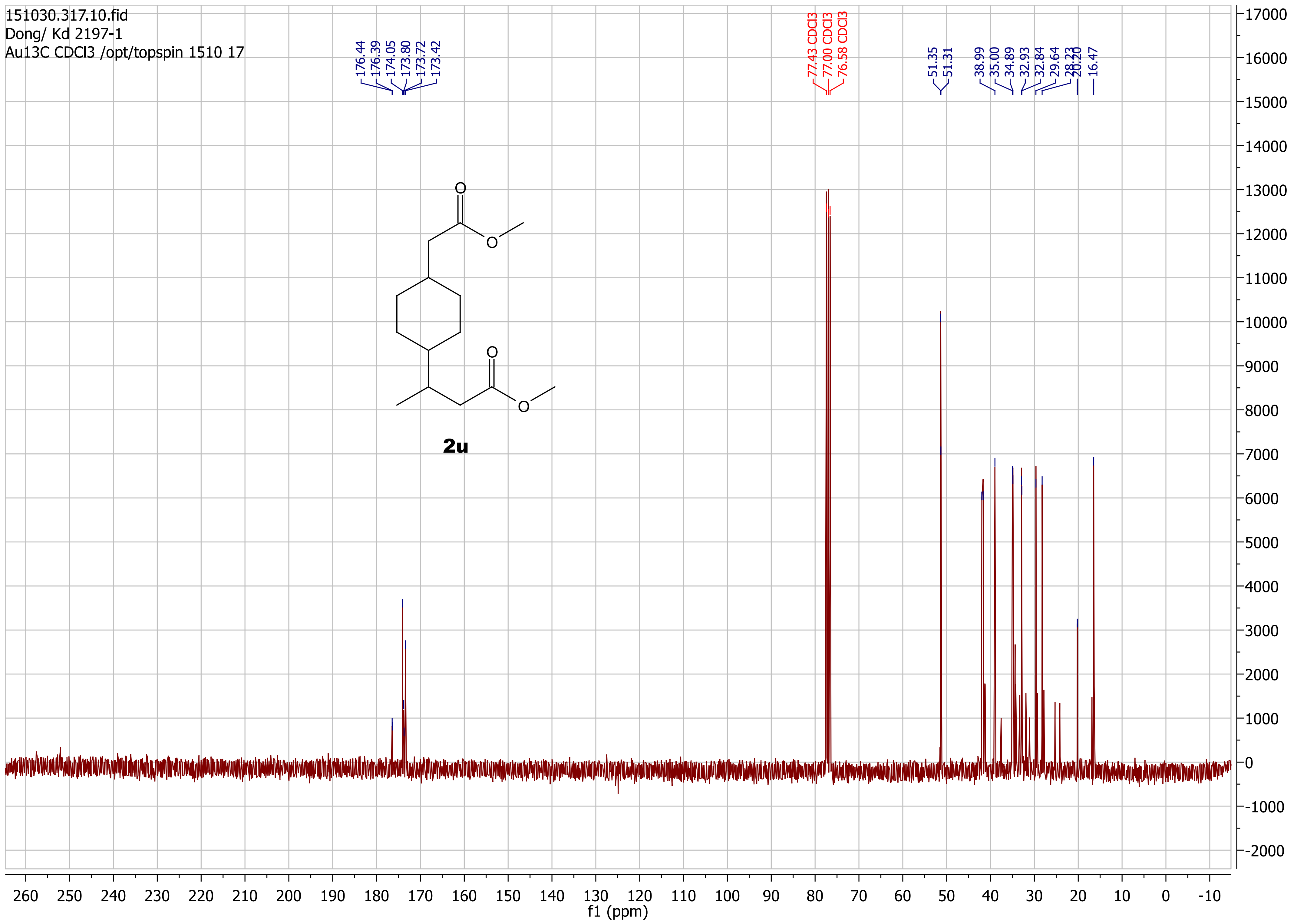
151030.317.10.fid
Dong/ Kd 2197-1
Au13C CDCl3 /opt/topspin 1510 17
176.44
176.39
174.05
173.80
173.72
173.42
77.43 CDCl3
77.00 CDCl3
76.58 CDCl3
51.35
51.31
38.99
35.00
34.89
32.93
32.84
29.64
28.23
20.20
16.47
O
O
O
O
2u
f1 (ppm)

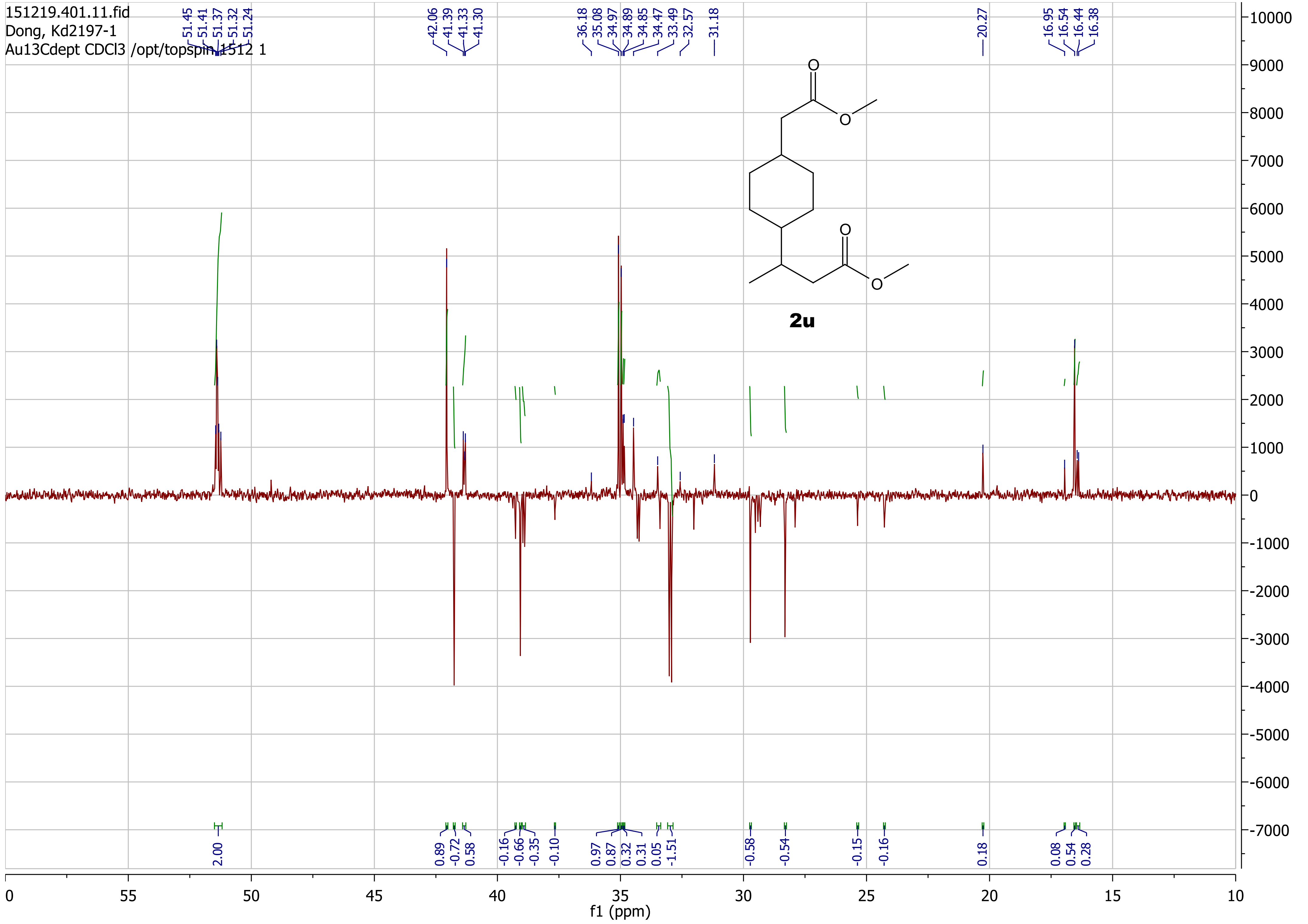
151219.401.11.fid
Dong, Kd2197-1
Au13Cdept CDCl3 /opt/topspin 1512 1
51.45
51.41
51.37
51.32
51.24
42.06
41.39
41.33
41.30
36.18
35.08
34.97
34.89
34.85
34.47
33.49
32.57
31.18
20.27
16.95
16.54
16.44
16.38
O
O
O
O
2u
2.00
0.89
-0.72
0.58
-0.16
-0.66
-0.35
-0.10
0.97
0.87
0.32
0.31
0.05
-1.51
-0.58
-0.54
-0.15
-0.16
0.18
0.08
0.54
0.28
f1 (ppm)

151103.f306.10.fid
Kaiwu Dong kd3077-1
PROTON CDCl3 {C:\Bruker\TopSpin3.2PL6} 1511 6

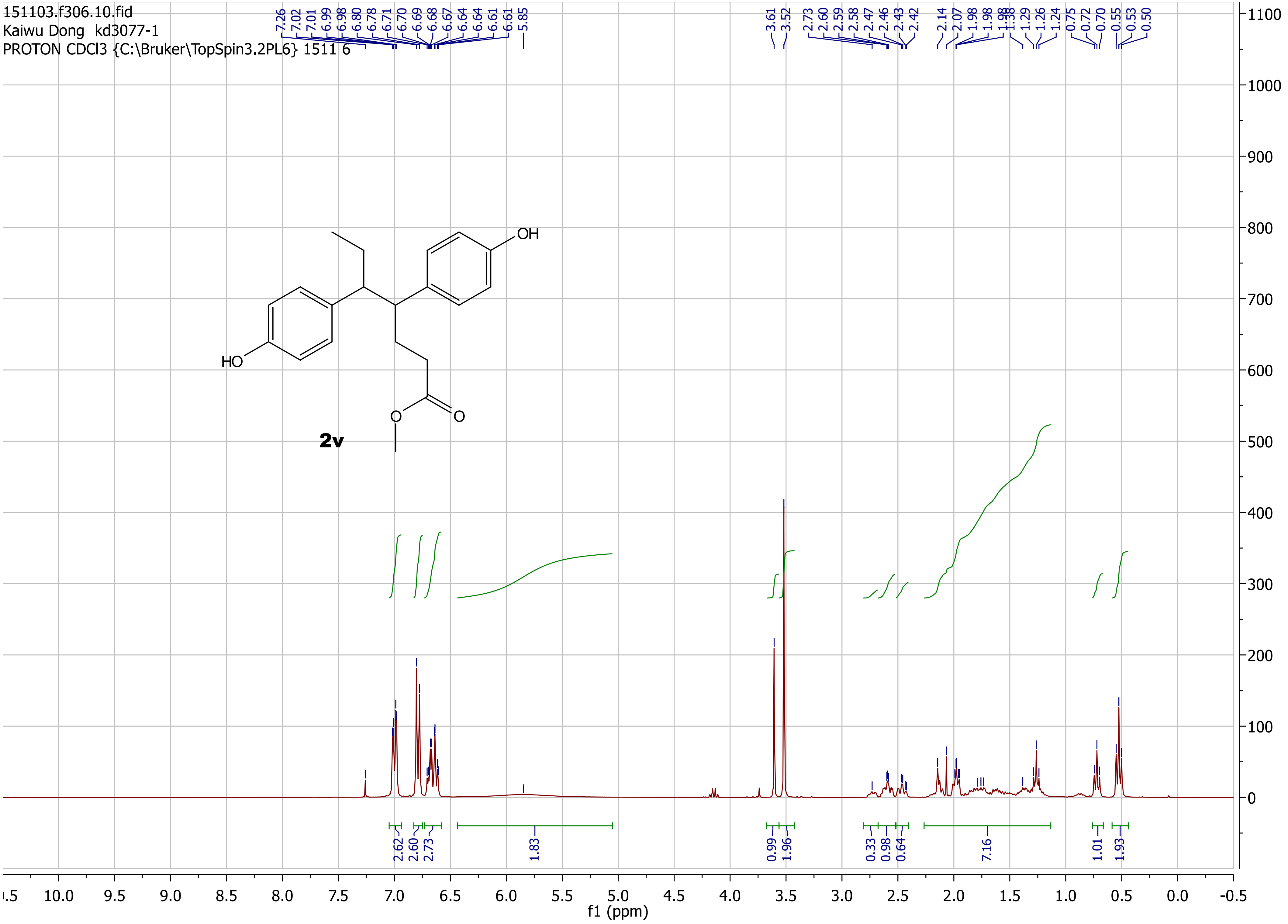

151103.f306.11.fid
Kaiwu Dong kd3077-1
C13CPD CDCl3 {C:\Bruker\TopSpin3.2PL6} 1511 6

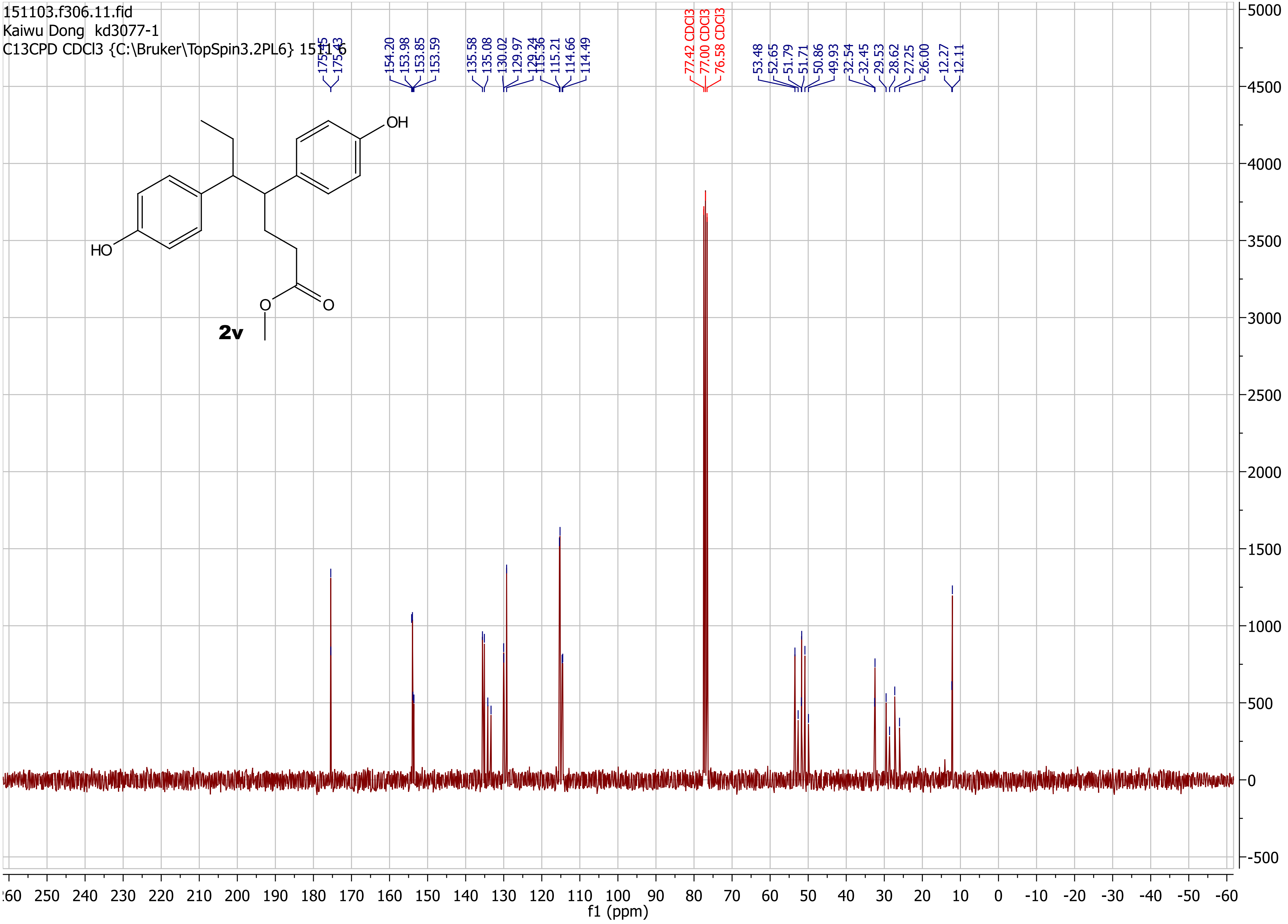

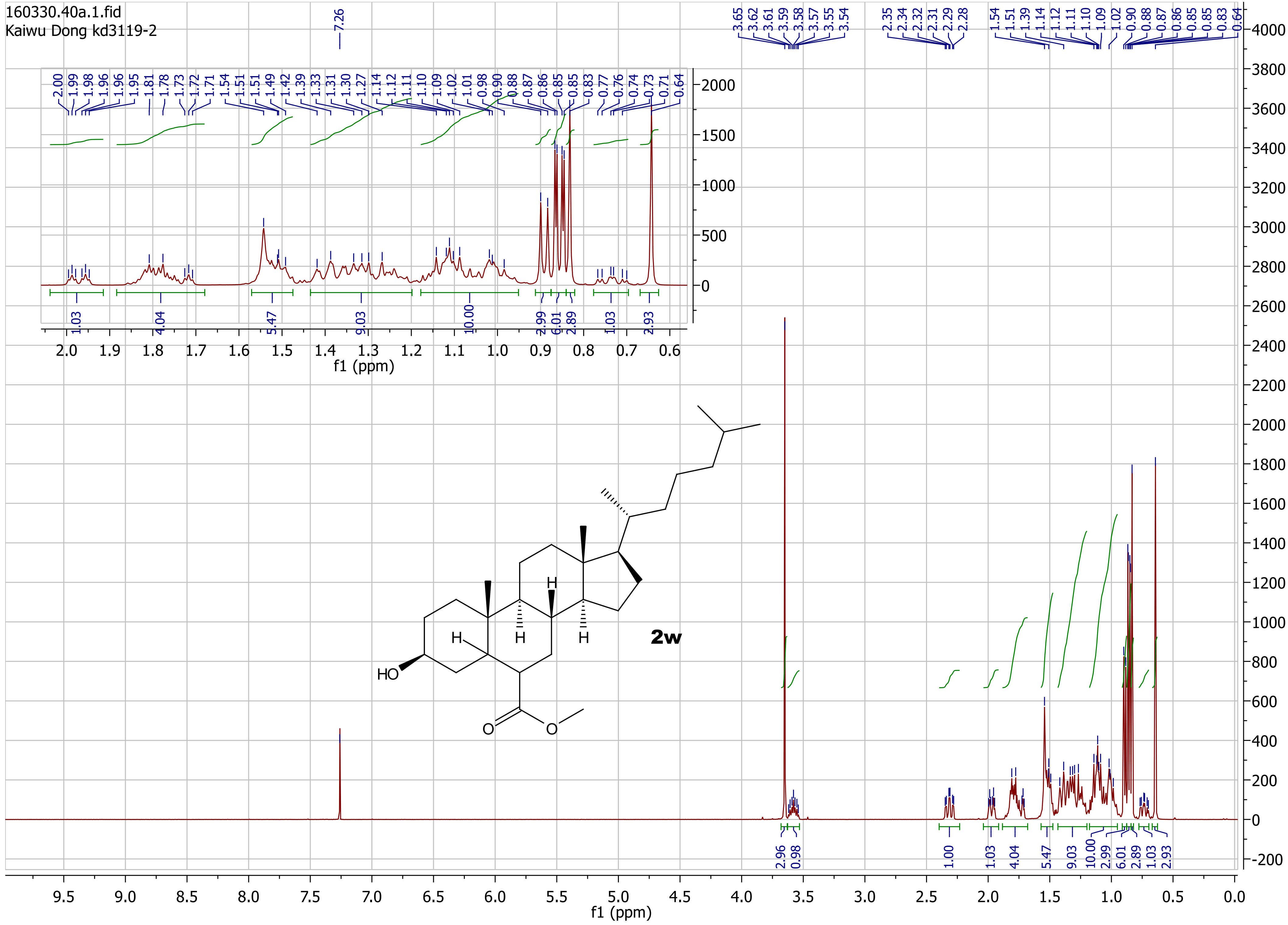

160330.40a.1.fid
Kaiwu Dong kd3119-2
2w
HO
O
O
H
H
H
H
f1 (ppm)
f1 (ppm)

160324.404.11.fid
Kaiwu Dong kd3119-2
Au13C CDCl3 /opt/topspin 1603 4

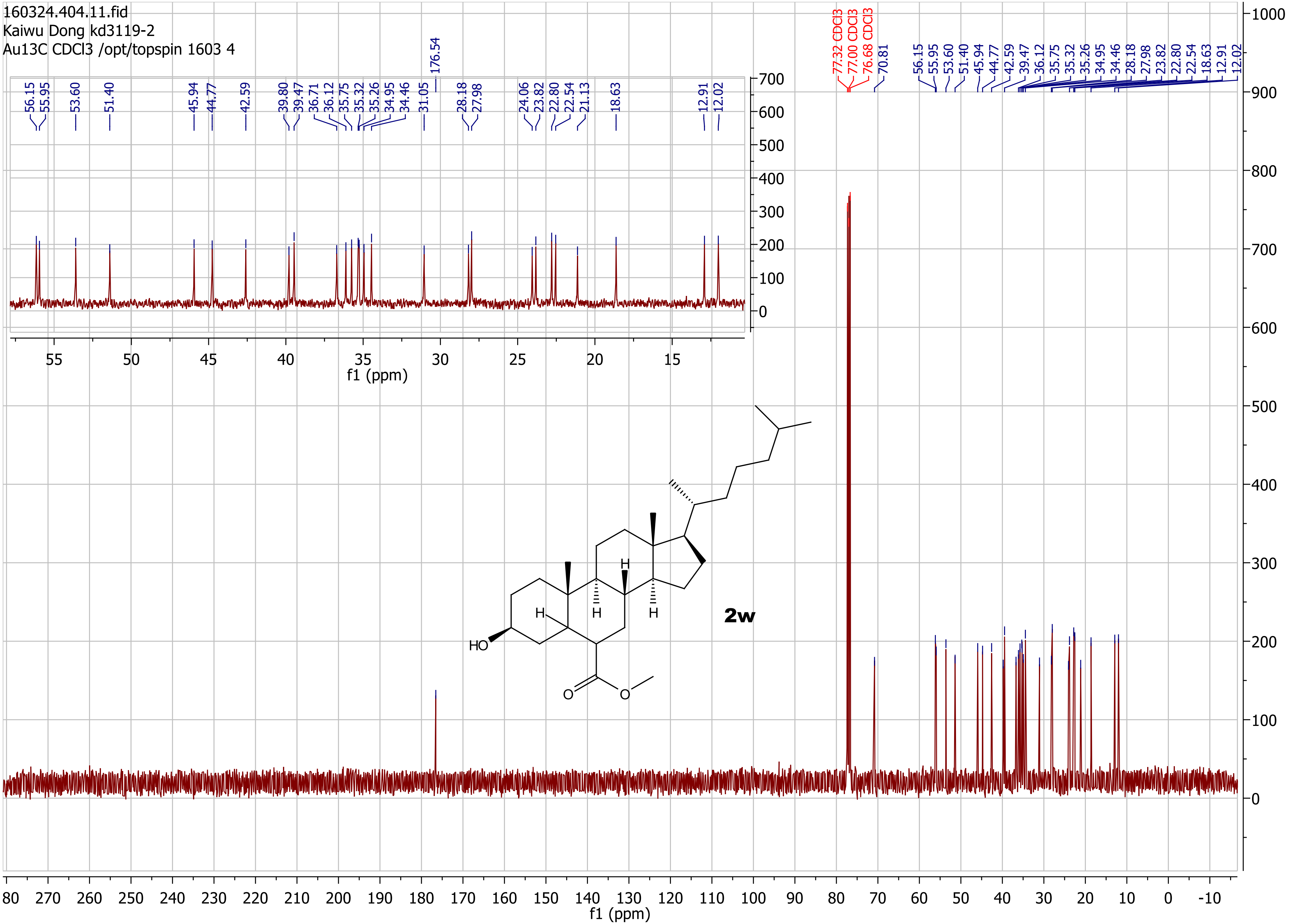

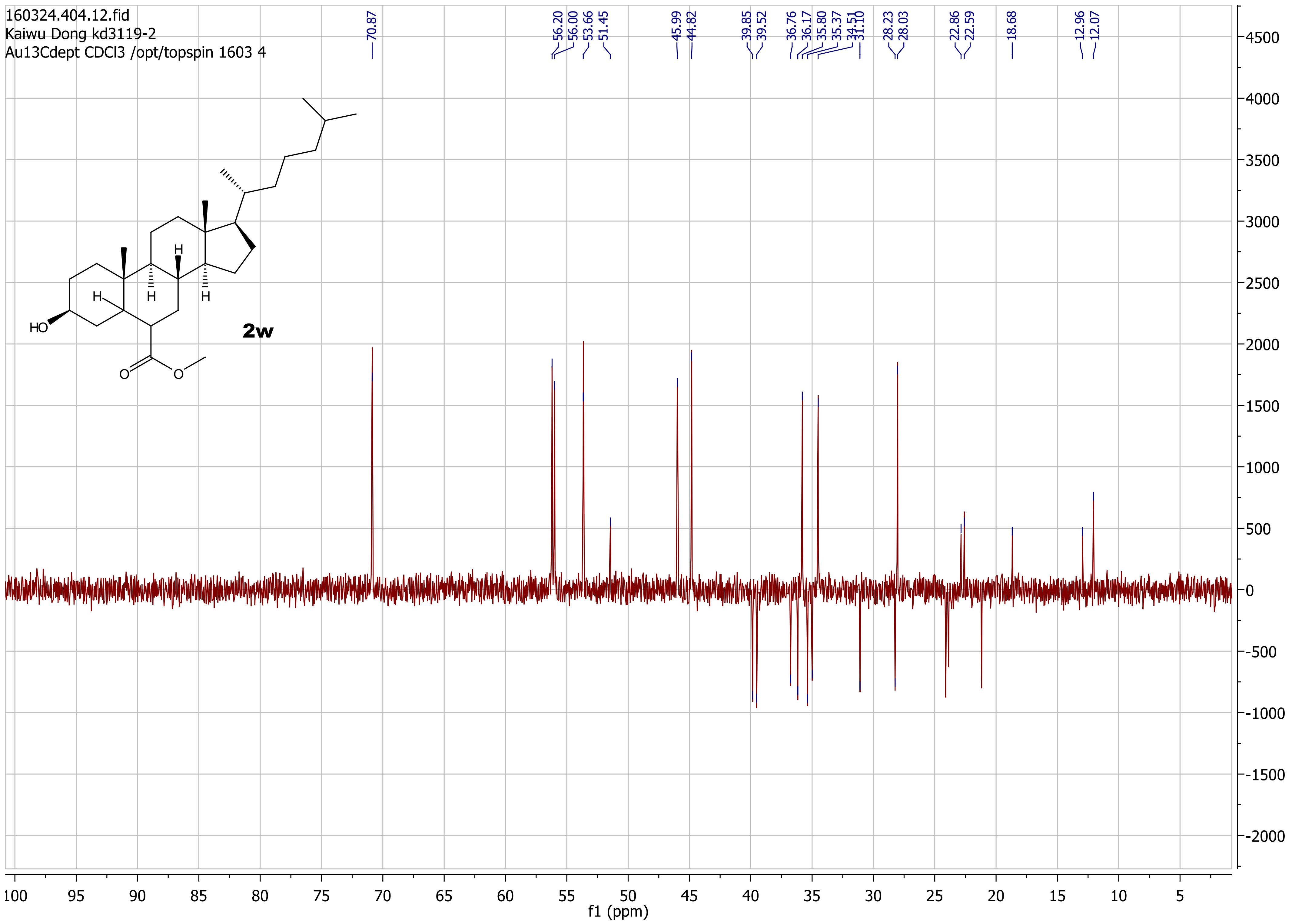

160324.404.12.fid
Kaiwu Dong kd3119-2
Au13Cdept CDCl3 /opt/topspin 1603 4
70.87
56.20
56.00
53.66
51.45
45.99
44.82
39.85
39.52
36.76
36.17
35.80
35.37
34.51
31.10
28.23
28.03
22.86
22.59
18.68
12.96
12.07
HO
H
O
2w
f1 (ppm)

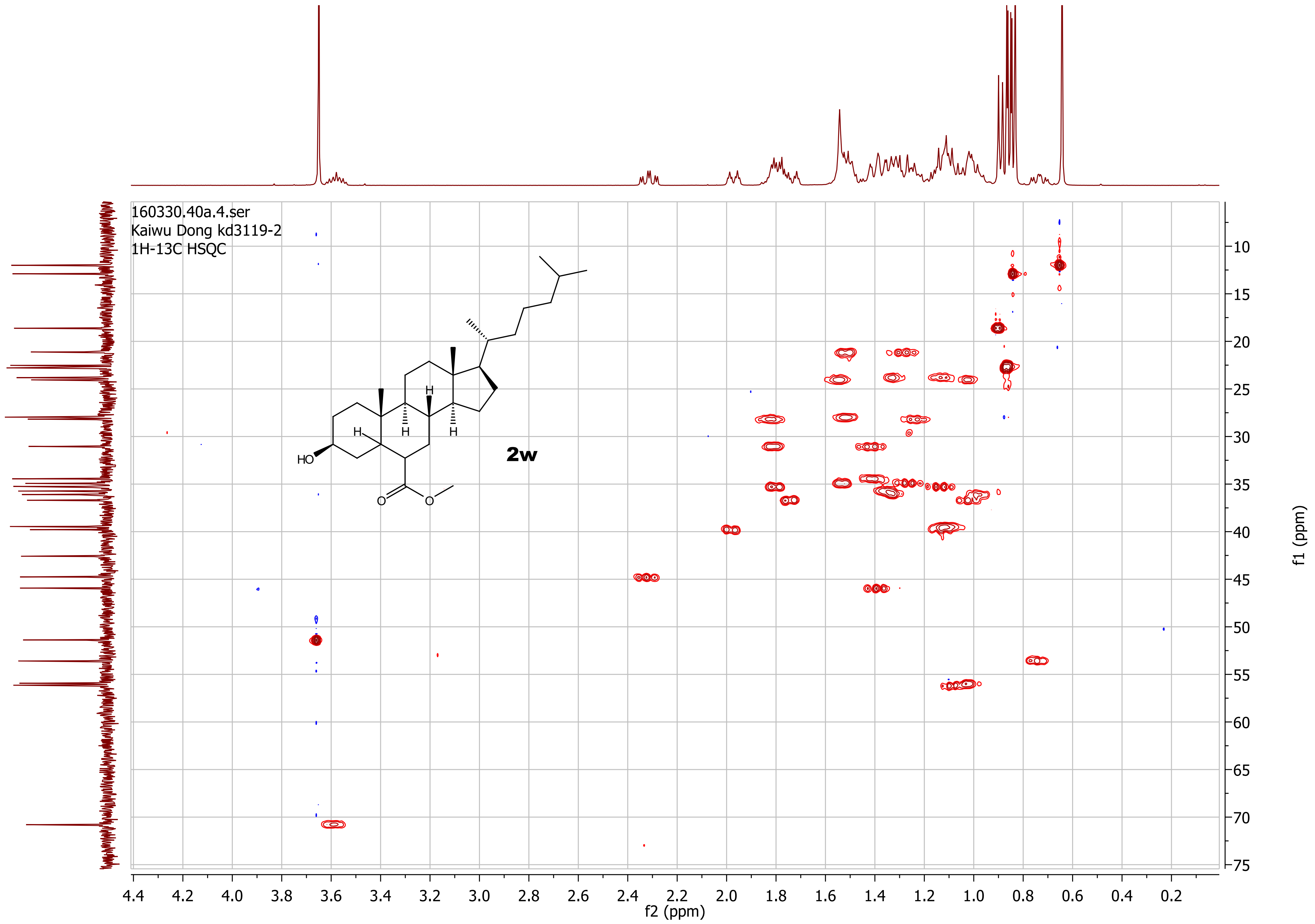
160330.40a.4.ser
Kaiwu Dong kd3119-2
1H-13C HSQC
HO
H
H
H
H
O
O
2w
f2 (ppm)
f1 (ppm)

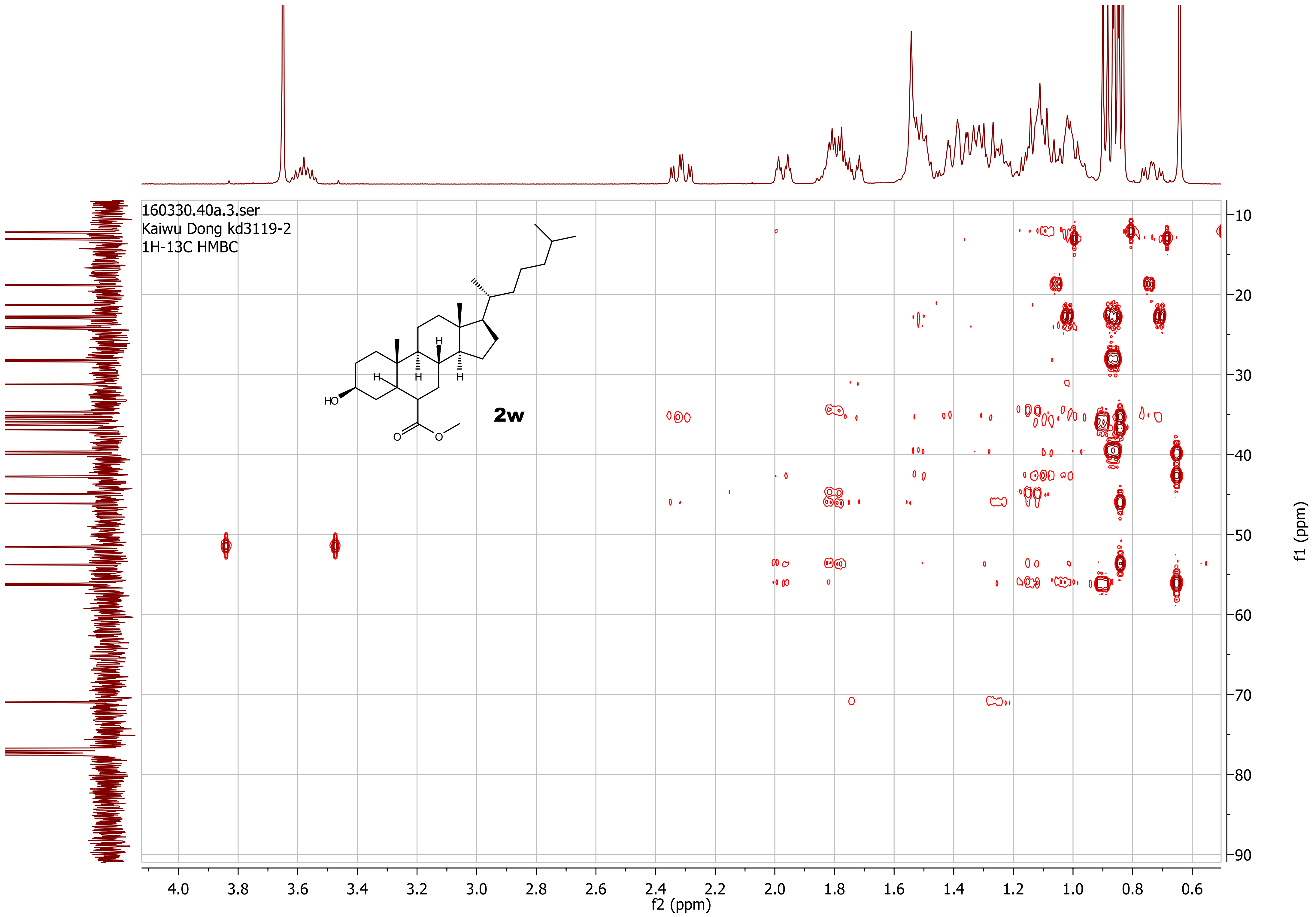
160330.40a.3.ser
Kaiwu Dong kd3119-2
1H-13C HMBC
HO
O
O
H
H
H
H
2w
f2 (ppm)
f1 (ppm)
4.0
3.8
3.6
3.4
3.2
3.0
2.8
2.6
2.4
2.2
2.0
1.8
1.6
1.4
1.2
1.0
0.8
0.6
10
20
30
40
50
60
70
80
90

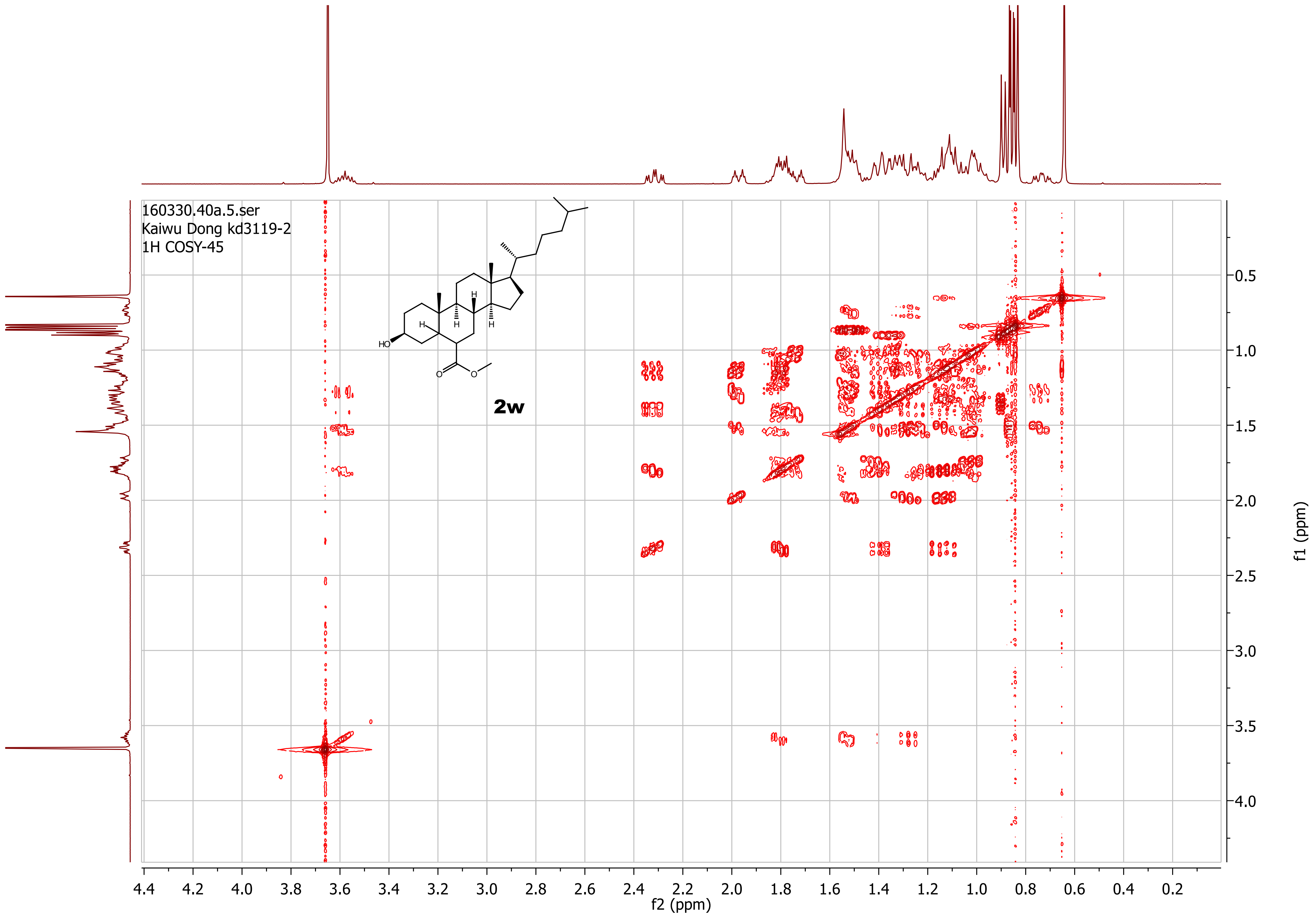

160330.40a.5.ser
Kaiwu Dong kd3119-2
1H COSY-45
HO
H
H
H
H
O
O
2w
f2 (ppm)
f1 (ppm)

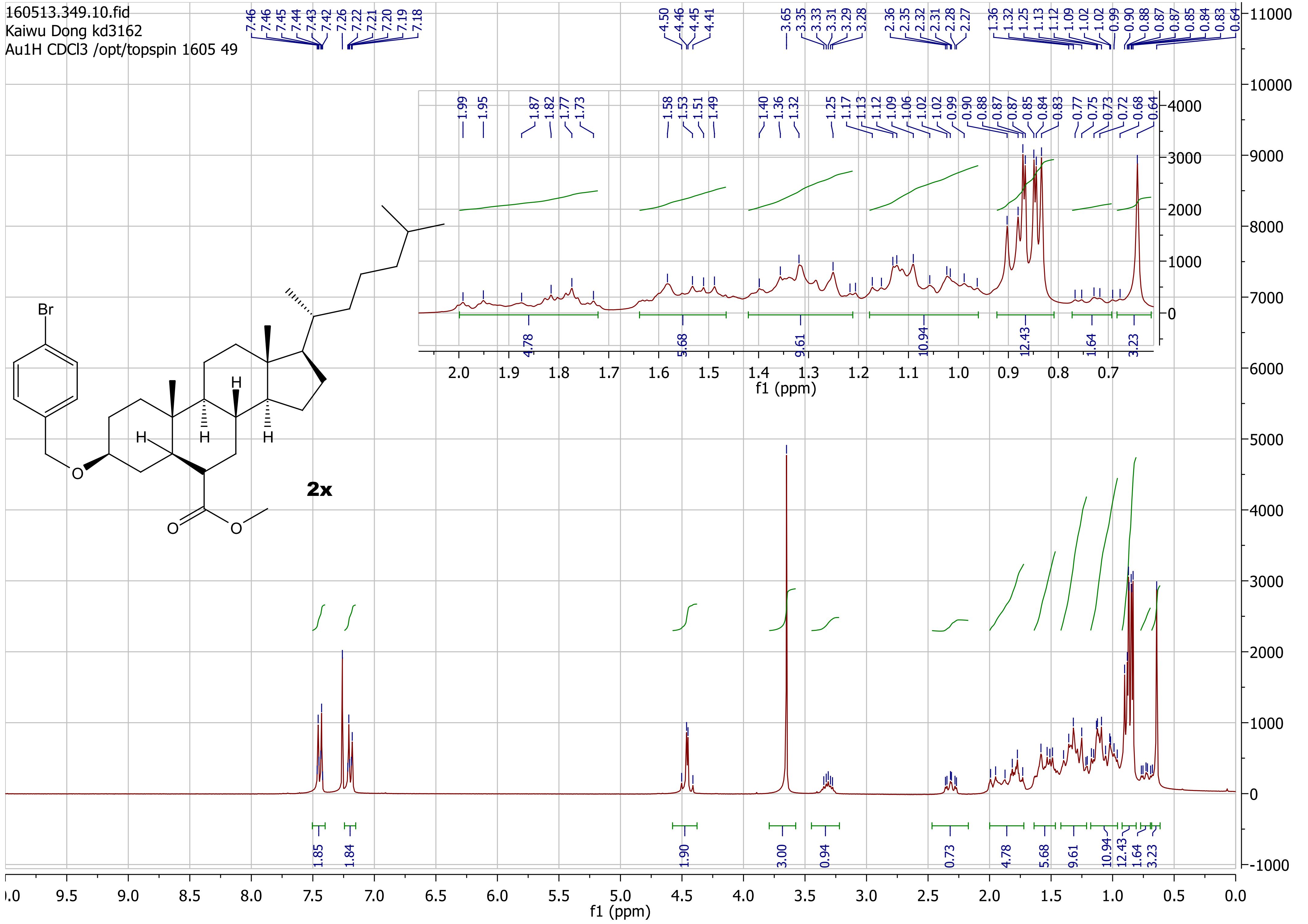
160513.349.10.fid
Kaiwu Dong kd3162
Au1H CDCl3 /opt/topspin 1605 49
2x
f1 (ppm)

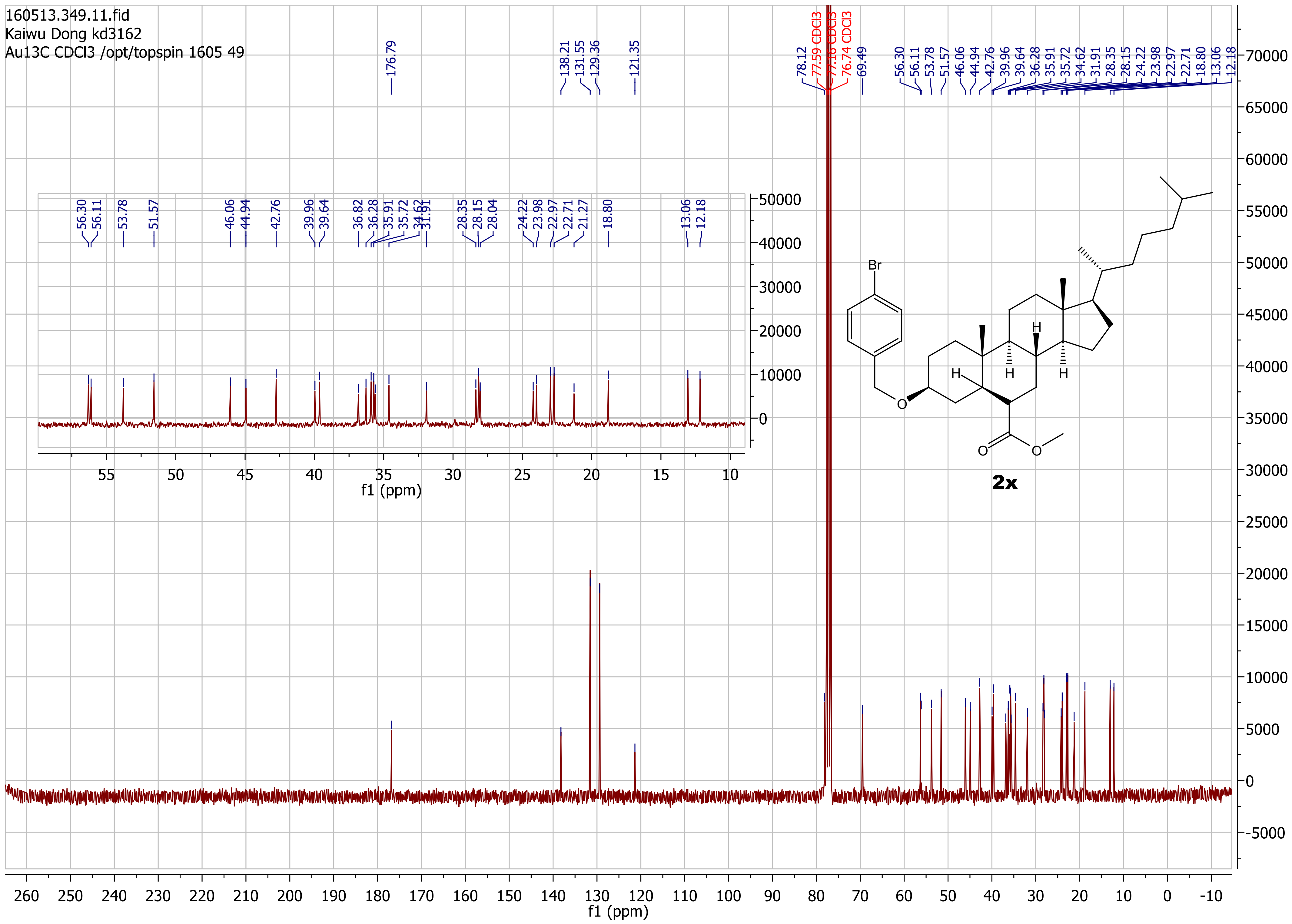
160513.349.11.fid
Kaiwu Dong kd3162
Au13C CDCl3 /opt/topspin 1605 49
176.79
138.21
131.55
129.36
121.35
78.12
77.59 CDCl3
77.16 CDCl3
76.74 CDCl3
69.49
56.30
56.11
53.78
51.57
46.06
44.94
42.76
39.96
39.64
36.82
36.28
35.91
35.72
34.62
31.91
28.35
28.15
28.04
24.22
23.98
22.97
22.71
21.27
18.80
13.06
12.18
Br
H
O
2x
f1 (ppm)

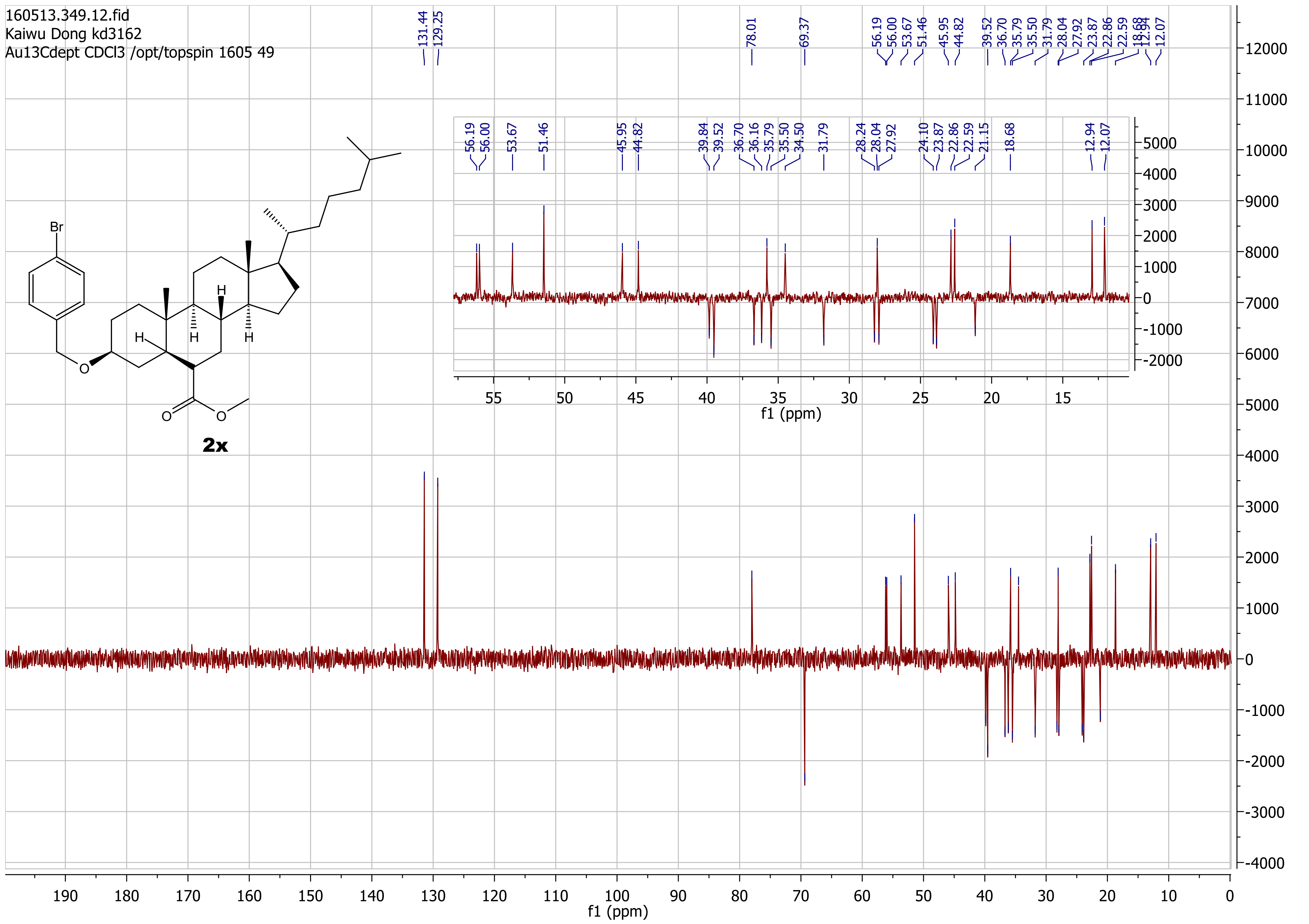

160513.349.12.fid
Kaiwu Dong kd3162
Au13Cdept CDCl3 /opt/topspin 1605 49
131.44
129.25
78.01
69.37
56.19
56.00
53.67
51.46
45.95
44.82
39.52
36.70
35.79
35.50
31.79
28.04
27.92
23.87
22.86
22.59
18.68
12.94
12.07
39.84
36.16
34.50
28.24
24.10
21.15
Br
H
O
2x
f1 (ppm)
