# Supplementary material for: Highly active and efficient catalysts for alkoxycarbonylation of alkenes
Source: Nat Commun. 2017 Jan 25;8:14117. doi: 10.1038/ncomms14117 (PMC5288498; doi:10.1038/ncomms14117)
Supplement: Peer review file [file ncomms14117-s2.pdf]

Editorial Note: This manuscript has been previously reviewed at another journal that is not operating a transparent peer review scheme. This document only contains reviewer comments and rebuttal letters for versions considered at Nature Communications.
